# Markers typed in genome-wide analysis identify regions showing deviation from Hardy-Weinberg equilibrium

**DOI:** 10.1186/1756-0500-2-29

**Published:** 2009-03-02

**Authors:** Anna E Vine, David Curtis

**Affiliations:** 1 Centre for Psychiatry, Barts and the London School of Medicine and Dentistry, London, E1 1BB, UK

## Abstract

**Background:**

Deviations from Hardy-Weinberg equilibrium (HWE) are commonly thought of as indicating genotyping errors, population stratification or some other artefact. However they could also arise through important biological mechanisms. In particular, genetic variants having a recessive effect on the successful fertilisation and/or development of an embryo might be manifest through such deviations in an unselected sample of "control" subjects.

**Findings:**

We investigated genotypes from 463842 autosomal markers from 1504 British subjects. We identified regions in which several neighbouring markers exhibited deviation from HWE in the same direction by considering "heterozygosity scores" in windows of 10 markers. The heterozygosity score for each marker was defined as -log(p) or log(p) according to whether the marker demonstrated increased heterozygosity or homozygosity. In each window the marker with the highest absolute score was ignored and the positive and negative scores were summed for the other nine markers. Windows were selected on the basis of this sum exceeding a given threshold, for which we used values of 50 or 15.

For the threshold of 50, we identified 7 regions with increased heterozygosity and for the threshold of 15 we identified 22 regions with increased heterozygosity, 23 with increased homozygosity and 2 containing both kinds of window. The most impressive of these results came from a group of 6 markers at 17q21, each of which showed increased heterozygosity significant at p < 10^-190^.

**Conclusion:**

The human genome contains regions which deviate markedly from HWE and these might harbour genes influencing embryonic survival.

## Findings

When marker allele frequencies in controls deviate markedly from Hardy-Weinberg equilibrium (HWE) this is commonly taken as an indicator that the genotyping is unreliable or that there is marked population stratification and the marker is discarded [1]. However if common polymorphisms influence embryonic survival then it is expected that these may also lead to such deviations. The existence of such loci is supported by a genome-wide tendency for siblings to share alleles more than would be expected by chance [2].

As previously suggested, we reasoned that if groups of nearby markers all showed deviation from HWE then this could not result purely from genotyping errors since there would be no reason for the same kind of error to be replicated in each marker [3]. Hence we used the control data from the 1958 British Birth Cohort which we obtained online from the Wellcome Trust Case-Control Consortium (WTCCC) after their approval was granted [4]. We used the genotypes called by the Chiamo algorithm and excluded those having either a studywise missing data proportion of more than 0.05 or a studywise minor allele frequency of less than 0.05 along with a studywise missing data proportion of more than 0.01. Naturally, for the purposes of this study, we did not exclude markers on grounds of deviation from HWE. Genotypes for 463842 autosomal markers were investigated, typed in 1504 subjects. We used sliding windows of ten markers across the sample and for each of the ten markers in each window we checked for deviation from HWE using a chi-squared test and recorded the resultant p-values. We assigned a "heterozygosity score" which was defined as -log_10 _(p) for markers showing increased heterozygosity (i.e. a positive number) and as log_10 _(p) for those showing increased homozygosity. We then excluded the marker having the highest absolute value for this score and considered only the scores from the other nine markers. The aim of the approach was to ignore regions where only a single marker produced a marked deviation from HWE but to identify those in which a group of markers all supported deviation in the same direction. We then summed all the positive heterozygosity scores and all the negative heterozygosity scores from the nine markers and tested whether the absolute value of either sum exceeded a predetermined threshold. For the current study, we used threshold values of 15 and of 50.

For each set of ten markers reaching the specified threshold using this process, we went on to investigate departure from HWE of two-marker and three-marker haplotypes using a method we have described elsewhere [5] to produce a one degree of freedom chi-squared test for departure from HWE, summarised by a "heterozygosity score" defined as -log_10 _(p) or log_10 _(p).

When there were overlapping sets of ten markers which exceeded the threshold they were amalgamated together, building up regions in which there was evidence for deviation from HWE. We obtained lists of genes within 200 kb either side of these regions by interrogating the UCSC genome browser [6].

Table 1, 2, 3, 4, 5, 6 show the results when we applied a threshold of 50 to identify sets of markers demonstrating deviation from HWE. (Additional File 1 is Table S5: HWETable5.doc and Additional File 2 is Table S6: HWETable6.doc. Results using a threshold of 15 are presented in Additional File 3 Table S7: HWETable7.doc.) Using the threshold of 50, 7 regions were identified as showing increased heterozygosity. In addition, there were 68 markers which individually produced results significant at p < 10^-50 ^but which were not supported by other markers nearby and hence which might represent genotyping errors, of which 10 demonstrated increased heterozygosity and 58 increased homozygosity. Using a threshold of 15 implicated 22 regions and 37 isolated markers as showing increased heterozygosity and 23 regions and 285 isolated markers as showing increased homozygosity. There were 2 regions containing a mixture of 10-marker windows meeting the criterion of 15 for both increased heterozygosity and homozygosity.

**Table 1:** Region of 1q31-41 with summed heterozygosity score exceeding 50.

**Marker or gene**	**Position**		**Marker genotype counts** **Observed** **Expected**			**Heterozygosity scores for single, two and three marker analysis**			**Gene description**
			**AA**	**AB**	**BB**	**1**	**2**	**3**	

CAMSAP1L1	198975309	Start							calmodulin regulated spectrin-associated protein 1-like 1

CAMSAP1L1	199096455	End							

GPR25	199108789	Start							G protein-coupled receptor 25

GPR25	199109874	End							

C1orf106	199127292	Start							chromosome 1 open reading frame 106

rs2792810	199146603		1468	0	12	-6.1	-6.1	-0.9	

			1456.1	23.8	0.1				

rs3767424	199148779		1480	0	0	0	-0.1	13.4	

			1480	0	0				

C1orf106	199151486	End							

C1orf81	199151855	Start							chromosome 1 open reading frame 81

rs805909	199160517		1143	312	25	-0.1	13.4	3.9	

			1140.1	317.7	22.1				

rs1819043	199163659		345	940	195	26.4	9	8.8	

			448.8	732.4	298.8				

rs3767421	199163817		382	753	345	0.3	0.3	8.7	

			388.7	739.5	351.7				

rs805911	199172076		1463	17	0	0	24.7	8.8	

			1463	16.9	0				

rs705736	199173739		361	933	186	25.4	9	8.2	

			462.7	729.7	287.7				

C1orf81	199202415	End							

KIF21B	199205143	Start							kinesin family member 21B

rs7522991	199223808		383	754	343	0.3	0.2	8.3	

			390.3	739.5	350.3				

rs6696611	199225346		395	742	343	0.1	8	7.9	

			396.5	739.1	344.5				

rs705747	199236940		366	936	178	26.5	26	8.7	

			470	728.1	282				

rs697455	199243733		1468	12	0	0	0.3	0.3	

			1468	12	0				

rs3767406	199243903		383	754	343	0.3	0.3	0	

			390.3	739.5	350.3				

rs497824	199256967		1468	12	0	0	0	0	

			1468	12	0				

KIF21B	199259451	End							

The table shows markers and genes in a region of 1q31-41 showing increased heterozygosity using a threshold for the summed heterozygosity scores (ignoring the highest-scoring marker) exceeeding 50. Observed counts are shown for each marker genotype with the expected counts in the row below. Heterozygosity scores, defined as -log(p) for increased heterozygosity and log(p) for increased homozygosity, are shown for individual markers and for two and three marker haplotypes.

**Table 2:** Region of 6p23-25.3 with summed heterozygosity score exceeding 50.

**Marker or gene**	**Position**		**Marker genotype counts** **Observed** **Expected**			**Heterozygosity scores for single, two and three marker analysis**			**Gene description**
			**AA**	**AB**	**BB**	**1**	**2**	**3**	

OR4F1P	50822	Start							olfactory receptor, family 4, subfamily F, member 1 pseudogene

OR4F1P	51956	End							

LOC646070	59339	Start							similar to capicua homolog

LOC646070	89509	End							

LOC100132266	89746	Start							similar to hCG2014367

LOC100132266	91534	End							

FLJ43763	148313	Start							hypothetical protein LOC642316

FLJ43763	148839	End							

rs6927090	197145		1456	24	0	0	0.2	0.2	

			1456.1	23.8	0.1				

rs12197235	197772		1129	333	18	0.3	0.2	0.7	

			1134	323	23				

rs2181107	214735		1456	24	0	0	0.8	2.6	

			1456.1	23.8	0.1				

rs734674	224695		1211	266	3	0.7	2.5	55.5	

			1220.5	247	12.5				

rs815583	230695		961	505	14	4	58.2	43.2	

			995	437	48				

DUSP22	237101	Start							dual specificity phosphatase 22

rs815593	239457		166	1151	163	100.6	69.6	59.8	

			371.5	740	368.5				

rs7754000	248017		1201	262	17	-0.1	0.2	0.3	

			1198.8	266.4	14.8				

SNP_A-4299501	260197		1322	158	0	0.3	0.8	69.7	

			1326.2	149.6	4.2				

rs12198312	268326		1071	381	28	0.2	69.5	68	

			1075.3	372.5	32.3				

rs11757245	273070		394	1072	14	86.9	80.2	79.5	

			584.4	691.2	204.4				

rs3800250	279825		1375	104	1	0.1	0.1	0.1	

			1375.9	102.2	1.9				

rs7763092	294386		1439	41	0	0	0.2	0.6	

			1439.3	40.4	0.3				

DUSP22	296355	End							

rs2671415	312109		1298	180	2	0.3	0.6	0.6	

			1301.7	172.6	5.7				

rs9501958	323970		1284	196	0	0.5	0.4	0	

			1290.5	183	6.5				

rs7745887	329546		1435	45	0	0	0	0	

			1435.3	44.3	0.3				

IRF4	336760	Start							interferon regulatory factor 4

IRF4	356193	End							

EXOC2	430138	Start							exocyst complex component 2

LOC727827	469180	Start							hypothetical protein LOC727827

LOC727827	470524	End							

LOC642335	481199	Start							hypothetical LOC642335

LOC642335	483632	End							

EXOC2	638109	End							

**Table 3:** Region of 8p23.3 with summed heterozygosity score exceeding 50.

**Marker or gene**	**Position**		**Marker genotype counts** **Observed** **Expected**			**Heterozygosity scores for single, two and three marker analysis**			**Gene description**
			**AA**	**AB**	**BB**	**1**	**2**	**3**	

KBTBD11	1909451	Start							kelch repeat and BTB (POZ) domain containing 11

KBTBD11	1942509	End							

MYOM2	1980565	Start							myomesin (M-protein) 2, 165 kDa

MYOM2	1980565	Start							myomesin (M-protein) 2, 165 kDa

MYOM2	2080787	End							

MYOM2	2080787	End							

rs1478960	2137223		1316	160	4	0	0.4	1	

			1316.8	158.5	4.8				

rs1382608	2151988		372	772	336	1	1.5	1.1	

			388.2	739.6	352.2				

rs7838658	2152119		688	658	134	0.6	0.8	0.9	

			698.8	636.3	144.8				

rs2127175	2188481		1272	203	5	0.2	0.2	-8.5	

			1274.7	197.7	7.7				

rs1037704	2189117		1418	62	0	0.1	-10.6	0	

			1418.6	60.7	0.6				

rs2607684	2189919		753	509	218	-11.7	-0.1	-0.3	

			685.8	643.3	150.8				

rs10111921	2200103		644	760	76	10.9	4.9	36.8	

			708.5	631	140.5				

rs2618872	2218978		1161	302	17	0.1	44.4	80.4	

			1163.1	297.9	19.1				

rs2605037	2219069		456	971	53	49.5	98.1	71.4	

			598.9	685.1	195.9				

rs2013135	2226740		278	1148	54	107.5	80.5	53.4	

			490.5	723	266.5				

rs315225	2231722		510	901	69	31.6	24.4	20.3	

			623.4	674.3	182.4				

rs7015044	2234017		513	834	133	13	10	17	

			584.4	691.2	204.4				

rs1159923	2234501		1197	274	9	0.3	19.7	19	

			1202.4	263.2	14.4				

rs931093	2243578		599	767	114	7.6	8.2	24.2	

			652.2	660.5	167.2				

rs2605035	2257735		1401	79	0	0.1	16.6	0.2	

			1402.1	76.9	1.1				

rs6558636	2257979		589	814	77	16.8	0	-2.7	

			670.3	651.4	158.3				

rs6558637	2258129		594	534	352	-21.5	-8.5	-5.9	

			500.9	720.2	258.9				

rs4876160	2258259		907	498	75	-0.2	0.3	0.1	

			902.9	506.1	70.9				

rs1614403	2258689		1145	325	10	0.7	0	0	

			1155.1	304.8	20.1				

rs2260185	2259833		1411	69	0	0.1	14.9	0	

			1411.8	67.4	0.8				

rs11136507	2259919		663	767	50	15.3	0	0	

			740	613.1	127				

**Table 4:** Region of 17q21 with summed heterozygosity score exceeding 50.

**Marker or gene**	**Position**		**Marker genotype counts** **Observed** **Expected**			**Heterozygosity scores for single, two and three marker analysis**			**Gene description**
			**AA**	**AB**	**BB**	**1**	**2**	**3**	

LOC339192	40652465	Start							hypothetical protein LOC339192

FMNL1	40655075	Start							formin-like 1

LOC339192	40675042	End							

FMNL1	40680468	End							

C17orf46	40687543	Start							chromosome 17 open reading frame 46

C17orf46	40695262	End							

MAP3K14	40696271	Start							mitogen-activated protein kinase kinase kinase 14

MAP3K14	40750197	End							

rs4792855	40815480		405	746	329	0.2	0.3	0.3	

			409	738	333				

rs1230094	40825939		751	622	107	0.6	0.7	0.6	

			762.1	599.9	118.1				

rs732589	40826543		762	617	101	0.7	0.6	0.5	

			774.3	592.4	113.3				

ARHGAP27	40827058	Start							Rho GTPase activating protein 27

rs1230103	40841574		753	619	108	0.5	0.5	0.2	

			762.8	599.5	117.8				

rs12947718	40848884		977	458	45	0.3	0	0	

			982.7	446.5	50.7				

ARHGAP27	40858780	End							

LOC201175	40862501	Start							hypothetical protein LOC201175

LOC201175	40867570	End							

PLEKHM1	40869049	Start							pleckstrin homology domain containing, family M (with RUN domain) member 1

rs17631303	40872185		1036	399	45	-0.2	0	-0.1	

			1031.4	408.2	40.4				

rs3946526	40897439		980	455	45	0.3	0.1	49.4	

			985.2	444.7	50.2				

rs2078200	40897617		803	573	104	0	80.1	79.7	

			802	574.9	103				

PLEKHM1	40923893	End							

LOC644354	40934084	Start							similar to Apoptosis-related protein 2 (APR-2)

LOC644354	40934428	End							

LRRC37A4	40939890	Start							leucine rich repeat containing 37, member A4 (pseudogene)

LRRC37A4	40948305	End							

rs2696639	41006823		89	1312	79	193.6	193.6	191.3	

			375	740	365				

rs2696640	41007016		88	1312	80	193.6	192	192	

			374	740	366				

rs2693363	41007205		93	1307	80	190.3	192.6	192.6	

			376.5	739.9	363.5				

rs2693364	41007294		87	1314	79	195	195	153.4	

			374	740	366				

rs2693371	41011471		87	1314	79	195	153.4	153.4	

			374	740	366				

rs17642476	41012163		1298	182	0	0.4	153.4	75.8	

			1303.6	170.8	5.6				

rs2463520	41015138		87	1314	79	195	98.8	98.8	

			374	740	366				

LOC644157	41018375	Start							similar to dead end homolog 1

The most convincing evidence for a real departure from HWE occurs at 17q21 in the region around rs2693363, as shown in Table 4. This marker and five others flanking it are each individually significant at p < 10^-190^. It does not seem plausible that this result could occur through a set of genotyping errors or through some other artefact and so we can only conclude that there really is a marked excess of heterozygosity in this region. Using the threshold of 50, it seems unlikely that any of the results could have occurred by chance. Perhaps the least convincing result is at 6p25.3 (Table 2), where rs815593 had a heterozygosity score of 100.6 and rs11757245 has a score of 86.9. No other markers nearby support deviation from HWE and one could argue that it is possible that the result for each marker is due to genotyping error and that it is mere coincidence that the two happen to lie close to each other. When the threshold is set as low as 15, we expect that a number of the results might have occurred by chance. Given that results from nearby markers are not independent, it is possible that a region might happen to show deviation from HWE at p < 10^-3 ^or p < 10^-4 ^and that several markers in this region might be significant at this level and hence produce a combined score exceeding the threshold of 15. On the other hand, many regions produced a score far in excess of this and a substantial proportion of regions identified using this lower threshold are likely to represent a real biological effect.

With regard to the comparison of single marker and haplotype-based analyses, there were no regions in which there was a haplotype analysis which provided stronger evidence for increased heterozygosity than the most significant single marker analysis. We would take this to indicate that the information supporting departure from HWE was captured by the single marker. For example, given the allele frequencies of rs11757245 at 6p25.3 (Table 2), one would expect 204.4 subjects to have genotype BB. In fact, this genotype occurs in only 14 subjects, a finding consistent with this polymorphism itself or one in close LD with it having a marked effect on survival. However when we considered regions in which there was a deviation towards excess homozygosity rather than heterozygosity, identified using the threshold of 15, there were a few for which a haplotype analysis was more significant than any single marker analysis. One interpretation of this might be that there could be an untyped polymorphism in LD with one or more of the haplotypes. For example, the frequencies at rs649022 at 4q26 of both the AA and BB genotypes are somewhat increased from HWE with p < 10^-12 ^(Additional File 3 Table S7). When the haplotypes of this marker are considered along with the next two markers, rs594125 and rs11726138, the deviation in favour of increased homozygosity is significant at p < 10^-20^. Inspection of the counts of haplotype combinations revealed that the haplotypes BAB and AAB were homozygous approximately twice as often as would be expected under HWE, with expected counts of 48.6 and 27.5 and observed counts of 83 and 59, respectively.

For most of the regions implicated there were a number of different genes within 200 kB, making it impossible to draw firm conclusions about which might harbour biologically meaningful polymorphisms. It would be difficult to avoid making subjective judgements about the relative weight given to statistical evidence and to biological plausibility. For example, a number of markers around rs1326581 at 6p12.2 combined to provide relatively weak statistical evidence for increased homozygosity (only just exceeding the threshold of 15, Additional File 3 Table S7), yet these markers span PKHD1, the gene for polycystic kidney and hepatic disease, mutations in which are a known cause of autosomal recessive kidney disease (ARKD) which can result in stillbirth or death in infancy or childhood. By contrast, as we have already noted there is extremely strong statistical evidence to support increased homozygosity around rs2693363 at 17q21.31 (Table 4) but none of the identified genes in the region are really obvious candidates to have a recessive lethal effect.

One indication for genes having a biologically significant role in influencing departures from HWE might be that similar genes were found in different implicated regions. There were several possible examples of this phenomemon which were apparent when the threshold of 15 was considered, as shown in Additional File 3 Table S7. Two cytogenetically distinct implicated regions contain CSMD1 and CSMD3, the genes for CUB and Sushi multiple domains 1 and 3, although the third gene of the family, CSMD2, did not occur in an implicated region. Three loci related to ribosomal protein S26 were in separated implicated regions: LOC728937 (similar to 40S ribosomal protein S26), RPS26P3 (ribosomal protein S26 pseudogene 3) and LOC644191 (40S ribosomal protein S26). However the gene for ribosomal protein S26 itself, RPS26, was not in an implicated region and nor was RPS26L1 (ribosomal protein S26-like 1). Two loci related to FMR1 were in separate implicated regions: NUFIP1P (nuclear fragile × mental retardation protein interacting protein 1 pseudogene) and CYF1P1 (cytoplasmic FMR1 interacting protein 1), although NUF1P1 and CYF1P2 were not. Three loci related to golgin subfamily a were in different implicated regions: LOC643707 (golgi autoantigen, golgin subfamily a, 6 pseudogene), LOC192130 (golgi autoantigen, golgin subfamily a, 4 pseudogene) and LOC729786 (similar to golgi autoantigen, golgin subfamily a, 8A). However the UCSC browser lists loci containing the phrase "golgin subfamily a" in 11 other regions which did not show departure from HWE. Finally, olfactory receptor genes and/or pseudogenes were found in four different implicated regions but there are over 400 of these distributed in a number of genomic regions.

This simple exploratory analysis clearly demonstrates that there are regions of the human genome which deviate markedly from HWE in a sample of unselected British adults. The evidence is stronger for some regions than for others and we have not attempted to quantify this on the basis of a formal statistical test. The nature of our approach means that we have only sought to identify regions in which the effect is apparent in more than one marker. It is quite likely that at least some of the single markers showing deviation from HWE which we have ignored do so because of a real effect rather than through genotyping error although we note that they more often showed increased homozygosity whereas the regions implicated by groups of markers showed more marked deviations towards heterozygosity. This may suggest that a substantial proportion of these isolated markers do represent genotyping errors. Likewise, the marker set we have used does not provide 100% coverage of the genome. Hence there may be many more regions of HWE present than those highlighted by the present study.

Although it seems clear that deviations from HWE exist, the mechanisms driving this are not clear. One proposal we made is that a recessive lethal polymorphism could lead to decreased homozygosity in surviving subjects. Such a polymorphism might cause death antenatally or in childhood or might prevent successful fertilisation. We argue that the effect on reproductive fitness of the parent would be minimal if it produced very early termination or prevented fertilisation. Nevertheless, such polymorphisms would need to be very common indeed if they were to be detectable in a sample size of only 1504, as we have used.

Although the best implicated regions demonstrate increased heterozygosity, we also find regions with increased homozygosity, some with p values less than 10^-10 ^or 10^-20 ^or even smaller. Theoretically, increased homozygosity could occur through the presence of deletions or population stratification but is seems hard to conceive that these mechanisms could produce an effect of such magnitude.

To conclude, we have obtained good evidence that some regions of the human genome demonstrate deviation from HWE in an unselected sample of adults from the UK population. We believe that these preliminary findings warrant further exploration.

## Competing interests

The authors declare that they have no competing interests.

## Authors' contributions

DC conceived the project. AEV carried out the analyses. Both contributed to the preparation of the manuscript. Both authors have read and approved the final manuscript

## Supplementary Material

Additional file 1 **HWETable5.doc**. Table 5. Region of 14q11.1-11.2 with summed heterozygosity score exceeding 50. Click here for file

Additional file 2 **HWETable6.doc**. Table 6. Region of 15q11-14 with summed heterozygosity score exceeding 50. Click here for file

Additional file 3 **HWETable7.doc**. Table 7. Regions with absolute summed heterozygosity score exceeding 15. Click here for file
