# Supplementary material for: Markers typed in genome-wide analysis identify regions showing deviation from Hardy-Weinberg equilibrium
Source: BMC Res Notes. 2009 Mar 2;2:29. doi: 10.1186/1756-0500-2-29 (PMC2667528; doi:10.1186/1756-0500-2-29)
Supplement: Additional file 1 — HWETable5.doc. Table 5. Region of 14q11.1-11.2 with summed heterozygosity score exceeding 50. [file 1756-0500-2-29-S1.doc]

**Markers typed in genome-wide analysis identify regions showing deviation from Hardy-Weinberg equilibrium.**

AE Vine, D Curtis

**Additional file**

**Table 5.** Region of 14q11.1-11.2 with summed heterozygosity score exceeding 50.

| **Marker or gene** | **Position** |  | **Marker genotype counts**  **Observed**  **Expected** | | | **Heterozygosity scores for single, two and three marker analysis** | | | **Gene description** |
| --- | --- | --- | --- | --- | --- | --- | --- | --- | --- |
|  |  |  | **AA** | **AB** | **BB** | **1** | **2** | **3** |  |
| P704P | 19057551 | Start |  |  |  |  |  |  | prostate-specific P704P |
| P704P | 19090272 | End |  |  |  |  |  |  |  |
| LOC642850 | 19091205 | Start |  |  |  |  |  |  | similar to positive cofactor 2, glutamine/Q-rich-associated protein isoform b |
| LOC642850 | 19144901 | End |  |  |  |  |  |  |  |
| NEK2P | 19166779 | Start |  |  |  |  |  |  | NEK2 pseudogene |
| NEK2P | 19169042 | End |  |  |  |  |  |  |  |
| LOC400174 | 19206828 | Start |  |  |  |  |  |  | similar to single stranded DNA binding protein 3 |
| LOC400174 | 19208002 | End |  |  |  |  |  |  |  |
| USP10P1 | 19215649 | Start |  |  |  |  |  |  | ubiquitin specific peptidase 10 pseudogene 1 |
| USP10P1 | 19219046 | End |  |  |  |  |  |  |  |
| LOC254398 | 19226260 | Start |  |  |  |  |  |  | similar to Oligophrenin 1 |
| LOC254398 | 19230470 | End |  |  |  |  |  |  |  |
| OR11H8P | 19250735 | Start |  |  |  |  |  |  | olfactory receptor, family 11, subfamily H, member 8 pseudogene |
| OR11H8P | 19252115 | End |  |  |  |  |  |  |  |
| OR11K2P | 19271245 | Start |  |  |  |  |  |  | olfactory receptor, family 11, subfamily K, member 2 pseudogene |
| OR11K2P | 19272392 | End |  |  |  |  |  |  |  |
| rs4983173 | 19272965 |  | 934 | 510 | 36 | 1.8 | 1.5 | 4.5 |  |
|  |  |  | 955.2 | 467.6 | 57.2 |  |  |  |  |
| OR4Q3 | 19285427 | Start |  |  |  |  |  |  | olfactory receptor, family 4, subfamily Q, member 3 |
| OR4Q3 | 19286368 | End |  |  |  |  |  |  |  |
| OR4H12P | 19297909 | Start |  |  |  |  |  |  | olfactory receptor, family 4, subfamily H, member 12 pseudogene |
| OR4H12P | 19298787 | End |  |  |  |  |  |  |  |
| rs4562981 | 19309086 |  | 959 | 478 | 43 | 0.6 | 10 | 10.3 |  |
|  |  |  | 969.7 | 456.5 | 53.7 |  |  |  |  |
| OR4M1 | 19318322 | Start |  |  |  |  |  |  | olfactory receptor, family 4, subfamily M, member 1 |
| OR4M1 | 19319263 | End |  |  |  |  |  |  |  |
| OR4N1P | 19334241 | Start |  |  |  |  |  |  | olfactory receptor, family 4, subfamily N, member 1 pseudogene |
| OR4N1P | 19335389 | End |  |  |  |  |  |  |  |
| rs4412905 | 19336854 |  | 570 | 813 | 97 | 14.1 | 14.2 | 4.6 |  |
|  |  |  | 644.3 | 664.4 | 171.3 |  |  |  |  |
| rs4080788 | 19337785 |  | 1278 | 202 | 0 | 0.5 | 0.3 | 3.6 |  |
|  |  |  | 1284.9 | 188.2 | 6.9 |  |  |  |  |
| rs1780870 | 19362325 |  | 1040 | 403 | 37 | 0.1 | 3.2 | 3.4 |  |
|  |  |  | 1041.4 | 400.1 | 38.4 |  |  |  |  |
| rs10130102 | 19362693 |  | 717 | 716 | 47 | 10.9 | 9.6 | 20.7 |  |
|  |  |  | 780.8 | 588.3 | 110.8 |  |  |  |  |
| OR4N2 | 19365448 | Start |  |  |  |  |  |  | olfactory receptor, family 4, subfamily N, member 2 |
| OR4N2 | 19366371 | End |  |  |  |  |  |  |  |
| rs4473104 | 19380763 |  | 1011 | 455 | 14 | 2.5 | 28.7 | 19 |  |
|  |  |  | 1036.4 | 404.2 | 39.4 |  |  |  |  |
| rs2318527 | 19381928 |  | 317 | 1020 | 143 | 50.7 | 35.3 | 25.4 |  |
|  |  |  | 462.1 | 729.8 | 288.1 |  |  |  |  |
| OR4K6P | 19385266 | Start |  |  |  |  |  |  | olfactory receptor, family 4, subfamily K, member 6 pseudogene |
| OR4K6P | 19386328 | End |  |  |  |  |  |  |  |
| rs1686549 | 19402695 |  | 748 | 711 | 21 | 14.9 | 22.4 | 23 |  |
|  |  |  | 822.8 | 561.4 | 95.8 |  |  |  |  |
| OR4K3P | 19405996 | Start |  |  |  |  |  |  | olfactory receptor, family 4, subfamily K, member 3 pseudogene |
| rs1780941 | 19406967 |  | 446 | 918 | 116 | 28.3 | 26.8 | 18.8 |  |
|  |  |  | 553.4 | 703.2 | 223.4 |  |  |  |  |
| OR4K3P | 19407342 | End |  |  |  |  |  |  |  |
| rs1686539 | 19411485 |  | 538 | 867 | 75 | 24.7 | 20.3 | 19.9 |  |
|  |  |  | 637.7 | 667.6 | 174.7 |  |  |  |  |
| OR4K2 | 19414267 | Start |  |  |  |  |  |  | olfactory receptor, family 4, subfamily K, member 2 |
| OR4K2 | 19415211 | End |  |  |  |  |  |  |  |
| rs1780934 | 19416412 |  | 696 | 751 | 33 | 16.6 | 16.3 | 16.2 |  |
|  |  |  | 775.8 | 591.5 | 112.8 |  |  |  |  |
| rs1319956 | 19417051 |  | 689 | 754 | 37 | 16.2 | 16.5 | 12 |  |
|  |  |  | 767.8 | 596.4 | 115.8 |  |  |  |  |
| rs1319954 | 19417404 |  | 693 | 754 | 33 | 16.9 | 12.3 | 18.9 |  |
|  |  |  | 773.6 | 592.8 | 113.6 |  |  |  |  |
| rs1780930 | 19417621 |  | 830 | 630 | 20 | 8.9 | 16.6 | 16.7 |  |
|  |  |  | 885.8 | 518.3 | 75.8 |  |  |  |  |
| rs8013148 | 19420550 |  | 272 | 958 | 250 | 29.1 | 28.4 | 26 |  |
|  |  |  | 381.1 | 739.8 | 359.1 |  |  |  |  |
| rs1780927 | 19424016 |  | 599 | 843 | 38 | 27.4 | 24.9 | 23.6 |  |
|  |  |  | 703.7 | 633.7 | 142.7 |  |  |  |  |
| OR4K4P | 19443611 | Start |  |  |  |  |  |  | olfactory receptor, family 4, subfamily K, member 4 pseudogene |
| rs1780909 | 19444485 |  | 673 | 786 | 21 | 23 | 21.7 | 11.5 |  |
|  |  |  | 767.8 | 596.4 | 115.8 |  |  |  |  |
| OR4K4P | 19444743 | End |  |  |  |  |  |  |  |
| rs2318498 | 19447566 |  | 1426 | 54 | 0 | 0.1 | 1.6 | 14.2 |  |
|  |  |  | 1426.5 | 53 | 0.5 |  |  |  |  |
| rs1632089 | 19453422 |  | 1162 | 318 | 0 | 1.6 | 15.7 | 5 |  |
|  |  |  | 1179.1 | 283.8 | 17.1 |  |  |  |  |
| OR4K5 | 19458606 | Start |  |  |  |  |  |  | olfactory receptor, family 4, subfamily K, member 5 |
| OR4K5 | 19459577 | End |  |  |  |  |  |  |  |
| rs1686588 | 19459714 |  | 259 | 974 | 247 | 33.3 | 7.4 | 6.6 |  |
|  |  |  | 376 | 740 | 364 |  |  |  |  |
| OR4K1 | 19473666 | Start |  |  |  |  |  |  | olfactory receptor, family 4, subfamily K, member 1 |
| OR4K1 | 19474601 | End |  |  |  |  |  |  |  |
| rs7146334 | 19483641 |  | 795 | 663 | 22 | 10.8 | 7.8 | 15.1 |  |
|  |  |  | 857.4 | 538.1 | 84.4 |  |  |  |  |
| rs6572904 | 19487234 |  | 801 | 633 | 46 | 5.4 | 13.7 | 8.2 |  |
|  |  |  | 843.8 | 547.4 | 88.8 |  |  |  |  |
| rs2635568 | 19489099 |  | 241 | 1001 | 238 | 41.2 | 31.4 | 0.2 |  |
|  |  |  | 371.5 | 740 | 368.5 |  |  |  |  |
| rs10151880 | 19489537 |  | 545 | 805 | 130 | 9.9 | -3.5 | 0.3 |  |
|  |  |  | 606.6 | 681.8 | 191.6 |  |  |  |  |
| rs4060079 | 19489770 |  | 694 | 483 | 303 | -26 | 0.4 | 0.3 |  |
|  |  |  | 591.3 | 688.4 | 200.3 |  |  |  |  |
| rs2635576 | 19489991 |  | 242 | 1005 | 233 | 42.5 | 30.1 | 6.9 |  |
|  |  |  | 374.5 | 740 | 365.5 |  |  |  |  |
| rs3916621 | 19492423 |  | 449 | 888 | 143 | 20 | 8.2 | 8.2 |  |
|  |  |  | 538.8 | 708.4 | 232.8 |  |  |  |  |
| OR4K16P | 19494446 | Start |  |  |  |  |  |  | olfactory receptor, family 4, subfamily K, member 16 pseudogene |
| OR4K16P | 19495653 | End |  |  |  |  |  |  |  |
| rs10141075 | 19495751 |  | 548 | 690 | 242 | -0.5 | -0.5 | -0.5 |  |
|  |  |  | 538.8 | 708.4 | 232.8 |  |  |  |  |
| rs11850906 | 19496544 |  | 549 | 690 | 241 | -0.5 | -0.5 | -0.4 |  |
|  |  |  | 540 | 708 | 232 |  |  |  |  |
| rs7159122 | 19502641 |  | 548 | 690 | 242 | -0.5 | -0.4 | -0.4 |  |
|  |  |  | 538.8 | 708.4 | 232.8 |  |  |  |  |
| rs7151469 | 19502828 |  | 548 | 691 | 241 | -0.4 | -0.4 | 0 |  |
|  |  |  | 539.4 | 708.2 | 232.4 |  |  |  |  |
| rs6572987 | 19502884 |  | 548 | 690 | 242 | -0.5 | 0 | 0 |  |
|  |  |  | 538.8 | 708.4 | 232.8 |  |  |  |  |
| OR4K15 | 19513518 | Start |  |  |  |  |  |  | olfactory receptor, family 4, subfamily K, member 15 |
| OR4K15 | 19514564 | End |  |  |  |  |  |  |  |
| OR4Q2P | 19540062 | Start |  |  |  |  |  |  | olfactory receptor, family 4, subfamily Q, member 2 pseudogene |
| OR4Q2P | 19541192 | End |  |  |  |  |  |  |  |
| OR4K14 | 19552260 | Start |  |  |  |  |  |  | olfactory receptor, family 4, subfamily K, member 14 |
| OR4K14 | 19553192 | End |  |  |  |  |  |  |  |
| OR4K13 | 19571843 | Start |  |  |  |  |  |  | olfactory receptor, family 4, subfamily K, member 13 |
| OR4K13 | 19572757 | End |  |  |  |  |  |  |  |
| OR4U1P | 19581815 | Start |  |  |  |  |  |  | olfactory receptor, family 4, subfamily U, member 1 pseudogene |
| OR4U1P | 19582954 | End |  |  |  |  |  |  |  |
| OR4L1 | 19598044 | Start |  |  |  |  |  |  | olfactory receptor, family 4, subfamily L, member 1 |
| OR4L1 | 19598982 | End |  |  |  |  |  |  |  |
| OR4T1P | 19631254 | Start |  |  |  |  |  |  | olfactory receptor, family 4, subfamily T, member 1 pseudogene |
| OR4T1P | 19632374 | End |  |  |  |  |  |  |  |
| OR4K17 | 19655406 | Start |  |  |  |  |  |  | olfactory receptor, family 4, subfamily K, member 17 |
| OR4K17 | 19656437 | End |  |  |  |  |  |  |  |
| OR4N5 | 19681735 | Start |  |  |  |  |  |  | olfactory receptor, family 4, subfamily N, member 5 |
| OR4N5 | 19682661 | End |  |  |  |  |  |  |  |
