# Supplementary material for: Markers typed in genome-wide analysis identify regions showing deviation from Hardy-Weinberg equilibrium
Source: BMC Res Notes. 2009 Mar 2;2:29. doi: 10.1186/1756-0500-2-29 (PMC2667528; doi:10.1186/1756-0500-2-29)
Supplement: Additional file 2 — HWETable6.doc. Table 6. Region of 15q11-14 with summed heterozygosity score exceeding 50. [file 1756-0500-2-29-S2.doc]

**Markers typed in genome-wide analysis identify regions showing deviation from Hardy-Weinberg equilibrium.**

AE Vine, D Curtis

**Additional file**

**Table 6.** Region of 15q11-14 with summed heterozygosity score exceeding 50.

| **Marker or gene** | **Position** |  | **Marker genotype counts**  **Observed**  **Expected** | | | **Heterozygosity scores for single, two and three marker analysis** | | | **Gene description** |
| --- | --- | --- | --- | --- | --- | --- | --- | --- | --- |
|  |  |  | **AA** | **AB** | **BB** | **1** | **2** | **3** |  |
| LOC646139 | 18970614 | Start |  |  |  |  |  |  | similar to hect domain and RLD 2 |
| LOC646139 | 18995069 | End |  |  |  |  |  |  |  |
| LOC100132727 | 18998028 | Start |  |  |  |  |  |  | hypothetical protein LOC100132727 |
| LOC100132727 | 18998856 | End |  |  |  |  |  |  |  |
| LOC727832 | 18999026 | Start |  |  |  |  |  |  | similar to cis-Golgi matrix protein GM130 |
| LOC727832 | 19007128 | End |  |  |  |  |  |  |  |
| LOC729786 | 19027714 | Start |  |  |  |  |  |  | similar to golgi autoantigen, golgin subfamily a, 8A |
| LOC729786 | 19041108 | End |  |  |  |  |  |  |  |
| LOC646177 | 19058780 | Start |  |  |  |  |  |  | similar to chromosome 9 open reading frame 79 |
| LOC646177 | 19064543 | End |  |  |  |  |  |  |  |
| LOC401805 | 19092050 | Start |  |  |  |  |  |  | hypothetical gene supported by NM_144726 |
| LOC401805 | 19093967 | End |  |  |  |  |  |  |  |
| BCL8 | 19134811 | Start |  |  |  |  |  |  | B-cell CLL/lymphoma 8 |
| rs6600090 | 19178339 |  | 1397 | 82 | 1 | 0 | 0 | 0.6 |  |
|  |  |  | 1397.2 | 81.6 | 1.2 |  |  |  |  |
| LOC646214 | 19187488 | Start |  |  |  |  |  |  | similar to p21-activated kinase 2 |
| LOC646214 | 19194116 | End |  |  |  |  |  |  |  |
| rs4402527 | 19207088 |  | 1480 | 0 | 0 | 0 | 0.8 | -0.2 |  |
|  |  |  | 1480 | 0 | 0 |  |  |  |  |
| BCL8 | 19221496 | End |  |  |  |  |  |  |  |
| LOC100133065 | 19238458 | Start |  |  |  |  |  |  | similar to hCG1734082 |
| LOC100133065 | 19240245 | End |  |  |  |  |  |  |  |
| DKFZP547L112 | 19250616 | Start |  |  |  |  |  |  | hypothetical protein DKFZp547L112 |
| DKFZP547L112 | 19266096 | End |  |  |  |  |  |  |  |
| LOC646243 | 19268721 | Start |  |  |  |  |  |  | similar to coxsackie virus and adenovirus receptor precursor |
| LOC646243 | 19280268 | End |  |  |  |  |  |  |  |
| A26B1 | 19305253 | Start |  |  |  |  |  |  | ANKRD26-like family B, member 1 |
| A26B1 | 19336667 | End |  |  |  |  |  |  |  |
| rs3848222 | 19356830 |  | 1244 | 236 | 0 | 0.8 | -0.2 | 0 |  |
|  |  |  | 1253.4 | 217.2 | 9.4 |  |  |  |  |
| rs2243568 | 19369108 |  | 901 | 496 | 83 | -0.5 | -0.2 | 2.9 |  |
|  |  |  | 892 | 513.9 | 74 |  |  |  |  |
| LOC440225 | 19370631 | Start |  |  |  |  |  |  | similar to Neurofibromin (Neurofibromatosis-related protein NF-1) |
| rs2251057 | 19385558 |  | 1197 | 273 | 10 | 0.3 | 6.8 | 6.9 |  |
|  |  |  | 1201.5 | 264 | 14.5 |  |  |  |  |
| rs7166137 | 19403945 |  | 832 | 635 | 13 | 10.5 | 10.6 | 17.1 |  |
|  |  |  | 892.8 | 513.4 | 73.8 |  |  |  |  |
| LOC440225 | 19405675 | End |  |  |  |  |  |  |  |
| OR11J2P | 19430549 | Start |  |  |  |  |  |  | olfactory receptor, family 11, subfamily J, member 2 pseudogene |
| OR11J2P | 19431694 | End |  |  |  |  |  |  |  |
| OR11J5P | 19439138 | Start |  |  |  |  |  |  | olfactory receptor, family 11, subfamily J, member 5 pseudogene |
| OR11J5P | 19440297 | End |  |  |  |  |  |  |  |
| LOC727899 | 19449018 | Start |  |  |  |  |  |  | hypothetical protein LOC727899 |
| LOC727899 | 19461476 | End |  |  |  |  |  |  |  |
| rs17134298 | 19464910 |  | 1478 | 2 | 0 | 0 | 24.2 | 43.8 |  |
|  |  |  | 1478 | 2 | 0 |  |  |  |  |
| LOC283804 | 19522590 | Start |  |  |  |  |  |  | similar to a disintegrin and metallopeptidase domain 6 |
| LOC283804 | 19525134 | End |  |  |  |  |  |  |  |
| LOC100133063 | 19542669 | Start |  |  |  |  |  |  | similar to breast cancer anti-estrogen resistance 1 |
| LOC100133063 | 19551572 | End |  |  |  |  |  |  |  |
| LOC100132026 | 19598556 | Start |  |  |  |  |  |  | hypothetical protein LOC100132026 |
| LOC100132026 | 19599445 | End |  |  |  |  |  |  |  |
| LOC100132293 | 19617008 | Start |  |  |  |  |  |  | hypothetical LOC100132293 |
| LOC100132293 | 19619558 | End |  |  |  |  |  |  |  |
| LOC400968 | 19620671 | Start |  |  |  |  |  |  | hypothetical LOC400968 |
| LOC400968 | 19621307 | End |  |  |  |  |  |  |  |
| LOC727914 | 19624879 | Start |  |  |  |  |  |  | similar to Ribosome biogenesis protein BMS1 homolog |
| LOC727914 | 19635323 | End |  |  |  |  |  |  |  |
| OR11J1P | 19769501 | Start |  |  |  |  |  |  | olfactory receptor, family 11, subfamily J, member 1 pseudogene |
| OR11J1P | 19770660 | End |  |  |  |  |  |  |  |
| LOC727924 | 19779378 | Start |  |  |  |  |  |  | hypothetical protein LOC727924 |
| LOC727924 | 19791849 | End |  |  |  |  |  |  |  |
| OR11H3P | 19798765 | Start |  |  |  |  |  |  | olfactory receptor, family 11, subfamily H, member 3 pseudogene |
| OR11H3P | 19799911 | End |  |  |  |  |  |  |  |
| OR11K1P | 19820118 | Start |  |  |  |  |  |  | olfactory receptor, family 11, subfamily K, member 1 pseudogene |
| OR11K1P | 19821246 | End |  |  |  |  |  |  |  |
| rs1346662 | 19821421 |  | 684 | 782 | 14 | 24.1 | 43.4 | 38 |  |
|  |  |  | 780.8 | 588.3 | 110.8 |  |  |  |  |
| rs2880332 | 19830049 |  | 616 | 839 | 25 | 29.5 | 33.2 | 38.4 |  |
|  |  |  | 724.5 | 622 | 133.5 |  |  |  |  |
| rs7166438 | 19831773 |  | 751 | 716 | 13 | 17.1 | 21.5 | 46.8 |  |
|  |  |  | 831 | 556 | 93 |  |  |  |  |
| rs7166613 | 19831810 |  | 673 | 797 | 10 | 27 | 52.9 | 32.2 |  |
|  |  |  | 775.8 | 591.5 | 112.8 |  |  |  |  |
| OR4Q1P | 19833632 | Start |  |  |  |  |  |  | olfactory receptor, family 4, subfamily Q, member 1 pseudogene |
| OR4Q1P | 19834812 | End |  |  |  |  |  |  |  |
| rs4238548 | 19835428 |  | 577 | 884 | 19 | 38.4 | 24.3 | 22.8 |  |
|  |  |  | 701.6 | 634.8 | 143.6 |  |  |  |  |
| LOC652851 | 19845949 | Start |  |  |  |  |  |  | similar to Olfactory receptor 4H12 |
| OR4H6P | 19846219 | Start |  |  |  |  |  |  | olfactory receptor, family 4, subfamily H, member 6 pseudogene |
| OR4H6P | 19846855 | End |  |  |  |  |  |  |  |
| LOC652851 | 19846957 | End |  |  |  |  |  |  |  |
| rs4310812 | 19852603 |  | 1177 | 301 | 2 | 1.2 | 2.1 | 2.3 |  |
|  |  |  | 1190.7 | 273.6 | 15.7 |  |  |  |  |
| OR4M2 | 19869940 | Start |  |  |  |  |  |  | olfactory receptor, family 4, subfamily M, member 2 |
| OR4M2 | 19870881 | End |  |  |  |  |  |  |  |
| rs2082048 | 19876834 |  | 777 | 630 | 73 | 2.7 | 2.8 | 33.3 |  |
|  |  |  | 805.7 | 572.6 | 101.7 |  |  |  |  |
| OR4N4 | 19883837 | Start |  |  |  |  |  |  | olfactory receptor, family 4, subfamily N, member 4 |
| OR4N4 | 19884787 | End |  |  |  |  |  |  |  |
| rs1896867 | 19886462 |  | 1281 | 199 | 0 | 0.5 | 39.3 | 34.9 |  |
|  |  |  | 1287.7 | 185.6 | 6.7 |  |  |  |  |
| rs4779295 | 19911648 |  | 515 | 945 | 20 | 50.5 | 38.3 | 31.9 |  |
|  |  |  | 658.9 | 657.2 | 163.9 |  |  |  |  |
| rs11854399 | 19912458 |  | 845 | 622 | 13 | 9.7 | 28.8 | 56 |  |
|  |  |  | 902.9 | 506.1 | 70.9 |  |  |  |  |
| OR4N3P | 19914826 | Start |  |  |  |  |  |  | olfactory receptor, family 4, subfamily N, member 3 pseudogene |
| OR4N3P | 19915749 | End |  |  |  |  |  |  |  |
| rs1835208 | 19915944 |  | 601 | 856 | 23 | 32.7 | 80.2 | 48.7 |  |
|  |  |  | 715.4 | 627.1 | 137.4 |  |  |  |  |
| rs7161939 | 19922588 |  | 169 | 1159 | 152 | 104.5 | 58 | 48.7 |  |
|  |  |  | 378.5 | 739.9 | 361.5 |  |  |  |  |
| LOC388076 | 19941517 | Start |  |  |  |  |  |  | similar to ribosomal protein S8 |
| LOC388076 | 19942223 | End |  |  |  |  |  |  |  |
| rs10519379 | 19943075 |  | 1233 | 191 | 56 | -7.3 | 7.2 | 7.1 |  |
|  |  |  | 1192.5 | 272 | 15.5 |  |  |  |  |
| rs11635275 | 19943185 |  | 689 | 774 | 17 | 22.4 | 22.4 | 19.4 |  |
|  |  |  | 782.3 | 587.4 | 110.3 |  |  |  |  |
| rs8035298 | 19945295 |  | 1479 | 1 | 0 | 0 | 0.1 | 75.3 |  |
|  |  |  | 1479 | 1 | 0 |  |  |  |  |
| IGHV1OR15-1 | 19949604 | Start |  |  |  |  |  |  | immunoglobulin heavy variable 1/OR15-1 |
| rs2271569 | 19950116 |  | 1422 | 58 | 0 | 0.1 | 75.1 | 45.3 |  |
|  |  |  | 1422.6 | 56.9 | 0.6 |  |  |  |  |
| IGHV1OR15-1 | 19950239 | End |  |  |  |  |  |  |  |
| LOC646370 | 19967170 | Start |  |  |  |  |  |  | similar to Ig heavy chain V-I region V35 precursor |
| LOC646370 | 19967857 | End |  |  |  |  |  |  |  |
| rs1813939 | 19971289 |  | 333 | 1077 | 70 | 77.7 | 45.7 | 54.8 |  |
|  |  |  | 513.2 | 716.6 | 250.2 |  |  |  |  |
| LOC642131 | 19972818 | Start |  |  |  |  |  |  | similar to Ig heavy chain V-II region ARH-77 precursor |
| LOC642131 | 19974738 | End |  |  |  |  |  |  |  |
| LOC646379 | 19983953 | Start |  |  |  |  |  |  | similar to Ig heavy chain V-I region V35 precursor |
| rs2055220 | 19985717 |  | 1115 | 356 | 9 | 1.2 | 24 | 17.6 |  |
|  |  |  | 1129.6 | 326.7 | 23.6 |  |  |  |  |
| rs11633173 | 19988041 |  | 623 | 838 | 19 | 30.7 | 20.5 | 44.9 |  |
|  |  |  | 733.6 | 616.8 | 129.6 |  |  |  |  |
| rs1826882 | 19989036 |  | 894 | 532 | 54 | 1 | 39.9 | 25.5 |  |
|  |  |  | 909.2 | 501.6 | 69.2 |  |  |  |  |
| LOC646379 | 19991387 | End |  |  |  |  |  |  |  |
| rs1001444 | 20016405 |  | 325 | 1084 | 71 | 80 | 45.8 | 21.8 |  |
|  |  |  | 507.9 | 718.2 | 253.9 |  |  |  |  |
| LOC646396 | 20046847 | Start |  |  |  |  |  |  | similar to Zinc finger CCHC domain-containing protein 2 |
| rs1985933 | 20053967 |  | 1059 | 387 | 34 | 0 | 2.3 | 3.3 |  |
|  |  |  | 1060 | 385.1 | 35 |  |  |  |  |
| rs7166350 | 20059872 |  | 732 | 672 | 76 | 4.4 | 5.1 | 3 |  |
|  |  |  | 770.7 | 594.6 | 114.7 |  |  |  |  |
| LOC646396 | 20072491 | End |  |  |  |  |  |  |  |
| rs11259883 | 20079140 |  | 919 | 536 | 25 | 3.6 | 2 | 1.2 |  |
|  |  |  | 952 | 470 | 58 |  |  |  |  |
| LOC100130556 | 20241196 | Start |  |  |  |  |  |  | hypothetical LOC100130556 |
| LOC100130556 | 20242310 | End |  |  |  |  |  |  |  |
| ABCB10P | 20243548 | Start |  |  |  |  |  |  | ATP-binding cassette, sub-family B (MDR/TAP), member 10 pseudogene |
| ABCB10P | 20244000 | End |  |  |  |  |  |  |  |
| LOC100132979 | 20253649 | Start |  |  |  |  |  |  | similar to FLJ32679 protein |
| LOC100132979 | 20267066 | End |  |  |  |  |  |  |  |
| LOC283767 | 20287610 | Start |  |  |  |  |  |  | FLJ40198 protein |
| LOC100131452 | 20295617 | Start |  |  |  |  |  |  | hypothetical protein LOC100131452 |
| LOC283767 | 20296164 | End |  |  |  |  |  |  |  |
| LOC100131452 | 20296446 | End |  |  |  |  |  |  |  |
| LOC100129539 | 20299406 | Start |  |  |  |  |  |  | similar to FLJ00287 protein |
| LOC100129539 | 20300689 | End |  |  |  |  |  |  |  |
| LOC729894 | 20328861 | Start |  |  |  |  |  |  | similar to Engulfment and cell motility protein 2 (CED-12 homolog A) |
| rs4405519 | 20329239 |  | 517 | 733 | 230 | 0.6 | 0.1 | 0 |  |
|  |  |  | 527.4 | 712.2 | 240.4 |  |  |  |  |
| rs12900257 | 20335459 |  | 783 | 585 | 112 | -0.1 | 0 | 0 |  |
|  |  |  | 781.6 | 587.9 | 110.6 |  |  |  |  |
| LOC729894 | 20341338 | End |  |  |  |  |  |  |  |
| TUBGCP5 | 20384836 | Start |  |  |  |  |  |  | tubulin, gamma complex associated protein 5 |
| TUBGCP5 | 20425332 | End |  |  |  |  |  |  |  |
| CYFIP1 | 20444125 | Start |  |  |  |  |  |  | cytoplasmic FMR1 interacting protein 1 |
| CYFIP1 | 20555044 | End |  |  |  |  |  |  |  |
