# Supplementary material for: Markers typed in genome-wide analysis identify regions showing deviation from Hardy-Weinberg equilibrium
Source: BMC Res Notes. 2009 Mar 2;2:29. doi: 10.1186/1756-0500-2-29 (PMC2667528; doi:10.1186/1756-0500-2-29)
Supplement: Additional file 3 — HWETable7.doc. Table 7. Regions with absolute summed heterozygosity score exceeding 15. [file 1756-0500-2-29-S3.doc]

**Markers typed in genome-wide analysis identify regions showing deviation from Hardy-Weinberg equilibrium.**

AE Vine, D Curtis

**Additional file**

**Table 7.** Regions with absolute summed heterozygosity score exceeding 15.

The table shows markers and genes in regions showing deviation from HWE using a threshold for the summed heterozygosity scores (ignoring the highest-scoring marker) with an absolute value exceeding 15. Observed counts are shown for each marker genotype with the expected counts in the row below. Heterozygosity scores, defined as –log(p) for increased heterozygosity and log(p) for increased homozygosity, are shown for individual markers and for two and three marker haplotypes.

| **Cytogenetic location** | **Marker or gene** | **Position** |  | **Marker genotype counts**  **Observed**  **Expected** | | | **Heterozygosity scores for single, two and three marker analysis** | | | **Gene description** |
| --- | --- | --- | --- | --- | --- | --- | --- | --- | --- | --- |
|  |  |  |  | **AA** | **AB** | **BB** | **1** | **2** | **3** |  |
| 1p34.1 | GPBP1L1 | 45865567 | Start |  |  |  |  |  |  | GC-rich promoter binding protein 1-like 1 |
|  | GPBP1L1 | 45899398 | End |  |  |  |  |  |  |  |
| 1p34.1 | TMEM69 | 45926434 | Start |  |  |  |  |  |  | transmembrane protein 69 |
|  | TMEM69 | 45932695 | End |  |  |  |  |  |  |  |
| 1p34-p32 | IPP | 45936977 | Start |  |  |  |  |  |  | intracisternal A particle-promoted polypeptide |
|  | IPP | 45984720 | End |  |  |  |  |  |  |  |
| 1p34.1 | LOC440587 | 46006551 | Start |  |  |  |  |  |  | similar to 60S ribosomal protein L6 (TAX-responsive enhancer element-binding protein 107) (TAXREB107) (Neoplasm-related protein C140) |
|  | LOC440587 | 46024119 | End |  |  |  |  |  |  |  |
| 1p34.1 | MAST2 | 46041872 | Start |  |  |  |  |  |  | microtubule associated serine/threonine kinase 2 |
|  | rs10890365 | 46071368 |  | 711 | 665 | 104 | 2 | 2.6 | 2.5 |  |
|  |  |  |  | 735.7 | 615.5 | 128.7 |  |  |  |  |
|  | rs4545281 | 46072074 |  | 428 | 785 | 267 | 2.3 | 2.2 | 2.8 |  |
|  |  |  |  | 454.9 | 731.2 | 293.9 |  |  |  |  |
|  | rs7556436 | 46075900 |  | 430 | 782 | 268 | 2.1 | 2.7 | 2.6 |  |
|  |  |  |  | 455.4 | 731.1 | 293.4 |  |  |  |  |
| 1p34.1 | LOC100101407 | 46083602 | Start |  |  |  |  |  |  | FLJ11184 pseudogene |
|  | LOC100101407 | 46085968 | End |  |  |  |  |  |  |  |
|  | rs6689417 | 46100028 |  | 804 | 584 | 92 | 0.4 | 2.9 | 2.9 |  |
|  |  |  |  | 811.6 | 568.7 | 99.6 |  |  |  |  |
|  | rs10890370 | 46101237 |  | 710 | 666 | 104 | 2.1 | 2.1 | 2.1 |  |
|  |  |  |  | 735 | 615.9 | 129 |  |  |  |  |
|  | rs10890373 | 46105775 |  | 710 | 666 | 104 | 2.1 | 2.1 | 2.4 |  |
|  |  |  |  | 735 | 615.9 | 129 |  |  |  |  |
|  | rs10789486 | 46120687 |  | 710 | 666 | 104 | 2.1 | 2.4 | 2.4 |  |
|  |  |  |  | 735 | 615.9 | 129 |  |  |  |  |
|  | rs6700322 | 46121196 |  | 814 | 572 | 94 | 0.2 | 2.4 | 2.4 |  |
|  |  |  |  | 817.6 | 564.9 | 97.6 |  |  |  |  |
|  | rs10890378 | 46122677 |  | 711 | 665 | 104 | 2 | 2.1 | 2 |  |
|  |  |  |  | 735.7 | 615.5 | 128.7 |  |  |  |  |
|  | rs11211232 | 46123166 |  | 710 | 666 | 104 | 2.1 | 2 | 2 |  |
|  |  |  |  | 735 | 615.9 | 129 |  |  |  |  |
|  | rs4660905 | 46173607 |  | 709 | 666 | 105 | 2 | 2 | 2.5 |  |
|  |  |  |  | 733.6 | 616.8 | 129.6 |  |  |  |  |
|  | rs4660334 | 46174146 |  | 710 | 666 | 104 | 2.1 | 2.5 | 2.5 |  |
|  |  |  |  | 735 | 615.9 | 129 |  |  |  |  |
|  | rs4073846 | 46174901 |  | 428 | 783 | 269 | 2.1 | 2.5 | 2.4 |  |
|  |  |  |  | 453.8 | 731.5 | 294.8 |  |  |  |  |
|  | rs4073847 | 46175032 |  | 710 | 665 | 105 | 2 | 2.5 | 3 |  |
|  |  |  |  | 734.3 | 616.3 | 129.3 |  |  |  |  |
|  | rs11579634 | 46178411 |  | 423 | 789 | 268 | 2.5 | 2.9 | 3.2 |  |
|  |  |  |  | 451.6 | 731.9 | 296.6 |  |  |  |  |
|  | rs11589562 | 46178741 |  | 432 | 795 | 253 | 3.2 | 3.5 | 3.2 |  |
|  |  |  |  | 464.9 | 729.2 | 285.9 |  |  |  |  |
|  | rs6677777 | 46187856 |  | 708 | 668 | 104 | 2.2 | 2.5 | 2.5 |  |
|  |  |  |  | 733.6 | 616.8 | 129.6 |  |  |  |  |
|  | rs11211247 | 46188607 |  | 813 | 573 | 94 | 0.2 | 2.4 | 2.3 |  |
|  |  |  |  | 816.8 | 565.4 | 97.8 |  |  |  |  |
|  | rs946527 | 46197990 |  | 725 | 656 | 99 | 2 | 1.9 | 1.9 |  |
|  |  |  |  | 749.2 | 607.6 | 123.2 |  |  |  |  |
|  | rs946526 | 46199188 |  | 1348 | 130 | 2 | 0.1 | 1.9 | 0 |  |
|  |  |  |  | 1349 | 127.9 | 3 |  |  |  |  |
|  | rs785480 | 46204184 |  | 711 | 664 | 105 | 1.9 | 0 | 0 |  |
|  |  |  |  | 735 | 615.9 | 129 |  |  |  |  |
|  | MAST2 | 46274383 | End |  |  |  |  |  |  |  |
| 1p34.1 | PIK3R3 | 46278399 | Start |  |  |  |  |  |  | phosphoinositide-3-kinase, regulatory subunit 3 (p55, gamma) |
| 1p34.1 | LOC100133124 | 46291355 | Start |  |  |  |  |  |  | similar to hCG2041843 |
|  | PIK3R3 | 46370901 | End |  |  |  |  |  |  |  |
|  | LOC100133124 | 46377340 | End |  |  |  |  |  |  |  |
| 1q32.1 | CAMSAP1L1 | 198975309 | Start |  |  |  |  |  |  | calmodulin regulated spectrin-associated protein 1-like 1 |
|  | CAMSAP1L1 | 199096455 | End |  |  |  |  |  |  |  |
| 1q32.1 | GPR25 | 199108789 | Start |  |  |  |  |  |  | G protein-coupled receptor 25 |
|  | GPR25 | 199109874 | End |  |  |  |  |  |  |  |
| 1q32.1 | C1orf106 | 199127292 | Start |  |  |  |  |  |  | chromosome 1 open reading frame 106 |
|  | rs2843415 | 199127420 |  | 1467 | 13 | 0 | 0 | -0.1 | 0 |  |
|  |  |  |  | 1467 | 12.9 | 0 |  |  |  |  |
|  | rs2741853 | 199137328 |  | 638 | 665 | 177 | -0.1 | 0 | -0.4 |  |
|  |  |  |  | 636.4 | 668.2 | 175.4 |  |  |  |  |
|  | rs1358451 | 199138365 |  | 1458 | 22 | 0 | 0 | -3.4 | -3.4 |  |
|  |  |  |  | 1458.1 | 21.8 | 0.1 |  |  |  |  |
|  | rs2792810 | 199146603 |  | 1468 | 0 | 12 | -6.1 | -6.1 | -0.9 |  |
|  |  |  |  | 1456.1 | 23.8 | 0.1 |  |  |  |  |
|  | rs3767424 | 199148779 |  | 1480 | 0 | 0 | 0 | -0.1 | 13.4 |  |
|  |  |  |  | 1480 | 0 | 0 |  |  |  |  |
|  | C1orf106 | 199151486 | End |  |  |  |  |  |  |  |
| 1q32.1 | C1orf81 | 199151855 | Start |  |  |  |  |  |  | chromosome 1 open reading frame 81 |
|  | rs805909 | 199160517 |  | 1143 | 312 | 25 | -0.1 | 13.4 | 3.9 |  |
|  |  |  |  | 1140.1 | 317.7 | 22.1 |  |  |  |  |
|  | rs1819043 | 199163659 |  | 345 | 940 | 195 | 26.4 | 9 | 8.8 |  |
|  |  |  |  | 448.8 | 732.4 | 298.8 |  |  |  |  |
|  | rs3767421 | 199163817 |  | 382 | 753 | 345 | 0.3 | 0.3 | 8.7 |  |
|  |  |  |  | 388.7 | 739.5 | 351.7 |  |  |  |  |
|  | rs805911 | 199172076 |  | 1463 | 17 | 0 | 0 | 24.7 | 8.8 |  |
|  |  |  |  | 1463 | 16.9 | 0 |  |  |  |  |
|  | rs705736 | 199173739 |  | 361 | 933 | 186 | 25.4 | 9 | 8.2 |  |
|  |  |  |  | 462.7 | 729.7 | 287.7 |  |  |  |  |
|  | C1orf81 | 199202415 | End |  |  |  |  |  |  |  |
| 1pter-q31.3 | KIF21B | 199205143 | Start |  |  |  |  |  |  | kinesin family member 21B |
|  | rs7522991 | 199223808 |  | 383 | 754 | 343 | 0.3 | 0.2 | 8.3 |  |
|  |  |  |  | 390.3 | 739.5 | 350.3 |  |  |  |  |
|  | rs6696611 | 199225346 |  | 395 | 742 | 343 | 0.1 | 8 | 7.9 |  |
|  |  |  |  | 396.5 | 739.1 | 344.5 |  |  |  |  |
|  | rs705747 | 199236940 |  | 366 | 936 | 178 | 26.5 | 26 | 8.7 |  |
|  |  |  |  | 470 | 728.1 | 282 |  |  |  |  |
|  | rs697455 | 199243733 |  | 1468 | 12 | 0 | 0 | 0.3 | 0.3 |  |
|  |  |  |  | 1468 | 12 | 0 |  |  |  |  |
|  | rs3767406 | 199243903 |  | 383 | 754 | 343 | 0.3 | 0.3 | 0.2 |  |
|  |  |  |  | 390.3 | 739.5 | 350.3 |  |  |  |  |
|  | rs497824 | 199256967 |  | 1468 | 12 | 0 | 0 | 0.1 | 0.2 |  |
|  |  |  |  | 1468 | 12 | 0 |  |  |  |  |
|  | KIF21B | 199259451 | End |  |  |  |  |  |  |  |
|  | rs705750 | 199263303 |  | 632 | 673 | 175 | 0.1 | 0.2 | 0.3 |  |
|  |  |  |  | 633.8 | 669.4 | 176.8 |  |  |  |  |
|  | rs2249811 | 199263398 |  | 383 | 754 | 343 | 0.3 | 0.3 | 0 |  |
|  |  |  |  | 390.3 | 739.5 | 350.3 |  |  |  |  |
| 1q32 | CACNA1S | 199275263 | Start |  |  |  |  |  |  | calcium channel, voltage-dependent, L type, alpha 1S subunit |
|  | rs3767392 | 199278758 |  | 364 | 756 | 360 | 0.4 | 0 | 0 |  |
|  |  |  |  | 372 | 740 | 368 |  |  |  |  |
|  | CACNA1S | 199348317 | End |  |  |  |  |  |  |  |
| 1q32.1 | ASCL5 | 199350256 | Start |  |  |  |  |  |  | achaete-scute complex homolog 5 (Drosophila) |
|  | ASCL5 | 199364960 | End |  |  |  |  |  |  |  |
| 1q41 | TMEM9 | 199370523 | Start |  |  |  |  |  |  | transmembrane protein 9 |
|  | TMEM9 | 199390243 | End |  |  |  |  |  |  |  |
| 1q32.1 | LOC440706 | 199427630 | Start |  |  |  |  |  |  | similar to titin isoform N2-A |
| 1q32.1 | DKFZp434B1231 | 199448393 | Start |  |  |  |  |  |  | eEF1A2 binding protein |
|  | LOC440706 | 199448868 | End |  |  |  |  |  |  |  |
|  | DKFZp434B1231 | 199464697 | End |  |  |  |  |  |  |  |
|  | rs1492691 | 220562574 |  | 358 | 796 | 326 | 2.5 | 1.5 | 1.9 |  |
|  |  |  |  | 386.2 | 739.7 | 354.2 |  |  |  |  |
|  | rs10916248 | 220563083 |  | 974 | 456 | 50 | 0.1 | 2.2 | 2 |  |
|  |  |  |  | 976.2 | 451.6 | 52.2 |  |  |  |  |
|  | rs4653993 | 220564965 |  | 339 | 810 | 331 | 3.6 | 3.2 | 3.2 |  |
|  |  |  |  | 374 | 740 | 366 |  |  |  |  |
|  | rs12402838 | 220566668 |  | 358 | 800 | 322 | 2.8 | 2.7 | 1.2 |  |
|  |  |  |  | 388.2 | 739.6 | 352.2 |  |  |  |  |
|  | rs12403374 | 220566807 |  | 357 | 797 | 326 | 2.5 | 1.2 | 1.1 |  |
|  |  |  |  | 385.7 | 739.7 | 354.7 |  |  |  |  |
|  | rs7543027 | 220567232 |  | 890 | 513 | 77 | -0.1 | 1.1 | 1 |  |
|  |  |  |  | 888.2 | 516.7 | 75.2 |  |  |  |  |
|  | rs6604887 | 220567559 |  | 358 | 796 | 326 | 2.5 | 2.4 | 2.3 |  |
|  |  |  |  | 386.2 | 739.7 | 354.2 |  |  |  |  |
|  | rs10753443 | 220570903 |  | 358 | 794 | 328 | 2.3 | 2.2 | 2.2 |  |
|  |  |  |  | 385.2 | 739.7 | 355.2 |  |  |  |  |
|  | rs10916264 | 220575173 |  | 352 | 792 | 336 | 2.2 | 2.1 | 2.1 |  |
|  |  |  |  | 378 | 739.9 | 362 |  |  |  |  |
|  | rs6692043 | 220575837 |  | 354 | 790 | 336 | 2 | 2.1 | 0.6 |  |
|  |  |  |  | 379.1 | 739.9 | 361.1 |  |  |  |  |
|  | rs4653923 | 220576221 |  | 353 | 791 | 336 | 2.1 | 0.6 | 0 |  |
|  |  |  |  | 378.5 | 739.9 | 361.5 |  |  |  |  |
|  | rs10916290 | 220591110 |  | 793 | 592 | 95 | 0.4 | 0 | 0 |  |
|  |  |  |  | 801.3 | 575.4 | 103.3 |  |  |  |  |
| 1q41 | TRNAT-UGU | 220704970 | Start |  |  |  |  |  |  | transfer RNA threonine (anticodon UGU) |
|  | TRNAT-UGU | 220705042 | End |  |  |  |  |  |  |  |
| 1q41 | LOC728615 | 220708059 | Start |  |  |  |  |  |  | similar to capicua homolog |
|  | LOC728615 | 220710316 | End |  |  |  |  |  |  |  |
| 1q41 | LOC100130483 | 220710552 | Start |  |  |  |  |  |  | hypothetical LOC100130483 |
| 1q41 | LOC100132960 | 220710629 | Start |  |  |  |  |  |  | similar to hCG1793472 |
|  | LOC100130483 | 220712444 | End |  |  |  |  |  |  |  |
|  | LOC100132960 | 220712833 | End |  |  |  |  |  |  |  |
| 1q41 | LOC653056 | 220712929 | Start |  |  |  |  |  |  | hypothetical LOC653056 |
| 1q41 | LOC728417 | 220714060 | Start |  |  |  |  |  |  | hypothetical protein LOC728417 |
|  | LOC653056 | 220716504 | End |  |  |  |  |  |  |  |
|  | LOC728417 | 220730060 | End |  |  |  |  |  |  |  |
| 1q41 | LOC728624 | 220744096 | Start |  |  |  |  |  |  | hypothetical protein LOC728624 |
|  | LOC728624 | 220759613 | End |  |  |  |  |  |  |  |
| 1q41 | KIAA1822L | 220762225 | Start |  |  |  |  |  |  | KIAA1822-like |
|  | KIAA1822L | 220788067 | End |  |  |  |  |  |  |  |
| 1q42.3 | GNG4 | 233777608 | Start |  |  |  |  |  |  | guanine nucleotide binding protein (G protein), gamma 4 |
| 1q42.3 | LOC645645 | 233818069 | Start |  |  |  |  |  |  | hypothetical protein LOC645645 |
|  | LOC645645 | 233818744 | End |  |  |  |  |  |  |  |
| 1q42.3 | LOC100131725 | 233837346 | Start |  |  |  |  |  |  | hypothetical LOC100131725 |
|  | LOC100131725 | 233846322 | End |  |  |  |  |  |  |  |
|  | GNG4 | 233880677 | End |  |  |  |  |  |  |  |
| 1q42.1-q42.2 | LYST | 233890966 | Start |  |  |  |  |  |  | lysosomal trafficking regulator |
|  | rs2927934 | 233943139 |  | 1039 | 410 | 31 | 0.4 | -0.8 | -1.2 |  |
|  |  |  |  | 1045.6 | 396.7 | 37.6 |  |  |  |  |
|  | rs4258212 | 233943440 |  | 591 | 655 | 234 | -1.5 | -2 | -1.9 |  |
|  |  |  |  | 570 | 696.9 | 213 |  |  |  |  |
|  | rs2045955 | 233946090 |  | 504 | 671 | 305 | -2.4 | -2.2 | -1.1 |  |
|  |  |  |  | 476.2 | 726.6 | 277.2 |  |  |  |  |
|  | rs11588591 | 233946995 |  | 497 | 677 | 306 | -2.1 | -1 | -1.1 |  |
|  |  |  |  | 471.7 | 727.7 | 280.7 |  |  |  |  |
|  | rs1842079 | 233951238 |  | 928 | 472 | 80 | -0.8 | -1.1 | -1.1 |  |
|  |  |  |  | 915.5 | 497.1 | 67.5 |  |  |  |  |
|  | rs1842082 | 233951526 |  | 500 | 675 | 305 | -2.2 | -2.2 | -2.3 |  |
|  |  |  |  | 473.9 | 727.2 | 278.9 |  |  |  |  |
|  | rs1478913 | 233951900 |  | 493 | 678 | 309 | -2.1 | -2.2 | -2.2 |  |
|  |  |  |  | 467.7 | 728.6 | 283.7 |  |  |  |  |
|  | rs4436381 | 233961144 |  | 491 | 674 | 315 | -2.4 | -2.3 | -2.3 |  |
|  |  |  |  | 463.2 | 729.5 | 287.2 |  |  |  |  |
|  | rs4329502 | 233961344 |  | 484 | 678 | 318 | -2.2 | -2.2 | -2.2 |  |
|  |  |  |  | 457.7 | 730.7 | 291.7 |  |  |  |  |
|  | rs4397648 | 233961362 |  | 484 | 678 | 318 | -2.2 | -2.2 | -2.2 |  |
|  |  |  |  | 457.7 | 730.7 | 291.7 |  |  |  |  |
|  | rs4329503 | 233961605 |  | 484 | 678 | 318 | -2.2 | -2.2 | -1.7 |  |
|  |  |  |  | 457.7 | 730.7 | 291.7 |  |  |  |  |
|  | rs10802613 | 233962138 |  | 485 | 677 | 318 | -2.3 | -1.7 | 0 |  |
|  |  |  |  | 458.2 | 730.6 | 291.2 |  |  |  |  |
|  | rs16835305 | 233972398 |  | 1422 | 58 | 0 | 0.1 | 0 | 0 |  |
|  |  |  |  | 1422.6 | 56.9 | 0.6 |  |  |  |  |
|  | LYST | 234096843 | End |  |  |  |  |  |  |  |
| 1q43 | RGS7 | 239005440 | Start |  |  |  |  |  |  | regulator of G-protein signaling 7 |
| 1q43 | RGS7 | 239005440 | Start |  |  |  |  |  |  | regulator of G-protein signaling 7 |
| 1q43 | LOC729138 | 239155229 | Start |  |  |  |  |  |  | 60S acidic ribosomal protein P1 pseudogene |
| 1q43 | LOC729138 | 239155229 | Start |  |  |  |  |  |  | 60S acidic ribosomal protein P1 pseudogene |
|  | LOC729138 | 239228321 | End |  |  |  |  |  |  |  |
|  | LOC729138 | 239228321 | End |  |  |  |  |  |  |  |
|  | rs10803082 | 239244800 |  | 985 | 445 | 50 | 0 | 0 | 0 |  |
|  |  |  |  | 985.2 | 444.7 | 50.2 |  |  |  |  |
|  | rs10926845 | 239245063 |  | 1480 | 0 | 0 | 0 | 0 | 0 |  |
|  |  |  |  | 1480 | 0 | 0 |  |  |  |  |
|  | rs10737884 | 239245101 |  | 1005 | 429 | 46 | 0 | 0 | -0.4 |  |
|  |  |  |  | 1004.9 | 429.3 | 45.9 |  |  |  |  |
|  | rs10803085 | 239267872 |  | 1472 | 8 | 0 | 0 | 0.1 | -0.2 |  |
|  |  |  |  | 1472 | 8 | 0 |  |  |  |  |
|  | rs9943068 | 239272210 |  | 430 | 741 | 309 | 0.1 | -0.2 | -0.2 |  |
|  |  |  |  | 433 | 735.1 | 312 |  |  |  |  |
|  | rs2809879 | 239274536 |  | 582 | 673 | 225 | -0.7 | -0.6 | -0.5 |  |
|  |  |  |  | 570 | 696.9 | 213 |  |  |  |  |
|  | rs2151552 | 239274934 |  | 1473 | 7 | 0 | 0 | -0.4 | 9.3 |  |
|  |  |  |  | 1473 | 7 | 0 |  |  |  |  |
|  | rs2780781 | 239279955 |  | 602 | 670 | 208 | -0.4 | 9.4 | 11.3 |  |
|  |  |  |  | 593.2 | 687.6 | 199.2 |  |  |  |  |
|  | rs2809868 | 239284269 |  | 310 | 963 | 207 | 31.3 | 29.7 | 18.1 |  |
|  |  |  |  | 423.3 | 736.4 | 320.3 |  |  |  |  |
|  | rs12066858 | 239300276 |  | 320 | 1017 | 143 | 49.8 | 27.5 | 27 |  |
|  |  |  |  | 463.8 | 729.4 | 286.8 |  |  |  |  |
|  | rs2210612 | 239315198 |  | 921 | 496 | 63 | 0.1 | 0.1 | -0.3 |  |
|  |  |  |  | 923.4 | 491.3 | 65.4 |  |  |  |  |
|  | rs12043626 | 239319611 |  | 1467 | 13 | 0 | 0 | -0.3 | -0.1 |  |
|  |  |  |  | 1467 | 12.9 | 0 |  |  |  |  |
|  | rs914944 | 239329563 |  | 401 | 727 | 352 | -0.3 | -0.1 | -0.1 |  |
|  |  |  |  | 394.9 | 739.2 | 345.9 |  |  |  |  |
|  | rs1333691 | 239332027 |  | 484 | 721 | 275 | -0.1 | -0.1 | -0.2 |  |
|  |  |  |  | 481.9 | 725.2 | 272.9 |  |  |  |  |
|  | rs12143316 | 239333125 |  | 400 | 728 | 352 | -0.3 | -0.3 | -0.3 |  |
|  |  |  |  | 394.4 | 739.2 | 346.4 |  |  |  |  |
|  | rs4658868 | 239345881 |  | 402 | 723 | 355 | -0.4 | -0.4 | -0.3 |  |
|  |  |  |  | 393.9 | 739.3 | 346.9 |  |  |  |  |
|  | rs4412580 | 239347041 |  | 1479 | 1 | 0 | 0 | -0.3 | 0 |  |
|  |  |  |  | 1479 | 1 | 0 |  |  |  |  |
|  | rs10926897 | 239353803 |  | 400 | 726 | 354 | -0.3 | 0 | 0 |  |
|  |  |  |  | 393.4 | 739.3 | 347.4 |  |  |  |  |
|  | rs10926902 | 239371472 |  | 1478 | 1 | 1 | -0.6 | 0.2 | 0.5 |  |
|  |  |  |  | 1477 | 3 | 0 |  |  |  |  |
|  | rs4278360 | 239379098 |  | 1088 | 368 | 24 | 0.3 | 0.5 | 0.7 |  |
|  |  |  |  | 1093.2 | 357.5 | 29.2 |  |  |  |  |
|  | rs2491826 | 239380743 |  | 750 | 619 | 111 | 0.4 | 1 | 3.3 |  |
|  |  |  |  | 758.5 | 602.1 | 119.5 |  |  |  |  |
|  | rs4658507 | 239382442 |  | 577 | 695 | 208 | 0 | 4.1 | 2.6 |  |
|  |  |  |  | 577.5 | 694 | 208.5 |  |  |  |  |
|  | rs2502308 | 239405036 |  | 851 | 590 | 39 | 4.1 | 2.8 | 10.2 |  |
|  |  |  |  | 887.4 | 517.2 | 75.4 |  |  |  |  |
|  | rs3003542 | 239413147 |  | 669 | 662 | 149 | 0.3 | 16.5 | 9.5 |  |
|  |  |  |  | 675.7 | 648.6 | 155.7 |  |  |  |  |
|  | rs3013384 | 239413619 |  | 334 | 999 | 147 | 44.3 | 27.6 | 21.5 |  |
|  |  |  |  | 469.4 | 728.2 | 282.4 |  |  |  |  |
|  | rs2491853 | 239416458 |  | 1067 | 369 | 44 | -0.5 | 4.9 | 13.7 |  |
|  |  |  |  | 1058.3 | 386.4 | 35.3 |  |  |  |  |
|  | rs7549760 | 239443172 |  | 695 | 716 | 69 | 8 | 17.3 | 13.2 |  |
|  |  |  |  | 749.2 | 607.6 | 123.2 |  |  |  |  |
|  | rs3013386 | 239443543 |  | 773 | 653 | 54 | 5.6 | 2.2 | 1.6 |  |
|  |  |  |  | 816.8 | 565.4 | 97.8 |  |  |  |  |
|  | rs1081091 | 239447083 |  | 768 | 607 | 105 | 0.4 | 0 | 0 |  |
|  |  |  |  | 775.8 | 591.5 | 112.8 |  |  |  |  |
|  | rs10926940 | 239461296 |  | 701 | 621 | 158 | -0.5 | -0.5 | -0.3 |  |
|  |  |  |  | 691.3 | 640.4 | 148.3 |  |  |  |  |
|  | rs2907143 | 239473476 |  | 1480 | 0 | 0 | 0 | -0.1 | 0 |  |
|  |  |  |  | 1480 | 0 | 0 |  |  |  |  |
|  | RGS7 | 239587101 | End |  |  |  |  |  |  |  |
|  | RGS7 | 239587101 | End |  |  |  |  |  |  |  |
|  | rs7531635 | 239606926 |  | 809 | 566 | 105 | -0.1 | 0 | 0 |  |
|  |  |  |  | 805.7 | 572.6 | 101.7 |  |  |  |  |
| 1q42.1 | FH | 239727526 | Start |  |  |  |  |  |  | fumarate hydratase |
|  | FH | 239749677 | End |  |  |  |  |  |  |  |
| 1q42-q44 | KMO | 239762330 | Start |  |  |  |  |  |  | kynurenine 3-monooxygenase (kynurenine 3-hydroxylase) |
|  | KMO | 239825566 | End |  |  |  |  |  |  |  |
| 2p11.2 | POLR1A | 86106962 | Start |  |  |  |  |  |  | polymerase (RNA) I polypeptide A, 194kDa |
|  | POLR1A | 86186789 | End |  |  |  |  |  |  |  |
| 2p11.2 | PTCD3 | 86186849 | Start |  |  |  |  |  |  | Pentatricopeptide repeat domain 3 |
| 2p11.2 | PTCD3 | 86186849 | Start |  |  |  |  |  |  | Pentatricopeptide repeat domain 3 |
|  | PTCD3 | 86222791 | End |  |  |  |  |  |  |  |
|  | PTCD3 | 86222791 | End |  |  |  |  |  |  |  |
| 2p11.2 | IMMT | 86224566 | Start |  |  |  |  |  |  | inner membrane protein, mitochondrial (mitofilin) |
| 2p11.2 | IMMT | 86224566 | Start |  |  |  |  |  |  | inner membrane protein, mitochondrial (mitofilin) |
|  | IMMT | 86276404 | End |  |  |  |  |  |  |  |
|  | IMMT | 86276404 | End |  |  |  |  |  |  |  |
| 2p11.2 | MRPL35 | 86280091 | Start |  |  |  |  |  |  | mitochondrial ribosomal protein L35 |
| 2p11.2 | MRPL35 | 86280091 | Start |  |  |  |  |  |  | mitochondrial ribosomal protein L35 |
|  | MRPL35 | 86293481 | End |  |  |  |  |  |  |  |
|  | MRPL35 | 86293481 | End |  |  |  |  |  |  |  |
| 2p11.2 | REEP1 | 86294633 | Start |  |  |  |  |  |  | receptor accessory protein 1 |
| 2p11.2 | REEP1 | 86294633 | Start |  |  |  |  |  |  | receptor accessory protein 1 |
|  | rs13000103 | 86367150 |  | 478 | 701 | 301 | -0.9 | -0.8 | -0.9 |  |
|  |  |  |  | 463.8 | 729.4 | 286.8 |  |  |  |  |
|  | rs2042502 | 86370907 |  | 620 | 653 | 207 | -0.9 | -0.9 | -2.4 |  |
|  |  |  |  | 605.3 | 682.4 | 192.3 |  |  |  |  |
|  | rs4832258 | 86376183 |  | 1267 | 203 | 10 | -0.1 | -2.6 | -2.7 |  |
|  |  |  |  | 1265.4 | 206.2 | 8.4 |  |  |  |  |
|  | rs2278105 | 86378174 |  | 516 | 669 | 295 | -2.3 | -2.4 | -2.5 |  |
|  |  |  |  | 488.8 | 723.5 | 267.8 |  |  |  |  |
|  | rs13017143 | 86378207 |  | 576 | 647 | 257 | -2.6 | -2.5 | -2.6 |  |
|  |  |  |  | 546.7 | 705.6 | 227.7 |  |  |  |  |
|  | rs6714362 | 86388246 |  | 515 | 667 | 298 | -2.5 | -2.6 | -2.6 |  |
|  |  |  |  | 486.5 | 724.1 | 269.5 |  |  |  |  |
|  | rs17027099 | 86390287 |  | 1479 | 0 | 1 | -0.8 | -3.1 | -2.7 |  |
|  |  |  |  | 1478 | 2 | 0 |  |  |  |  |
|  | rs4832031 | 86390638 |  | 409 | 677 | 394 | -3 | -2.6 | -2.6 |  |
|  |  |  |  | 377.5 | 739.9 | 362.5 |  |  |  |  |
|  | rs4832031 | 86390638 |  | 409 | 677 | 394 | -3 | -2.6 | -2.6 |  |
|  |  |  |  | 377.5 | 739.9 | 362.5 |  |  |  |  |
|  | rs2276625 | 86393493 |  | 453 | 687 | 340 | -1.9 | -2.6 | -2.6 |  |
|  |  |  |  | 428.7 | 735.7 | 315.7 |  |  |  |  |
|  | rs2276625 | 86393493 |  | 453 | 687 | 340 | -1.9 | -2.6 | -2.6 |  |
|  |  |  |  | 428.7 | 735.7 | 315.7 |  |  |  |  |
|  | rs11691807 | 86394679 |  | 410 | 675 | 395 | -3.1 | -3.1 | -3.1 |  |
|  |  |  |  | 377.5 | 739.9 | 362.5 |  |  |  |  |
|  | rs11691807 | 86394679 |  | 410 | 675 | 395 | -3.1 | -3.1 | -3.1 |  |
|  |  |  |  | 377.5 | 739.9 | 362.5 |  |  |  |  |
|  | rs4832261 | 86396156 |  | 409 | 676 | 395 | -3.1 | -3.1 | -3 |  |
|  |  |  |  | 377 | 739.9 | 363 |  |  |  |  |
|  | rs4832261 | 86396156 |  | 409 | 676 | 395 | -3.1 | -3.1 | -3 |  |
|  |  |  |  | 377 | 739.9 | 363 |  |  |  |  |
|  | rs4832262 | 86396792 |  | 409 | 676 | 395 | -3.1 | -3 | -2.3 |  |
|  |  |  |  | 377 | 739.9 | 363 |  |  |  |  |
|  | rs4832262 | 86396792 |  | 409 | 676 | 395 | -3.1 | -3 | -2.3 |  |
|  |  |  |  | 377 | 739.9 | 363 |  |  |  |  |
|  | rs4832263 | 86396815 |  | 464 | 677 | 339 | -2.6 | -1.9 | -1.9 |  |
|  |  |  |  | 435.1 | 734.7 | 310.1 |  |  |  |  |
|  | rs4832263 | 86396815 |  | 464 | 677 | 339 | -2.6 | -1.9 | -1.9 |  |
|  |  |  |  | 435.1 | 734.7 | 310.1 |  |  |  |  |
|  | rs17510512 | 86396973 |  | 1215 | 252 | 13 | 0 | 0 | 0.2 |  |
|  |  |  |  | 1215.1 | 251.9 | 13.1 |  |  |  |  |
|  | rs17510512 | 86396973 |  | 1215 | 252 | 13 | 0 | 0 | 0 |  |
|  |  |  |  | 1215.1 | 251.9 | 13.1 |  |  |  |  |
|  | rs17027110 | 86399763 |  | 1479 | 1 | 0 | 0 | 0.1 | -3.1 |  |
|  |  |  |  | 1479 | 1 | 0 |  |  |  |  |
|  | rs17027110 | 86399763 |  | 1479 | 1 | 0 | 0 | 0 | 0 |  |
|  |  |  |  | 1479 | 1 | 0 |  |  |  |  |
|  | rs7576102 | 86401488 |  | 1257 | 215 | 8 | 0.1 | -3.2 | -3.2 |  |
|  |  |  |  | 1258 | 213 | 9 |  |  |  |  |
|  | rs12622200 | 86410253 |  | 515 | 665 | 300 | -2.7 | -2.8 | -3.1 |  |
|  |  |  |  | 485.3 | 724.4 | 270.3 |  |  |  |  |
|  | rs10520415 | 86410396 |  | 515 | 665 | 300 | -2.7 | -3 | -3 |  |
|  |  |  |  | 485.3 | 724.4 | 270.3 |  |  |  |  |
|  | rs9309635 | 86411060 |  | 467 | 674 | 339 | -2.8 | -2.7 | 0 |  |
|  |  |  |  | 436.8 | 734.5 | 308.8 |  |  |  |  |
|  | rs11127024 | 86417111 |  | 467 | 676 | 337 | -2.6 | 0 | 0 |  |
|  |  |  |  | 437.9 | 734.3 | 307.9 |  |  |  |  |
|  | REEP1 | 86418288 | End |  |  |  |  |  |  |  |
|  | REEP1 | 86418288 | End |  |  |  |  |  |  |  |
| 2p11.2 | JMJD1A | 86521865 | Start |  |  |  |  |  |  | jumonji domain containing 1A |
| 2p11.2 | JMJD1A | 86521865 | Start |  |  |  |  |  |  | jumonji domain containing 1A |
|  | JMJD1A | 86573350 | End |  |  |  |  |  |  |  |
|  | JMJD1A | 86573350 | End |  |  |  |  |  |  |  |
| 2p24.3-p24.1 | VPS24 | 86584064 | Start |  |  |  |  |  |  | vacuolar protein sorting 24 homolog (S. cerevisiae) |
| 2p24.3-p24.1 | VPS24 | 86584064 | Start |  |  |  |  |  |  | vacuolar protein sorting 24 homolog (S. cerevisiae) |
|  | VPS24 | 86644111 | End |  |  |  |  |  |  |  |
|  | VPS24 | 86644111 | End |  |  |  |  |  |  |  |
| 2q31.1-q31.2 | MYO3B | 170742901 | Start |  |  |  |  |  |  | myosin IIIB |
|  | rs16858142 | 170995361 |  | 681 | 668 | 131 | 0.9 | 1.7 | 1.8 |  |
|  |  |  |  | 696.1 | 637.8 | 146.1 |  |  |  |  |
|  | rs11681687 | 170997780 |  | 432 | 784 | 264 | 2.3 | 2.3 | 2.3 |  |
|  |  |  |  | 458.8 | 730.5 | 290.8 |  |  |  |  |
|  | rs12613772 | 170998111 |  | 432 | 786 | 262 | 2.4 | 2.4 | 2.4 |  |
|  |  |  |  | 459.9 | 730.2 | 289.9 |  |  |  |  |
|  | rs1568269 | 170998768 |  | 432 | 786 | 262 | 2.4 | 2.4 | 2.4 |  |
|  |  |  |  | 459.9 | 730.2 | 289.9 |  |  |  |  |
|  | rs11696033 | 170999455 |  | 430 | 785 | 265 | 2.3 | 2.4 | 1.4 |  |
|  |  |  |  | 457.1 | 730.8 | 292.1 |  |  |  |  |
|  | rs9677051 | 170999743 |  | 430 | 787 | 263 | 2.5 | 1.5 | 1.4 |  |
|  |  |  |  | 458.2 | 730.6 | 291.2 |  |  |  |  |
|  | rs2113650 | 171001899 |  | 1126 | 332 | 22 | 0.1 | 1.7 | 1.4 |  |
|  |  |  |  | 1127.9 | 328.2 | 23.9 |  |  |  |  |
|  | rs2176593 | 171008483 |  | 403 | 791 | 286 | 2.4 | 1.6 | 1.9 |  |
|  |  |  |  | 430.8 | 735.4 | 313.8 |  |  |  |  |
|  | rs2176592 | 171008517 |  | 697 | 659 | 124 | 0.9 | 2.3 | 0.3 |  |
|  |  |  |  | 712 | 629.1 | 139 |  |  |  |  |
|  | rs10930420 | 171008611 |  | 372 | 803 | 305 | 3.1 | 0.4 | 0 |  |
|  |  |  |  | 404.3 | 738.5 | 337.3 |  |  |  |  |
|  | rs10930422 | 171033749 |  | 476 | 718 | 286 | -0.2 | 0 | 0 |  |
|  |  |  |  | 471.1 | 727.8 | 281.1 |  |  |  |  |
| 2q32 | HMG1L4 | 171166419 | Start |  |  |  |  |  |  | high-mobility group (nonhistone chromosomal) protein 1-like 4 |
|  | HMG1L4 | 171166984 | End |  |  |  |  |  |  |  |
|  | MYO3B | 171219237 | End |  |  |  |  |  |  |  |
| 2q37.3 | FLJ33590 | 242460559 | Start |  |  |  |  |  |  | hypothetical protein FLJ33590 |
|  | FLJ33590 | 242464155 | End |  |  |  |  |  |  |  |
| 2q37.3 | LOC285095 | 242484807 | Start |  |  |  |  |  |  | hypothetical LOC285095 |
|  | LOC285095 | 242491272 | End |  |  |  |  |  |  |  |
| 2q37.3 | FLJ40712 | 242577387 | Start |  |  |  |  |  |  | FLJ40712 protein |
|  | FLJ40712 | 242577946 | End |  |  |  |  |  |  |  |
| 2q37.3 | FLJ38379 | 242594521 | Start |  |  |  |  |  |  | hypothetical protein FLJ38379 |
|  | FLJ38379 | 242596837 | End |  |  |  |  |  |  |  |
| 2q37.3 | FLJ41327 | 242619101 | Start |  |  |  |  |  |  | FLJ41327 protein |
|  | FLJ41327 | 242619989 | End |  |  |  |  |  |  |  |
|  | rs6738966 | 242659035 |  | 528 | 658 | 294 | -3 | -2.7 | -3.3 |  |
|  |  |  |  | 496.2 | 721.5 | 262.2 |  |  |  |  |
| 2q37.3 | LOC728323 | 242679367 | Start |  |  |  |  |  |  | similar to F-box only protein 25 isoform 2 |
|  | rs7424996 | 242688002 |  | 1329 | 141 | 10 | -0.5 | -1 | -0.9 |  |
|  |  |  |  | 1323.4 | 152.2 | 4.4 |  |  |  |  |
|  | rs7423026 | 242688047 |  | 1300 | 160 | 20 | -1.4 | -1.4 | -2.3 |  |
|  |  |  |  | 1286.8 | 186.5 | 6.8 |  |  |  |  |
|  | rs6720576 | 242689246 |  | 1324 | 140 | 16 | -1.2 | -2.2 | -2.3 |  |
|  |  |  |  | 1313 | 162 | 5 |  |  |  |  |
|  | rs4973680 | 242692632 |  | 499 | 684 | 297 | -1.6 | -1.6 | -3.1 |  |
|  |  |  |  | 477.9 | 726.2 | 275.9 |  |  |  |  |
|  | rs7426040 | 242692937 |  | 1475 | 5 | 0 | 0 | -3 | -4.4 |  |
|  |  |  |  | 1475 | 5 | 0 |  |  |  |  |
|  | rs12477174 | 242695280 |  | 403 | 677 | 400 | -3 | -4.3 | -5.9 |  |
|  |  |  |  | 371.5 | 740 | 368.5 |  |  |  |  |
|  | rs11247970 | 242696895 |  | 542 | 655 | 283 | -2.9 | -4.5 | -4.5 |  |
|  |  |  |  | 510.8 | 717.3 | 251.8 |  |  |  |  |
|  | rs12468297 | 242712226 |  | 1058 | 353 | 69 | -3 | -3 | 0 |  |
|  |  |  |  | 1029.7 | 409.6 | 40.7 |  |  |  |  |
|  | rs7573042 | 242712341 |  | 1058 | 353 | 69 | -3 | 0 | 0 |  |
|  |  |  |  | 1029.7 | 409.6 | 40.7 |  |  |  |  |
|  | LOC728323 | 242713810 | End |  |  |  |  |  |  |  |
| 3p21.2-p21.1 | ITIH3 | 52803824 | Start |  |  |  |  |  |  | inter-alpha (globulin) inhibitor H3 |
|  | ITIH3 | 52818065 | End |  |  |  |  |  |  |  |
| 3p21-p14 | ITIH4 | 52822046 | Start |  |  |  |  |  |  | inter-alpha (globulin) inhibitor H4 (plasma Kallikrein-sensitive glycoprotein) |
|  | ITIH4 | 52839734 | End |  |  |  |  |  |  |  |
| 3p21.1 | MUSTN1 | 52842177 | Start |  |  |  |  |  |  | musculoskeletal, embryonic nuclear protein 1 |
|  | MUSTN1 | 52844260 | End |  |  |  |  |  |  |  |
| 3p21.1 | TMEM110 | 52848938 | Start |  |  |  |  |  |  | transmembrane protein 110 |
| 3p21.1 | LOC100132069 | 52898658 | Start |  |  |  |  |  |  | hypothetical protein LOC100132069 |
|  | LOC100132069 | 52899372 | End |  |  |  |  |  |  |  |
|  | TMEM110 | 52906587 | End |  |  |  |  |  |  |  |
| 3p21.1 | SFMBT1 | 52913667 | Start |  |  |  |  |  |  | Scm-like with four mbt domains 1 |
|  | rs2336721 | 53003415 |  | 651 | 566 | 263 | -9.9 | -16.9 | -17 |  |
|  |  |  |  | 589.4 | 689.1 | 201.4 |  |  |  |  |
|  | rs2581795 | 53013826 |  | 634 | 592 | 254 | -6.6 | -7 | -4.3 |  |
|  |  |  |  | 584.4 | 691.2 | 204.4 |  |  |  |  |
|  | rs1529544 | 53014495 |  | 637 | 589 | 254 | -6.9 | -4.2 | 0.7 |  |
|  |  |  |  | 586.3 | 690.4 | 203.3 |  |  |  |  |
|  | rs17304995 | 53045795 |  | 864 | 542 | 74 | 0.3 | 0.3 | -0.1 |  |
|  |  |  |  | 870.4 | 529.2 | 80.4 |  |  |  |  |
|  | rs11712653 | 53045915 |  | 1203 | 266 | 11 | 0.2 | 0 | -0.1 |  |
|  |  |  |  | 1206 | 260 | 14 |  |  |  |  |
|  | SFMBT1 | 53055110 | End |  |  |  |  |  |  |  |
| 3p21.1 | LOC401068 | 53055128 | Start |  |  |  |  |  |  | hypothetical gene supported by BC028186 |
|  | LOC401068 | 53057241 | End |  |  |  |  |  |  |  |
| 3p21.1 | LOC553148 | 53067124 | Start |  |  |  |  |  |  | PAI-1 mRNA binding protein pseudogene |
|  | rs11712910 | 53069234 |  | 741 | 597 | 142 | -0.6 | -0.3 | 0.1 |  |
|  |  |  |  | 730.1 | 618.8 | 131.1 |  |  |  |  |
|  | rs11915851 | 53071087 |  | 660 | 662 | 158 | 0.1 | 0.6 | 0.7 |  |
|  |  |  |  | 663.6 | 654.9 | 161.6 |  |  |  |  |
|  | LOC553148 | 53074241 | End |  |  |  |  |  |  |  |
|  | rs6770152 | 53075254 |  | 459 | 756 | 265 | 0.9 | 0.7 | 0.1 |  |
|  |  |  |  | 473.4 | 727.3 | 279.4 |  |  |  |  |
|  | rs11714876 | 53076680 |  | 1199 | 270 | 11 | 0.2 | -0.5 | 0 |  |
|  |  |  |  | 1202.4 | 263.2 | 14.4 |  |  |  |  |
|  | rs2581790 | 53076820 |  | 665 | 627 | 188 | -1.2 | 0 | 0 |  |
|  |  |  |  | 646.9 | 663.1 | 169.9 |  |  |  |  |
| 3p21.1 | RFT1 | 53099850 | Start |  |  |  |  |  |  | RFT1 homolog (S. cerevisiae) |
|  | RFT1 | 53139503 | End |  |  |  |  |  |  |  |
| 3p21.31 | PRKCD | 53170263 | Start |  |  |  |  |  |  | protein kinase C, delta |
|  | PRKCD | 53201773 | End |  |  |  |  |  |  |  |
| 3p14.3 | TKT | 53234704 | Start |  |  |  |  |  |  | transketolase (Wernicke-Korsakoff syndrome) |
|  | TKT | 53264998 | End |  |  |  |  |  |  |  |
| 3q21 | MYLK | 124813833 | Start |  |  |  |  |  |  | myosin, light chain kinase |
|  | rs3911406 | 125021533 |  | 1343 | 136 | 1 | 0.2 | 1.7 | 1.7 |  |
|  |  |  |  | 1345.2 | 131.6 | 3.2 |  |  |  |  |
|  | rs2700350 | 125032860 |  | 917 | 525 | 38 | 2 | 2 | 2 |  |
|  |  |  |  | 940 | 479 | 61 |  |  |  |  |
|  | rs2682213 | 125032892 |  | 922 | 521 | 37 | 2 | 2 | 2 |  |
|  |  |  |  | 944.8 | 475.4 | 59.8 |  |  |  |  |
|  | rs2682208 | 125036320 |  | 921 | 522 | 37 | 2 | 2 | 1.9 |  |
|  |  |  |  | 944 | 476 | 60 |  |  |  |  |
|  | rs2682206 | 125038366 |  | 921 | 522 | 37 | 2 | 1.9 | 1.9 |  |
|  |  |  |  | 944 | 476 | 60 |  |  |  |  |
|  | rs2682204 | 125039154 |  | 921 | 520 | 39 | 1.8 | 1.9 | 1.9 |  |
|  |  |  |  | 942.4 | 477.2 | 60.4 |  |  |  |  |
|  | rs1920211 | 125046146 |  | 920 | 523 | 37 | 2 | 2 | 2 |  |
|  |  |  |  | 943.2 | 476.6 | 60.2 |  |  |  |  |
|  | rs13094803 | 125047753 |  | 919 | 524 | 37 | 2 | 2 | 1.8 |  |
|  |  |  |  | 942.4 | 477.2 | 60.4 |  |  |  |  |
|  | rs4461370 | 125048862 |  | 920 | 523 | 37 | 2 | 1.8 | 1.9 |  |
|  |  |  |  | 943.2 | 476.6 | 60.2 |  |  |  |  |
|  | rs2700396 | 125049031 |  | 920 | 520 | 40 | 1.7 | 1.8 | 1.9 |  |
|  |  |  |  | 940.8 | 478.4 | 60.8 |  |  |  |  |
|  | rs7643862 | 125050318 |  | 1471 | 9 | 0 | 0 | 2.3 | 2.2 |  |
|  |  |  |  | 1471 | 9 | 0 |  |  |  |  |
|  | rs7646900 | 125051148 |  | 936 | 512 | 32 | 2.2 | 2.1 | 2 |  |
|  |  |  |  | 960 | 463.9 | 56 |  |  |  |  |
|  | rs2682218 | 125066569 |  | 920 | 523 | 37 | 2 | 1.9 | 0 |  |
|  |  |  |  | 943.2 | 476.6 | 60.2 |  |  |  |  |
|  | rs4118366 | 125066921 |  | 916 | 524 | 40 | 1.8 | 0 | 0 |  |
|  |  |  |  | 937.6 | 480.8 | 61.6 |  |  |  |  |
|  | MYLK | 125085839 | End |  |  |  |  |  |  |  |
| 3q21.1 | CCDC14 | 125115257 | Start |  |  |  |  |  |  | coiled-coil domain containing 14 |
|  | CCDC14 | 125158620 | End |  |  |  |  |  |  |  |
| 3q21.1 | ROPN1 | 125170568 | Start |  |  |  |  |  |  | ropporin, rhophilin associated protein 1 |
|  | ROPN1 | 125192889 | End |  |  |  |  |  |  |  |
| 3q29 | C3orf21 | 196270302 | Start |  |  |  |  |  |  | chromosome 3 open reading frame 21 |
|  | C3orf21 | 196473166 | End |  |  |  |  |  |  |  |
| 3q29 | CENTB2 | 196476768 | Start |  |  |  |  |  |  | centaurin, beta 2 |
|  | rs6437471 | 196533192 |  | 880 | 534 | 66 | 0.5 | 0.4 | 0.4 |  |
|  |  |  |  | 888.9 | 516.1 | 74.9 |  |  |  |  |
|  | rs6798243 | 196579829 |  | 1439 | 41 | 0 | 0 | 0.4 | 0.4 |  |
|  |  |  |  | 1439.3 | 40.4 | 0.3 |  |  |  |  |
|  | rs6801007 | 196583744 |  | 876 | 537 | 67 | 0.5 | 0.4 | 0.4 |  |
|  |  |  |  | 885.1 | 518.9 | 76.1 |  |  |  |  |
|  | rs823298 | 196602369 |  | 984 | 454 | 42 | 0.4 | 0.3 | -0.8 |  |
|  |  |  |  | 990.9 | 440.2 | 48.9 |  |  |  |  |
|  | rs2029708 | 196602407 |  | 847 | 554 | 79 | 0.3 | -0.9 | -0.1 |  |
|  |  |  |  | 853.6 | 540.7 | 85.6 |  |  |  |  |
|  | CENTB2 | 196645041 | End |  |  |  |  |  |  |  |
| 3q29 | PPP1R2 | 196722513 | Start |  |  |  |  |  |  | protein phosphatase 1, regulatory (inhibitor) subunit 2 |
|  | rs823508 | 196743569 |  | 595 | 662 | 223 | -1 | -0.1 | 1.9 |  |
|  |  |  |  | 579.4 | 693.2 | 207.4 |  |  |  |  |
|  | rs823510 | 196743818 |  | 820 | 568 | 92 | 0.2 | 5.2 | 18.9 |  |
|  |  |  |  | 823.5 | 561 | 95.5 |  |  |  |  |
|  | PPP1R2 | 196751362 | End |  |  |  |  |  |  |  |
| 3q29 | LOC100129808 | 196762717 | Start |  |  |  |  |  |  | similar to Rpl24 protein |
|  | LOC100129808 | 196763244 | End |  |  |  |  |  |  |  |
| 3q26.2-qter | APOD | 196776865 | Start |  |  |  |  |  |  | apolipoprotein D |
|  | APOD | 196792278 | End |  |  |  |  |  |  |  |
| 3q29 | LOC651714 | 196823376 | Start |  |  |  |  |  |  | similar to mucin 20 |
|  | LOC651714 | 196832773 | End |  |  |  |  |  |  |  |
| 3q29 | MIRN570 | 196911451 | Start |  |  |  |  |  |  | microRNA 570 |
|  | MIRN570 | 196911547 | End |  |  |  |  |  |  |  |
| 3q29 | LOC440993 | 196913600 | Start |  |  |  |  |  |  | hypothetical gene supported by AK128346 |
|  | LOC440993 | 196920598 | End |  |  |  |  |  |  |  |
|  | rs2688590 | 196932371 |  | 805 | 647 | 28 | 8.7 | 33.6 | 34.9 |  |
|  |  |  |  | 860.5 | 536 | 83.5 |  |  |  |  |
| 3q29 | MUC20 | 196933424 | Start |  |  |  |  |  |  | mucin 20, cell surface associated |
|  | rs2251330 | 196950134 |  | 391 | 942 | 147 | 30.1 | 37.9 | 42.9 |  |
|  |  |  |  | 502.1 | 719.9 | 258.1 |  |  |  |  |
|  | MUC20 | 196950211 | End |  |  |  |  |  |  |  |
| 3q29 | MUC4 | 196959307 | Start |  |  |  |  |  |  | mucin 4, cell surface associated |
|  | rs2113226 | 196963073 |  | 367 | 991 | 122 | 44.5 | 46.6 | 37.7 |  |
|  |  |  |  | 502.6 | 719.7 | 257.6 |  |  |  |  |
|  | rs712005 | 196963225 |  | 1033 | 435 | 12 | 2.3 | 1.3 | 0.8 |  |
|  |  |  |  | 1056.6 | 387.8 | 35.6 |  |  |  |  |
|  | rs842226 | 196968445 |  | 1267 | 203 | 10 | -0.1 | -0.2 | -0.1 |  |
|  |  |  |  | 1265.4 | 206.2 | 8.4 |  |  |  |  |
|  | rs842225 | 196969332 |  | 429 | 731 | 320 | -0.1 | 0.1 | 0 |  |
|  |  |  |  | 426.5 | 736 | 317.5 |  |  |  |  |
|  | rs2550251 | 196991485 |  | 469 | 729 | 282 | 0 | 0 | 0.3 |  |
|  |  |  |  | 469.4 | 728.2 | 282.4 |  |  |  |  |
|  | rs2688515 | 197015781 |  | 396 | 728 | 356 | -0.3 | 0.1 | 0.2 |  |
|  |  |  |  | 390.3 | 739.5 | 350.3 |  |  |  |  |
|  | rs2641773 | 197016536 |  | 361 | 760 | 359 | 0.5 | 0.5 | -0.1 |  |
|  |  |  |  | 371 | 740 | 369 |  |  |  |  |
|  | MUC4 | 197023545 | End |  |  |  |  |  |  |  |
|  | rs842461 | 197023924 |  | 935 | 479 | 66 | -0.1 | -1.9 | 0 |  |
|  |  |  |  | 932.1 | 484.9 | 63.1 |  |  |  |  |
|  | rs3107745 | 197041494 |  | 934 | 431 | 115 | -5.2 | 0 | 0 |  |
|  |  |  |  | 892.8 | 513.4 | 73.8 |  |  |  |  |
| 3q29 | TNK2 | 197074633 | Start |  |  |  |  |  |  | tyrosine kinase, non-receptor, 2 |
|  | TNK2 | 197120277 | End |  |  |  |  |  |  |  |
| 3q29 | LOC100128262 | 197122575 | Start |  |  |  |  |  |  | hypothetical protein LOC100128262 |
|  | LOC100128262 | 197123188 | End |  |  |  |  |  |  |  |
| 3q29 | LOC727978 | 197147436 | Start |  |  |  |  |  |  | hypothetical protein LOC727978 |
| 3q29 | LOC100133326 | 197148511 | Start |  |  |  |  |  |  | similar to PPP4R2 protein |
|  | LOC100133326 | 197150240 | End |  |  |  |  |  |  |  |
|  | LOC727978 | 197153362 | End |  |  |  |  |  |  |  |
| 3q29 | LOC100131360 | 197207352 | Start |  |  |  |  |  |  | similar to protein phosphatase 4 regulatory subunit 2 |
|  | LOC100131360 | 197210663 | End |  |  |  |  |  |  |  |
|  | rs1436472 | 42929230 |  | 917 | 497 | 66 | 0 | 0 | 0 |  |
|  |  |  |  | 917.8 | 495.3 | 66.8 |  |  |  |  |
|  | rs10031221 | 42946071 |  | 1480 | 0 | 0 | 0 | 0.1 | 0.2 |  |
|  |  |  |  | 1480 | 0 | 0 |  |  |  |  |
|  | rs1436475 | 42949449 |  | 914 | 500 | 66 | 0.1 | 0.2 | 0.2 |  |
|  |  |  |  | 915.5 | 497.1 | 67.5 |  |  |  |  |
|  | rs7663681 | 42950671 |  | 845 | 554 | 81 | 0.3 | 0.1 | 0.6 |  |
|  |  |  |  | 850.6 | 542.8 | 86.6 |  |  |  |  |
|  | rs6447228 | 42950699 |  | 912 | 499 | 69 | 0 | 0.4 | 0.3 |  |
|  |  |  |  | 911.5 | 499.9 | 68.5 |  |  |  |  |
|  | rs1017429 | 42954446 |  | 510 | 720 | 250 | 0.1 | 0.4 | -0.1 |  |
|  |  |  |  | 511.4 | 717.2 | 251.4 |  |  |  |  |
|  | rs1436485 | 42965638 |  | 922 | 491 | 67 | 0 | -0.4 | -0.2 |  |
|  |  |  |  | 921 | 493 | 66 |  |  |  |  |
|  | rs4412028 | 42987706 |  | 1106 | 278 | 96 | -11.3 | -11 | -10.4 |  |
|  |  |  |  | 1047.3 | 395.4 | 37.3 |  |  |  |  |
|  | rs6825107 | 42987962 |  | 1087 | 293 | 100 | -11.1 | -11.1 | -7.4 |  |
|  |  |  |  | 1028.1 | 410.9 | 41.1 |  |  |  |  |
|  | rs10213019 | 42988442 |  | 1106 | 277 | 97 | -11.6 | -7.8 | -0.8 |  |
|  |  |  |  | 1046.5 | 396.1 | 37.5 |  |  |  |  |
|  | rs4361423 | 43004531 |  | 1096 | 354 | 30 | 0 | 1 | 0.4 |  |
|  |  |  |  | 1095 | 356.1 | 29 |  |  |  |  |
|  | rs6846200 | 43048682 |  | 736 | 643 | 101 | 1.4 | 0.5 | 0.5 |  |
|  |  |  |  | 755.6 | 603.8 | 120.6 |  |  |  |  |
|  | rs4277811 | 43049698 |  | 815 | 565 | 100 | 0 | -0.1 | -0.1 |  |
|  |  |  |  | 813.9 | 567.3 | 98.9 |  |  |  |  |
|  | rs4309873 | 43051416 |  | 816 | 562 | 102 | -0.1 | -0.1 | 0.3 |  |
|  |  |  |  | 813.1 | 567.8 | 99.1 |  |  |  |  |
|  | rs13123153 | 43052059 |  | 1480 | 0 | 0 | 0 | 0.2 | 0.2 |  |
|  |  |  |  | 1480 | 0 | 0 |  |  |  |  |
|  | rs7682119 | 43057622 |  | 403 | 748 | 329 | 0.2 | 0.2 | 0 |  |
|  |  |  |  | 407.9 | 738.1 | 333.9 |  |  |  |  |
|  | rs4481267 | 43060466 |  | 1479 | 1 | 0 | 0 | 0 | 0 |  |
|  |  |  |  | 1479 | 1 | 0 |  |  |  |  |
| 4p13 | LOC441013 | 43106771 | Start |  |  |  |  |  |  | similar to ribosomal protein S2 |
|  | LOC441013 | 43107711 | End |  |  |  |  |  |  |  |
|  | rs9985504 | 82959236 |  | 535 | 749 | 196 | 1.9 | 1.8 | 1.8 |  |
|  |  |  |  | 558.9 | 701.2 | 219.9 |  |  |  |  |
|  | rs9985506 | 82959378 |  | 535 | 747 | 198 | 1.7 | 1.8 | 1.7 |  |
|  |  |  |  | 557.7 | 701.6 | 220.7 |  |  |  |  |
|  | rs4574400 | 82966023 |  | 517 | 754 | 209 | 1.8 | 1.8 | 2 |  |
|  |  |  |  | 540 | 708 | 232 |  |  |  |  |
|  | rs6535272 | 82967029 |  | 517 | 754 | 209 | 1.8 | 2.1 | 2 |  |
|  |  |  |  | 540 | 708 | 232 |  |  |  |  |
|  | rs6817583 | 82967907 |  | 633 | 708 | 139 | 2.1 | 2 | 2.2 |  |
|  |  |  |  | 658.2 | 657.6 | 164.2 |  |  |  |  |
|  | rs6839535 | 82967958 |  | 516 | 754 | 210 | 1.8 | 1.9 | 1.5 |  |
|  |  |  |  | 538.8 | 708.4 | 232.8 |  |  |  |  |
|  | rs4585291 | 82970143 |  | 386 | 786 | 308 | 1.9 | 1.4 | 1.6 |  |
|  |  |  |  | 410 | 737.9 | 332 |  |  |  |  |
|  | rs9996469 | 82971836 |  | 357 | 772 | 351 | 1 | 1 | 1.6 |  |
|  |  |  |  | 373 | 740 | 367 |  |  |  |  |
|  | rs6815101 | 82979969 |  | 477 | 755 | 248 | 1.1 | 1.6 | 0 |  |
|  |  |  |  | 493.4 | 722.3 | 264.4 |  |  |  |  |
|  | rs10010237 | 82986491 |  | 509 | 761 | 210 | 2.1 | 0 | 0 |  |
|  |  |  |  | 534.6 | 709.8 | 235.6 |  |  |  |  |
| 4q21.3 | COX5BL1 | 83059999 | Start |  |  |  |  |  |  | cytochrome c oxidase subunit Vb-like 1 |
|  | COX5BL1 | 83060664 | End |  |  |  |  |  |  |  |
| 4q25-q27 | ANK2 | 114190319 | Start |  |  |  |  |  |  | ankyrin 2, neuronal |
| 4q26 | LOC728937 | 114354544 | Start |  |  |  |  |  |  | similar to 40S ribosomal protein S26 |
|  | LOC728937 | 114355027 | End |  |  |  |  |  |  |  |
|  | rs313967 | 114363250 |  | 921 | 489 | 70 | -0.1 | 0.9 | 1.1 |  |
|  |  |  |  | 917.8 | 495.3 | 66.8 |  |  |  |  |
|  | rs12500552 | 114365930 |  | 408 | 788 | 284 | 2.2 | 1.4 | -0.6 |  |
|  |  |  |  | 434.6 | 734.8 | 310.6 |  |  |  |  |
|  | rs7656666 | 114367190 |  | 854 | 545 | 81 | 0.1 | -6.9 | -6.9 |  |
|  |  |  |  | 857.4 | 538.1 | 84.4 |  |  |  |  |
|  | rs649022 | 114367489 |  | 576 | 580 | 324 | -12.2 | -12.2 | -20.3 |  |
|  |  |  |  | 506.7 | 718.5 | 254.7 |  |  |  |  |
|  | rs594125 | 114368061 |  | 1480 | 0 | 0 | 0 | -5.8 | 0 |  |
|  |  |  |  | 1480 | 0 | 0 |  |  |  |  |
|  | rs11726138 | 114371244 |  | 735 | 550 | 195 | -5.8 | 0 | 0 |  |
|  |  |  |  | 689.3 | 641.5 | 149.3 |  |  |  |  |
|  | rs7683186 | 114371302 |  | 706 | 643 | 131 | 0.4 | 0.4 | 0.2 |  |
|  |  |  |  | 713.3 | 628.3 | 138.3 |  |  |  |  |
|  | rs7683536 | 114371396 |  | 706 | 645 | 129 | 0.4 | 0.2 | -0.3 |  |
|  |  |  |  | 714.7 | 627.5 | 137.7 |  |  |  |  |
|  | rs17045555 | 114371969 |  | 1219 | 252 | 9 | 0.2 | -3.1 | 4.4 |  |
|  |  |  |  | 1222.3 | 245.4 | 12.3 |  |  |  |  |
|  | rs684932 | 114372067 |  | 1120 | 258 | 102 | -14.1 | 0.1 | 4.4 |  |
|  |  |  |  | 1054.1 | 389.9 | 36.1 |  |  |  |  |
|  | rs6533666 | 114373140 |  | 569 | 709 | 202 | 0.3 | 0.8 | 0.7 |  |
|  |  |  |  | 576.3 | 694.5 | 209.3 |  |  |  |  |
|  | rs17476342 | 114373840 |  | 739 | 637 | 104 | 1.1 | 0.7 | 0 |  |
|  |  |  |  | 755.6 | 603.8 | 120.6 |  |  |  |  |
|  | rs17476377 | 114374956 |  | 573 | 702 | 205 | 0.2 | 0 | 0 |  |
|  |  |  |  | 576.9 | 694.2 | 208.9 |  |  |  |  |
|  | ANK2 | 114524337 | End |  |  |  |  |  |  |  |
| 5p15.2 | LOC402198 | 14957903 | Start |  |  |  |  |  |  | similar to selenophosphate synthetase 2 |
|  | LOC402198 | 15057884 | End |  |  |  |  |  |  |  |
|  | rs2455489 | 15196511 |  | 739 | 621 | 120 | 0.2 | -0.5 | -0.5 |  |
|  |  |  |  | 744.2 | 610.6 | 125.2 |  |  |  |  |
|  | rs10070527 | 15262311 |  | 479 | 684 | 317 | -1.8 | -1.8 | -1.9 |  |
|  |  |  |  | 455.4 | 731.1 | 293.4 |  |  |  |  |
|  | rs10475039 | 15263091 |  | 479 | 684 | 317 | -1.8 | -1.9 | -1.8 |  |
|  |  |  |  | 455.4 | 731.1 | 293.4 |  |  |  |  |
|  | rs2402051 | 15263941 |  | 480 | 683 | 317 | -1.9 | -1.9 | -1.9 |  |
|  |  |  |  | 456 | 731 | 293 |  |  |  |  |
|  | rs7734720 | 15277099 |  | 479 | 684 | 317 | -1.8 | -1.9 | -1.9 |  |
|  |  |  |  | 455.4 | 731.1 | 293.4 |  |  |  |  |
|  | rs10067799 | 15278284 |  | 481 | 682 | 317 | -2 | -2 | -2.1 |  |
|  |  |  |  | 456.5 | 730.9 | 292.5 |  |  |  |  |
|  | rs10042846 | 15285308 |  | 481 | 682 | 317 | -2 | -2.1 | -2.1 |  |
|  |  |  |  | 456.5 | 730.9 | 292.5 |  |  |  |  |
|  | rs10513204 | 15290740 |  | 482 | 679 | 319 | -2.2 | -2.2 | -2.2 |  |
|  |  |  |  | 456 | 731 | 293 |  |  |  |  |
|  | rs6865990 | 15292304 |  | 483 | 679 | 318 | -2.2 | -2.1 | -2.1 |  |
|  |  |  |  | 457.1 | 730.8 | 292.1 |  |  |  |  |
|  | rs173556 | 15298227 |  | 482 | 681 | 317 | -2 | -2 | -2 |  |
|  |  |  |  | 457.1 | 730.8 | 292.1 |  |  |  |  |
|  | rs173557 | 15298402 |  | 482 | 681 | 317 | -2 | -2 | -1.6 |  |
|  |  |  |  | 457.1 | 730.8 | 292.1 |  |  |  |  |
|  | rs2607527 | 15316833 |  | 479 | 682 | 319 | -2 | -1.5 | -1.6 |  |
|  |  |  |  | 454.3 | 731.4 | 294.3 |  |  |  |  |
|  | rs969856 | 15333771 |  | 479 | 695 | 306 | -1.2 | -1.6 | -1.6 |  |
|  |  |  |  | 461.6 | 729.9 | 288.6 |  |  |  |  |
|  | rs1457161 | 15335392 |  | 471 | 684 | 325 | -2 | -2 | 0 |  |
|  |  |  |  | 446.6 | 732.8 | 300.6 |  |  |  |  |
|  | rs13361669 | 15344398 |  | 1472 | 8 | 0 | 0 | 0 | 0 |  |
|  |  |  |  | 1472 | 8 | 0 |  |  |  |  |
| 5p15.1 | LOC391741 | 15503824 | Start |  |  |  |  |  |  | similar to Actin, cytoplasmic 2 (Gamma-actin) |
|  | LOC391741 | 15504943 | End |  |  |  |  |  |  |  |
| 5p15.1 | FBXL7 | 15553305 | Start |  |  |  |  |  |  | F-box and leucine-rich repeat protein 7 |
|  | rs17703976 | 15706765 |  | 1130 | 320 | 30 | -0.3 | -0.2 | 0 |  |
|  |  |  |  | 1124.4 | 331.2 | 24.4 |  |  |  |  |
|  | rs16867538 | 15733744 |  | 688 | 552 | 240 | -9.5 | -6.7 | 0.5 |  |
|  |  |  |  | 627.9 | 672.2 | 179.9 |  |  |  |  |
|  | rs17523624 | 15737270 |  | 1313 | 162 | 5 | 0 | -0.3 | 0.4 |  |
|  |  |  |  | 1313 | 162 | 5 |  |  |  |  |
|  | rs17602097 | 15741927 |  | 1152 | 301 | 27 | -0.3 | 0.2 | 0.2 |  |
|  |  |  |  | 1146.3 | 312.4 | 21.3 |  |  |  |  |
|  | rs12152734 | 15747406 |  | 714 | 634 | 132 | 0.2 | 0.2 | -0.1 |  |
|  |  |  |  | 718.2 | 625.6 | 136.2 |  |  |  |  |
|  | rs11133822 | 15753541 |  | 477 | 745 | 258 | 0.6 | -0.1 | -0.1 |  |
|  |  |  |  | 487.6 | 723.8 | 268.6 |  |  |  |  |
|  | rs7704590 | 15762298 |  | 864 | 524 | 92 | -0.4 | -0.3 | -8.3 |  |
|  |  |  |  | 856.7 | 538.7 | 84.7 |  |  |  |  |
|  | rs12519807 | 15767022 |  | 849 | 536 | 95 | -0.3 | -8.3 | -20.7 |  |
|  |  |  |  | 843 | 547.9 | 89 |  |  |  |  |
|  | rs4435862 | 15772222 |  | 669 | 546 | 265 | -12.3 | -26.5 | -26.8 |  |
|  |  |  |  | 599.6 | 684.9 | 195.6 |  |  |  |  |
|  | rs4374757 | 15772350 |  | 719 | 544 | 217 | -8.2 | -23.2 | -22.4 |  |
|  |  |  |  | 663.6 | 654.9 | 161.6 |  |  |  |  |
|  | rs16867615 | 15773075 |  | 682 | 558 | 240 | -8.9 | -7.9 | -8.3 |  |
|  |  |  |  | 624 | 674 | 182 |  |  |  |  |
|  | rs16867617 | 15773106 |  | 667 | 575 | 238 | -7.1 | -7.7 | -10.9 |  |
|  |  |  |  | 615.6 | 677.8 | 186.6 |  |  |  |  |
|  | rs7709102 | 15773451 |  | 645 | 585 | 250 | -7 | -10.3 | -2.7 |  |
|  |  |  |  | 593.9 | 687.3 | 198.9 |  |  |  |  |
|  | rs17647886 | 15773478 |  | 1290 | 166 | 24 | -1.9 | 0.1 | 0.4 |  |
|  |  |  |  | 1273.7 | 198.5 | 7.7 |  |  |  |  |
|  | rs11748700 | 15776346 |  | 457 | 747 | 276 | 0.5 | 0.1 | 0.1 |  |
|  |  |  |  | 466 | 728.9 | 285 |  |  |  |  |
|  | rs17647898 | 15782509 |  | 1316 | 156 | 8 | -0.2 | 0.1 | 0 |  |
|  |  |  |  | 1313 | 162 | 5 |  |  |  |  |
|  | rs4289558 | 15788014 |  | 476 | 741 | 263 | 0.4 | 0.3 | 0.4 |  |
|  |  |  |  | 484.2 | 724.7 | 271.2 |  |  |  |  |
|  | rs4702104 | 15799398 |  | 1449 | 30 | 1 | -0.1 | 0.2 | 0.1 |  |
|  |  |  |  | 1448.2 | 31.7 | 0.2 |  |  |  |  |
|  | rs7714352 | 15804517 |  | 881 | 529 | 70 | 0.3 | 0.1 | 0 |  |
|  |  |  |  | 886.6 | 517.8 | 75.6 |  |  |  |  |
|  | rs9637832 | 15810839 |  | 1314 | 159 | 7 | -0.1 | 0 | 0 |  |
|  |  |  |  | 1312.1 | 162.9 | 5.1 |  |  |  |  |
| 5p15.1 | MIRN887 | 15988290 | Start |  |  |  |  |  |  | microRNA 887 |
|  | MIRN887 | 15988368 | End |  |  |  |  |  |  |  |
|  | FBXL7 | 15992900 | End |  |  |  |  |  |  |  |
| 6p25.3 | OR4F1P | 50822 | Start |  |  |  |  |  |  | olfactory receptor, family 4, subfamily F, member 1 pseudogene |
|  | OR4F1P | 51956 | End |  |  |  |  |  |  |  |
| 6p25.3 | LOC646070 | 59339 | Start |  |  |  |  |  |  | similar to capicua homolog |
|  | LOC646070 | 89509 | End |  |  |  |  |  |  |  |
| 6p25.3 | LOC100132266 | 89746 | Start |  |  |  |  |  |  | similar to hCG2014367 |
|  | LOC100132266 | 91534 | End |  |  |  |  |  |  |  |
| 6p25.3 | FLJ43763 | 148313 | Start |  |  |  |  |  |  | hypothetical protein LOC642316 |
|  | FLJ43763 | 148839 | End |  |  |  |  |  |  |  |
|  | rs6927090 | 197145 |  | 1456 | 24 | 0 | 0 | 0.2 | 0.2 |  |
|  |  |  |  | 1456.1 | 23.8 | 0.1 |  |  |  |  |
|  | rs12197235 | 197772 |  | 1129 | 333 | 18 | 0.3 | 0.2 | 0.7 |  |
|  |  |  |  | 1134 | 323 | 23 |  |  |  |  |
|  | rs2181107 | 214735 |  | 1456 | 24 | 0 | 0 | 0.8 | 2.6 |  |
|  |  |  |  | 1456.1 | 23.8 | 0.1 |  |  |  |  |
|  | rs734674 | 224695 |  | 1211 | 266 | 3 | 0.7 | 2.5 | 55.5 |  |
|  |  |  |  | 1220.5 | 247 | 12.5 |  |  |  |  |
|  | rs815583 | 230695 |  | 961 | 505 | 14 | 4 | 58.2 | 43.2 |  |
|  |  |  |  | 995 | 437 | 48 |  |  |  |  |
| 6p25.3 | DUSP22 | 237101 | Start |  |  |  |  |  |  | dual specificity phosphatase 22 |
|  | rs815593 | 239457 |  | 166 | 1151 | 163 | 100.6 | 69.6 | 59.8 |  |
|  |  |  |  | 371.5 | 740 | 368.5 |  |  |  |  |
|  | rs7754000 | 248017 |  | 1201 | 262 | 17 | -0.1 | 0.2 | 0.3 |  |
|  |  |  |  | 1198.8 | 266.4 | 14.8 |  |  |  |  |
|  | SNP_A-4299501 | 260197 |  | 1322 | 158 | 0 | 0.3 | 0.8 | 69.7 |  |
|  |  |  |  | 1326.2 | 149.6 | 4.2 |  |  |  |  |
|  | rs12198312 | 268326 |  | 1071 | 381 | 28 | 0.2 | 69.5 | 68 |  |
|  |  |  |  | 1075.3 | 372.5 | 32.3 |  |  |  |  |
|  | rs11757245 | 273070 |  | 394 | 1072 | 14 | 86.9 | 80.2 | 79.5 |  |
|  |  |  |  | 584.4 | 691.2 | 204.4 |  |  |  |  |
|  | rs3800250 | 279825 |  | 1375 | 104 | 1 | 0.1 | 0.1 | 0.1 |  |
|  |  |  |  | 1375.9 | 102.2 | 1.9 |  |  |  |  |
|  | rs7763092 | 294386 |  | 1439 | 41 | 0 | 0 | 0.2 | 0.6 |  |
|  |  |  |  | 1439.3 | 40.4 | 0.3 |  |  |  |  |
|  | DUSP22 | 296355 | End |  |  |  |  |  |  |  |
|  | rs2671415 | 312109 |  | 1298 | 180 | 2 | 0.3 | 0.6 | 0.6 |  |
|  |  |  |  | 1301.7 | 172.6 | 5.7 |  |  |  |  |
|  | rs9501958 | 323970 |  | 1284 | 196 | 0 | 0.5 | 0.4 | 0 |  |
|  |  |  |  | 1290.5 | 183 | 6.5 |  |  |  |  |
|  | rs7745887 | 329546 |  | 1435 | 45 | 0 | 0 | 0 | 0 |  |
|  |  |  |  | 1435.3 | 44.3 | 0.3 |  |  |  |  |
| 6p25-p23 | IRF4 | 336760 | Start |  |  |  |  |  |  | interferon regulatory factor 4 |
|  | IRF4 | 356193 | End |  |  |  |  |  |  |  |
| 6p25.3 | EXOC2 | 430138 | Start |  |  |  |  |  |  | exocyst complex component 2 |
| 6p25.3 | LOC727827 | 469180 | Start |  |  |  |  |  |  | hypothetical protein LOC727827 |
|  | LOC727827 | 470524 | End |  |  |  |  |  |  |  |
| 6p25.3 | LOC642335 | 481199 | Start |  |  |  |  |  |  | hypothetical LOC642335 |
|  | LOC642335 | 483632 | End |  |  |  |  |  |  |  |
|  | EXOC2 | 638109 | End |  |  |  |  |  |  |  |
| 6p24.3 | HULC | 8597441 | Start |  |  |  |  |  |  | hypothetical protein LOC728655 |
|  | HULC | 8599078 | End |  |  |  |  |  |  |  |
|  | rs1207798 | 8604667 |  | 1022 | 424 | 34 | 0.4 | 0.6 | 0.6 |  |
|  |  |  |  | 1028.9 | 410.2 | 40.9 |  |  |  |  |
|  | rs7739573 | 8604903 |  | 450 | 749 | 281 | 0.5 | 0.5 | -0.1 |  |
|  |  |  |  | 459.3 | 730.4 | 290.3 |  |  |  |  |
|  | rs9393057 | 8613299 |  | 450 | 750 | 280 | 0.5 | 0 | 0.4 |  |
|  |  |  |  | 459.9 | 730.2 | 289.9 |  |  |  |  |
|  | rs3005179 | 8625282 |  | 1100 | 349 | 31 | -0.1 | 0.1 | 0.2 |  |
|  |  |  |  | 1097.5 | 353.9 | 28.5 |  |  |  |  |
|  | rs4960453 | 8637415 |  | 535 | 721 | 224 | 0.3 | -2.6 | -4.8 |  |
|  |  |  |  | 541.8 | 707.3 | 230.8 |  |  |  |  |
|  | rs3005132 | 8647461 |  | 807 | 482 | 191 | -11.1 | -4.9 | 0.2 |  |
|  |  |  |  | 742.1 | 611.8 | 126.1 |  |  |  |  |
|  | rs2987574 | 8647471 |  | 1077 | 277 | 126 | -18.6 | 0.1 | 0 |  |
|  |  |  |  | 998.3 | 434.5 | 47.3 |  |  |  |  |
|  | rs2984469 | 8649169 |  | 819 | 566 | 95 | 0.1 | 0 | 2.1 |  |
|  |  |  |  | 820.5 | 562.9 | 96.5 |  |  |  |  |
|  | rs1328860 | 8651004 |  | 667 | 656 | 157 | 0.1 | 1.6 | 2.1 |  |
|  |  |  |  | 668.9 | 652.1 | 158.9 |  |  |  |  |
|  | rs1028911 | 8651520 |  | 1004 | 351 | 125 | -11.9 | 2.3 | 2.4 |  |
|  |  |  |  | 940 | 479 | 61 |  |  |  |  |
|  | rs2984471 | 8655510 |  | 821 | 564 | 95 | 0 | 0 | 0 |  |
|  |  |  |  | 822 | 561.9 | 96 |  |  |  |  |
|  | rs17144443 | 8663987 |  | 1479 | 1 | 0 | 0 | 0 | 0 |  |
|  |  |  |  | 1479 | 1 | 0 |  |  |  |  |
|  | rs11243270 | 8665316 |  | 864 | 533 | 83 | 0 | 0 | 0 |  |
|  |  |  |  | 863.5 | 533.9 | 82.5 |  |  |  |  |
|  | rs13437391 | 8669507 |  | 1476 | 4 | 0 | 0 | 0.1 | 0 |  |
|  |  |  |  | 1476 | 4 | 0 |  |  |  |  |
|  | rs11755695 | 8683170 |  | 860 | 539 | 81 | 0.1 | 0 | 0 |  |
|  |  |  |  | 862 | 535 | 83 |  |  |  |  |
|  | rs4576244 | 9633529 |  | 1462 | 17 | 1 | -0.2 | -4.7 | -3.1 |  |
|  |  |  |  | 1461.1 | 18.9 | 0.1 |  |  |  |  |
|  | rs2103675 | 9636499 |  | 562 | 632 | 286 | -4.7 | -3.1 | -3.1 |  |
|  |  |  |  | 520.9 | 714.3 | 244.9 |  |  |  |  |
|  | rs2050144 | 9646378 |  | 562 | 661 | 257 | -1.9 | -1.8 | -1.8 |  |
|  |  |  |  | 538.2 | 708.6 | 233.2 |  |  |  |  |
|  | rs7749971 | 9646475 |  | 560 | 663 | 257 | -1.8 | -1.8 | -1.9 |  |
|  |  |  |  | 537 | 709 | 234 |  |  |  |  |
|  | rs9369824 | 9646619 |  | 561 | 661 | 258 | -1.9 | -2 | -2.1 |  |
|  |  |  |  | 537 | 709 | 234 |  |  |  |  |
|  | rs9395436 | 9647136 |  | 563 | 659 | 258 | -2 | -2.2 | -2.1 |  |
|  |  |  |  | 538.2 | 708.6 | 233.2 |  |  |  |  |
|  | rs9357594 | 9647226 |  | 517 | 671 | 292 | -2.2 | -2 | -2 |  |
|  |  |  |  | 491.1 | 722.9 | 266.1 |  |  |  |  |
|  | rs9349463 | 9647314 |  | 563 | 660 | 257 | -1.9 | -1.9 | -1.9 |  |
|  |  |  |  | 538.8 | 708.4 | 232.8 |  |  |  |  |
|  | rs9296611 | 9647458 |  | 562 | 661 | 257 | -1.9 | -1.9 | -1.9 |  |
|  |  |  |  | 538.2 | 708.6 | 233.2 |  |  |  |  |
|  | rs9395446 | 9647654 |  | 562 | 661 | 257 | -1.9 | -1.9 | -1.7 |  |
|  |  |  |  | 538.2 | 708.6 | 233.2 |  |  |  |  |
|  | rs1040518 | 9650924 |  | 561 | 662 | 257 | -1.8 | -1.7 | -2 |  |
|  |  |  |  | 537.6 | 708.8 | 233.6 |  |  |  |  |
|  | rs9395670 | 9669803 |  | 518 | 681 | 281 | -1.4 | -1.7 | -1.5 |  |
|  |  |  |  | 498 | 721 | 261 |  |  |  |  |
|  | rs9395672 | 9670135 |  | 478 | 682 | 320 | -2 | -1.5 | -1.5 |  |
|  |  |  |  | 453.2 | 731.6 | 295.2 |  |  |  |  |
|  | rs9395765 | 9678059 |  | 525 | 686 | 269 | -1 | -1.1 | 0 |  |
|  |  |  |  | 509.1 | 717.9 | 253.1 |  |  |  |  |
|  | rs7764470 | 9678450 |  | 525 | 684 | 271 | -1.1 | 0 | 0 |  |
|  |  |  |  | 507.9 | 718.2 | 253.9 |  |  |  |  |
| 6p12.2 | PKHD1 | 51588104 | Start |  |  |  |  |  |  | polycystic kidney and hepatic disease 1 (autosomal recessive) |
|  | rs1326579 | 51777521 |  | 549 | 738 | 193 | 1.5 | 1.5 | 1.4 |  |
|  |  |  |  | 569.4 | 697.2 | 213.4 |  |  |  |  |
|  | rs1326580 | 51777558 |  | 547 | 740 | 193 | 1.6 | 1.5 | 1.5 |  |
|  |  |  |  | 568.2 | 697.7 | 214.2 |  |  |  |  |
|  | rs6458788 | 51778281 |  | 552 | 736 | 192 | 1.4 | 1.4 | 1.4 |  |
|  |  |  |  | 571.9 | 696.2 | 211.9 |  |  |  |  |
|  | rs6458791 | 51778501 |  | 553 | 735 | 192 | 1.4 | 1.4 | 1.4 |  |
|  |  |  |  | 572.5 | 696 | 211.5 |  |  |  |  |
|  | rs1326581 | 51779120 |  | 552 | 735 | 193 | 1.3 | 1.4 | 1.4 |  |
|  |  |  |  | 571.3 | 696.5 | 212.3 |  |  |  |  |
|  | rs1326584 | 51779280 |  | 552 | 736 | 192 | 1.4 | 1.4 | 2.1 |  |
|  |  |  |  | 571.9 | 696.2 | 211.9 |  |  |  |  |
|  | rs4470847 | 51779309 |  | 552 | 736 | 192 | 1.4 | 2.1 | 2.1 |  |
|  |  |  |  | 571.9 | 696.2 | 211.9 |  |  |  |  |
|  | rs9395726 | 51781260 |  | 459 | 781 | 240 | 2.5 | 2.5 | 2.6 |  |
|  |  |  |  | 487.6 | 723.8 | 268.6 |  |  |  |  |
|  | rs9296661 | 51781398 |  | 459 | 781 | 240 | 2.5 | 2.6 | 0 |  |
|  |  |  |  | 487.6 | 723.8 | 268.6 |  |  |  |  |
|  | rs1326589 | 51782120 |  | 457 | 783 | 240 | 2.7 | 0 | 0 |  |
|  |  |  |  | 486.5 | 724.1 | 269.5 |  |  |  |  |
|  | PKHD1 | 52060382 | End |  |  |  |  |  |  |  |
| 6p12.3-p11.2 | ICK | 52974057 | Start |  |  |  |  |  |  | intestinal cell (MAK-like) kinase |
|  | ICK | 53034559 | End |  |  |  |  |  |  |  |
| 6p12.3-p11.2 | FBXO9 | 53037755 | Start |  |  |  |  |  |  | F-box protein 9 |
|  | FBXO9 | 53073630 | End |  |  |  |  |  |  |  |
| 6p21-p12 | GCM1 | 53099721 | Start |  |  |  |  |  |  | glial cells missing homolog 1 (Drosophila) |
|  | GCM1 | 53121586 | End |  |  |  |  |  |  |  |
| 6p12.1 | LOC647188 | 53149086 | Start |  |  |  |  |  |  | hypothetical LOC647188 |
|  | LOC647188 | 53149298 | End |  |  |  |  |  |  |  |
| 6p12.1 | LOC100130545 | 53179349 | Start |  |  |  |  |  |  | hypothetical LOC100130545 |
|  | LOC100130545 | 53181577 | End |  |  |  |  |  |  |  |
|  | rs9395848 | 53184512 |  | 1194 | 286 | 0 | 1.2 | 0.5 | 0.1 |  |
|  |  |  |  | 1207.8 | 258.4 | 13.8 |  |  |  |  |
|  | rs2817088 | 53187202 |  | 857 | 534 | 89 | -0.1 | -0.5 | -0.5 |  |
|  |  |  |  | 853.6 | 540.7 | 85.6 |  |  |  |  |
|  | rs2817090 | 53187243 |  | 570 | 683 | 227 | -0.4 | -0.5 | -0.6 |  |
|  |  |  |  | 561.4 | 700.3 | 218.4 |  |  |  |  |
|  | rs2816372 | 53193575 |  | 590 | 671 | 219 | -0.6 | -0.7 | -0.8 |  |
|  |  |  |  | 578.8 | 693.5 | 207.8 |  |  |  |  |
|  | rs7745628 | 53195543 |  | 1067 | 370 | 43 | -0.5 | -0.5 | -0.6 |  |
|  |  |  |  | 1059.1 | 385.8 | 35.1 |  |  |  |  |
|  | rs2562895 | 53201397 |  | 1239 | 228 | 13 | -0.1 | -0.1 | -2.2 |  |
|  |  |  |  | 1236.9 | 232.2 | 10.9 |  |  |  |  |
|  | rs2816364 | 53202798 |  | 1174 | 285 | 21 | -0.2 | -2.3 | -2.2 |  |
|  |  |  |  | 1171.1 | 290.9 | 18.1 |  |  |  |  |
|  | rs2562893 | 53203399 |  | 1151 | 258 | 71 | -7.2 | -7.3 | -7.4 |  |
|  |  |  |  | 1107 | 345.9 | 27 |  |  |  |  |
|  | rs2817101 | 53203522 |  | 1159 | 250 | 71 | -7.6 | -7.9 | -0.3 |  |
|  |  |  |  | 1114 | 340.1 | 26 |  |  |  |  |
|  | rs2816362 | 53203682 |  | 1174 | 235 | 71 | -8.4 | -0.8 | -0.8 |  |
|  |  |  |  | 1127 | 329 | 24 |  |  |  |  |
|  | rs7747855 | 53204641 |  | 1062 | 375 | 43 | -0.4 | -0.4 | -0.5 |  |
|  |  |  |  | 1054.9 | 389.2 | 35.9 |  |  |  |  |
|  | rs7748001 | 53204700 |  | 1062 | 375 | 43 | -0.4 | -0.5 | -0.7 |  |
|  |  |  |  | 1054.9 | 389.2 | 35.9 |  |  |  |  |
| 6p12.1 | LOC100128227 | 53208378 | Start |  |  |  |  |  |  | hypothetical LOC100128227 |
|  | LOC100128227 | 53209000 | End |  |  |  |  |  |  |  |
|  | rs2816356 | 53209407 |  | 1242 | 225 | 13 | -0.1 | -0.4 | -0.4 |  |
|  |  |  |  | 1239.6 | 229.7 | 10.6 |  |  |  |  |
|  | rs10948744 | 53213187 |  | 926 | 488 | 66 | 0 | -0.1 | -0.2 |  |
|  |  |  |  | 924.9 | 490.1 | 64.9 |  |  |  |  |
|  | rs9463885 | 53215279 |  | 1477 | 3 | 0 | 0 | -0.7 | 0.1 |  |
|  |  |  |  | 1477 | 3 | 0 |  |  |  |  |
|  | rs12183976 | 53216241 |  | 1468 | 8 | 4 | -1.3 | 0.1 | 0 |  |
|  |  |  |  | 1464 | 15.9 | 0 |  |  |  |  |
|  | rs16883632 | 53220950 |  | 1303 | 175 | 2 | 0.2 | 0 | 0 |  |
|  |  |  |  | 1306.4 | 168.2 | 5.4 |  |  |  |  |
| 6p21.1-p12.1 | ELOVL5 | 53240155 | Start |  |  |  |  |  |  | ELOVL family member 5, elongation of long chain fatty acids (FEN1/Elo2, SUR4/Elo3-like, yeast) |
| 6p12.1 | LOC647190 | 53307217 | Start |  |  |  |  |  |  | similar to 40S ribosomal protein S16 |
|  | LOC647190 | 53310780 | End |  |  |  |  |  |  |  |
|  | ELOVL5 | 53321901 | End |  |  |  |  |  |  |  |
| 6p12.1 | LOC442217 | 53327272 | Start |  |  |  |  |  |  | hypothetical LOC442217 |
|  | LOC442217 | 53327822 | End |  |  |  |  |  |  |  |
| 6p12.1 | LOC100130403 | 53351260 | Start |  |  |  |  |  |  | hypothetical LOC100130403 |
|  | LOC100130403 | 53351665 | End |  |  |  |  |  |  |  |
| 6p12.1 | NANOGP3 | 53391125 | Start |  |  |  |  |  |  | Nanog homeobox pseudogene 3 |
|  | NANOGP3 | 53392013 | End |  |  |  |  |  |  |  |
| 6q12 | NUFIP1P | 66859854 | Start |  |  |  |  |  |  | nuclear fragile X mental retardation protein interacting protein 1 pseudogene |
|  | NUFIP1P | 66861532 | End |  |  |  |  |  |  |  |
|  | rs7449962 | 67058797 |  | 860 | 540 | 80 | 0.1 | 0.1 | -0.2 |  |
|  |  |  |  | 862.8 | 534.5 | 82.8 |  |  |  |  |
|  | rs9453679 | 67058910 |  | 857 | 543 | 80 | 0.2 | -0.2 | -1 |  |
|  |  |  |  | 860.5 | 536 | 83.5 |  |  |  |  |
|  | rs17644076 | 67061694 |  | 855 | 538 | 87 | -0.1 | -0.8 | -1 |  |
|  |  |  |  | 853.6 | 540.7 | 85.6 |  |  |  |  |
|  | rs1468153 | 67065775 |  | 1014 | 386 | 80 | -3.1 | -5.4 | -5 |  |
|  |  |  |  | 984.4 | 445.3 | 50.4 |  |  |  |  |
|  | rs9453684 | 67068322 |  | 1161 | 281 | 38 | -1.4 | -1.5 | -1.8 |  |
|  |  |  |  | 1144.5 | 313.9 | 21.5 |  |  |  |  |
|  | rs16898098 | 67094275 |  | 1467 | 13 | 0 | 0 | -0.3 | -10.7 |  |
|  |  |  |  | 1467 | 12.9 | 0 |  |  |  |  |
|  | rs1708538 | 67094767 |  | 1432 | 45 | 3 | -0.3 | -10.7 | -11.5 |  |
|  |  |  |  | 1429.4 | 50.1 | 0.4 |  |  |  |  |
|  | rs1634209 | 67101187 |  | 536 | 593 | 351 | -11.7 | -11.3 | -11.2 |  |
|  |  |  |  | 468.3 | 728.4 | 283.3 |  |  |  |  |
|  | rs1708562 | 67105278 |  | 679 | 574 | 227 | -6.4 | -6.4 | -6.3 |  |
|  |  |  |  | 630.5 | 671 | 178.5 |  |  |  |  |
|  | rs1708561 | 67105350 |  | 678 | 574 | 228 | -6.5 | -6.3 | -2.2 |  |
|  |  |  |  | 629.2 | 671.6 | 179.2 |  |  |  |  |
|  | rs1708558 | 67106127 |  | 1088 | 364 | 28 | 0.1 | -0.1 | -0.2 |  |
|  |  |  |  | 1089.8 | 360.4 | 29.8 |  |  |  |  |
|  | rs1708557 | 67106320 |  | 525 | 713 | 242 | 0 | -0.1 | 0 |  |
|  |  |  |  | 525 | 712.9 | 242 |  |  |  |  |
|  | rs851861 | 67111248 |  | 987 | 439 | 54 | -0.2 | 0 | 0 |  |
|  |  |  |  | 983.5 | 445.9 | 50.5 |  |  |  |  |
| 7q31.1 | CAV2 | 115926680 | Start |  |  |  |  |  |  | caveolin 2 |
|  | CAV2 | 115935831 | End |  |  |  |  |  |  |  |
| 7q31.1 | CAV1 | 115952075 | Start |  |  |  |  |  |  | caveolin 1, caveolae protein, 22kDa |
|  | CAV1 | 115988466 | End |  |  |  |  |  |  |  |
|  | rs2237717 | 115999338 |  | 418 | 788 | 274 | 2.4 | 2.3 | 2 |  |
|  |  |  |  | 445.5 | 733 | 301.5 |  |  |  |  |
|  | rs2283053 | 116020970 |  | 966 | 471 | 43 | 0.5 | 2 | 2.1 |  |
|  |  |  |  | 975.4 | 452.2 | 52.4 |  |  |  |  |
|  | rs41735 | 116029367 |  | 415 | 784 | 281 | 2 | 2.2 | 2.2 |  |
|  |  |  |  | 440 | 733.9 | 306 |  |  |  |  |
|  | rs41736 | 116029719 |  | 419 | 787 | 274 | 2.3 | 2.2 | 1.9 |  |
|  |  |  |  | 446.1 | 732.9 | 301.1 |  |  |  |  |
|  | rs6566 | 116032369 |  | 413 | 786 | 281 | 2.2 | 1.8 | 1.8 |  |
|  |  |  |  | 438.9 | 734.1 | 306.9 |  |  |  |  |
|  | rs41741 | 116032462 |  | 655 | 671 | 154 | 0.4 | 1.7 | 1.7 |  |
|  |  |  |  | 662.9 | 655.2 | 161.9 |  |  |  |  |
|  | rs16945 | 116032605 |  | 413 | 785 | 282 | 2.1 | 2 | 2 |  |
|  |  |  |  | 438.4 | 734.2 | 307.4 |  |  |  |  |
|  | rs42336 | 116035540 |  | 414 | 785 | 281 | 2.1 | 2.1 | 2.7 |  |
|  |  |  |  | 439.5 | 734 | 306.5 |  |  |  |  |
|  | rs41746 | 116037025 |  | 415 | 785 | 280 | 2.1 | 2.7 | 2.9 |  |
|  |  |  |  | 440.6 | 733.8 | 305.6 |  |  |  |  |
|  | rs41750 | 116042951 |  | 555 | 738 | 187 | 1.6 | 3.5 | 3.4 |  |
|  |  |  |  | 576.9 | 694.2 | 208.9 |  |  |  |  |
|  | rs41751 | 116043001 |  | 480 | 780 | 220 | 3 | 2.8 | 2.9 |  |
|  |  |  |  | 511.4 | 717.2 | 251.4 |  |  |  |  |
|  | rs41752 | 116044806 |  | 479 | 777 | 224 | 2.7 | 2.7 | 3 |  |
|  |  |  |  | 508.5 | 718 | 253.5 |  |  |  |  |
|  | rs28167 | 116060034 |  | 553 | 733 | 194 | 1.2 | 3.2 | 3.1 |  |
|  |  |  |  | 571.3 | 696.5 | 212.3 |  |  |  |  |
|  | rs41775 | 116072600 |  | 507 | 773 | 200 | 3.1 | 3.1 | 2 |  |
|  |  |  |  | 539.4 | 708.2 | 232.4 |  |  |  |  |
| 7q31 | MET | 116099695 | Start |  |  |  |  |  |  | met proto-oncogene (hepatocyte growth factor receptor) |
|  | rs41796 | 116104190 |  | 1332 | 144 | 4 | 0 | 0.2 | 0 |  |
|  |  |  |  | 1331.9 | 144.2 | 3.9 |  |  |  |  |
|  | rs4808 | 116122191 |  | 869 | 536 | 75 | 0.2 | 0 | 0 |  |
|  |  |  |  | 873.5 | 527 | 79.5 |  |  |  |  |
|  | MET | 116225676 | End |  |  |  |  |  |  |  |
| 7q31.2-q31.3 | CAPZA2 | 116289799 | Start |  |  |  |  |  |  | capping protein (actin filament) muscle Z-line, alpha 2 |
|  | CAPZA2 | 116346549 | End |  |  |  |  |  |  |  |
| 8p23.3 | KBTBD11 | 1909451 | Start |  |  |  |  |  |  | kelch repeat and BTB (POZ) domain containing 11 |
|  | KBTBD11 | 1942509 | End |  |  |  |  |  |  |  |
| 8p23.3 | MYOM2 | 1980565 | Start |  |  |  |  |  |  | myomesin (M-protein) 2, 165kDa |
|  | MYOM2 | 2080787 | End |  |  |  |  |  |  |  |
|  | rs1478960 | 2137223 |  | 1316 | 160 | 4 | 0 | 0.4 | 1 |  |
|  |  |  |  | 1316.8 | 158.5 | 4.8 |  |  |  |  |
|  | rs1382608 | 2151988 |  | 372 | 772 | 336 | 1 | 1.5 | 1.1 |  |
|  |  |  |  | 388.2 | 739.6 | 352.2 |  |  |  |  |
|  | rs7838658 | 2152119 |  | 688 | 658 | 134 | 0.6 | 0.8 | 0.9 |  |
|  |  |  |  | 698.8 | 636.3 | 144.8 |  |  |  |  |
|  | rs2127175 | 2188481 |  | 1272 | 203 | 5 | 0.2 | 0.2 | -8.5 |  |
|  |  |  |  | 1274.7 | 197.7 | 7.7 |  |  |  |  |
|  | rs1037704 | 2189117 |  | 1418 | 62 | 0 | 0.1 | -10.6 | 0 |  |
|  |  |  |  | 1418.6 | 60.7 | 0.6 |  |  |  |  |
|  | rs2607684 | 2189919 |  | 753 | 509 | 218 | -11.7 | -0.1 | -0.3 |  |
|  |  |  |  | 685.8 | 643.3 | 150.8 |  |  |  |  |
|  | rs10111921 | 2200103 |  | 644 | 760 | 76 | 10.9 | 4.9 | 36.8 |  |
|  |  |  |  | 708.5 | 631 | 140.5 |  |  |  |  |
|  | rs2618872 | 2218978 |  | 1161 | 302 | 17 | 0.1 | 44.4 | 80.4 |  |
|  |  |  |  | 1163.1 | 297.9 | 19.1 |  |  |  |  |
|  | rs2605037 | 2219069 |  | 456 | 971 | 53 | 49.5 | 98.1 | 71.4 |  |
|  |  |  |  | 598.9 | 685.1 | 195.9 |  |  |  |  |
|  | rs2013135 | 2226740 |  | 278 | 1148 | 54 | 107.5 | 80.5 | 53.4 |  |
|  |  |  |  | 490.5 | 723 | 266.5 |  |  |  |  |
|  | rs315225 | 2231722 |  | 510 | 901 | 69 | 31.6 | 24.4 | 20.3 |  |
|  |  |  |  | 623.4 | 674.3 | 182.4 |  |  |  |  |
|  | rs7015044 | 2234017 |  | 513 | 834 | 133 | 13 | 10 | 17 |  |
|  |  |  |  | 584.4 | 691.2 | 204.4 |  |  |  |  |
|  | rs1159923 | 2234501 |  | 1197 | 274 | 9 | 0.3 | 19.7 | 19 |  |
|  |  |  |  | 1202.4 | 263.2 | 14.4 |  |  |  |  |
|  | rs931093 | 2243578 |  | 599 | 767 | 114 | 7.6 | 8.2 | 24.2 |  |
|  |  |  |  | 652.2 | 660.5 | 167.2 |  |  |  |  |
|  | rs2605035 | 2257735 |  | 1401 | 79 | 0 | 0.1 | 16.6 | 0.2 |  |
|  |  |  |  | 1402.1 | 76.9 | 1.1 |  |  |  |  |
|  | rs6558636 | 2257979 |  | 589 | 814 | 77 | 16.8 | 0 | -2.7 |  |
|  |  |  |  | 670.3 | 651.4 | 158.3 |  |  |  |  |
|  | rs6558637 | 2258129 |  | 594 | 534 | 352 | -21.5 | -8.5 | -5.9 |  |
|  |  |  |  | 500.9 | 720.2 | 258.9 |  |  |  |  |
|  | rs4876160 | 2258259 |  | 907 | 498 | 75 | -0.2 | 0.3 | 0.1 |  |
|  |  |  |  | 902.9 | 506.1 | 70.9 |  |  |  |  |
|  | rs1614403 | 2258689 |  | 1145 | 325 | 10 | 0.7 | 0.5 | 15.8 |  |
|  |  |  |  | 1155.1 | 304.8 | 20.1 |  |  |  |  |
|  | rs2260185 | 2259833 |  | 1411 | 69 | 0 | 0.1 | 14.9 | 16.9 |  |
|  |  |  |  | 1411.8 | 67.4 | 0.8 |  |  |  |  |
|  | rs11136507 | 2259919 |  | 663 | 767 | 50 | 15.3 | 17.7 | 16.1 |  |
|  |  |  |  | 740 | 613.1 | 127 |  |  |  |  |
|  | rs315237 | 2268999 |  | 1319 | 160 | 1 | 0.3 | 1 | 1 |  |
|  |  |  |  | 1322.4 | 153.1 | 4.4 |  |  |  |  |
|  | rs1244622 | 2275672 |  | 1209 | 271 | 0 | 1.1 | 1.1 | 1.9 |  |
|  |  |  |  | 1221.4 | 246.2 | 12.4 |  |  |  |  |
|  | rs6558647 | 2277096 |  | 1480 | 0 | 0 | 0 | 1 | 1 |  |
|  |  |  |  | 1480 | 0 | 0 |  |  |  |  |
|  | rs6558648 | 2277246 |  | 410 | 768 | 302 | 1 | 1 | 0 |  |
|  |  |  |  | 426 | 736.1 | 318 |  |  |  |  |
|  | rs6981064 | 2277300 |  | 411 | 768 | 301 | 1 | 0 | 0 |  |
|  |  |  |  | 427 | 735.9 | 317 |  |  |  |  |
| 8p23.2 | CSMD1 | 2780282 | Start |  |  |  |  |  |  | CUB and Sushi multiple domains 1 |
|  | rs1625889 | 4073171 |  | 530 | 747 | 203 | 1.6 | 2.3 | 2.2 |  |
|  |  |  |  | 551.6 | 703.9 | 224.6 |  |  |  |  |
|  | rs1624827 | 4073351 |  | 1373 | 107 | 0 | 0.2 | 2.1 | 2.1 |  |
|  |  |  |  | 1374.9 | 103.1 | 1.9 |  |  |  |  |
|  | rs7834905 | 4075822 |  | 587 | 726 | 167 | 1.8 | 2.2 | 2.2 |  |
|  |  |  |  | 609.8 | 680.4 | 189.8 |  |  |  |  |
|  | rs13280268 | 4075964 |  | 486 | 768 | 226 | 2.1 | 1.9 | 2 |  |
|  |  |  |  | 511.4 | 717.2 | 251.4 |  |  |  |  |
|  | rs1700049 | 4076226 |  | 547 | 738 | 195 | 1.4 | 1.4 | 2.2 |  |
|  |  |  |  | 566.9 | 698.1 | 214.9 |  |  |  |  |
|  | rs7822178 | 4076294 |  | 558 | 734 | 188 | 1.4 | 2.1 | 2 |  |
|  |  |  |  | 578.1 | 693.8 | 208.1 |  |  |  |  |
|  | rs11992222 | 4076524 |  | 432 | 784 | 264 | 2.3 | 2.3 | 2.3 |  |
|  |  |  |  | 458.8 | 730.5 | 290.8 |  |  |  |  |
|  | rs11992223 | 4076540 |  | 481 | 772 | 227 | 2.3 | 2.3 | 2.3 |  |
|  |  |  |  | 507.9 | 718.2 | 253.9 |  |  |  |  |
|  | rs11993028 | 4076646 |  | 432 | 785 | 263 | 2.3 | 2.4 | 0 |  |
|  |  |  |  | 459.3 | 730.4 | 290.3 |  |  |  |  |
|  | rs13272378 | 4078056 |  | 438 | 784 | 258 | 2.4 | 0 | 0 |  |
|  |  |  |  | 465.5 | 729.1 | 285.5 |  |  |  |  |
|  | CSMD1 | 4839736 | End |  |  |  |  |  |  |  |
| 8p23.1 | FAM86B1 | 12077022 | Start |  |  |  |  |  |  | family with sequence similarity 86, member B1 |
|  | FAM86B1 | 12089033 | End |  |  |  |  |  |  |  |
| 8p23.1 | DEFB130 | 12212843 | Start |  |  |  |  |  |  | defensin, beta 130 |
|  | DEFB130 | 12220196 | End |  |  |  |  |  |  |  |
| 8p23.1 | ZNF705C | 12237365 | Start |  |  |  |  |  |  | zinc finger protein 705C |
|  | ZNF705C | 12263733 | End |  |  |  |  |  |  |  |
| 8p23.1 | LOC100133172 | 12263894 | Start |  |  |  |  |  |  | similar to hCG1990697 |
|  | LOC100133172 | 12264754 | End |  |  |  |  |  |  |  |
| 8p23.1 | LOC649346 | 12276213 | Start |  |  |  |  |  |  | similar to deubiquitinating enzyme 3 |
|  | LOC649346 | 12277743 | End |  |  |  |  |  |  |  |
| 8p23.1 | LOC649352 | 12280552 | Start |  |  |  |  |  |  | similar to deubiquitinating enzyme 3 |
|  | LOC649352 | 12282461 | End |  |  |  |  |  |  |  |
|  | rs3976518 | 12285820 |  | 1229 | 251 | 0 | 0.9 | 5.4 | 5.2 |  |
|  |  |  |  | 1239.6 | 229.7 | 10.6 |  |  |  |  |
|  | rs2739979 | 12285893 |  | 747 | 682 | 51 | 7.7 | 7.4 | 7.4 |  |
|  |  |  |  | 799.8 | 576.3 | 103.8 |  |  |  |  |
| 8p23.1 | LOC100128995 | 12289374 | Start |  |  |  |  |  |  | hypothetical protein LOC100128995 |
|  | LOC100128995 | 12291715 | End |  |  |  |  |  |  |  |
| 8p23.1 | FAM90A25P | 12316402 | Start |  |  |  |  |  |  | family with sequence similarity 90, member A25 pseudogene |
|  | FAM90A25P | 12322773 | End |  |  |  |  |  |  |  |
| 8p23.1 | FAM86B2 | 12325843 | Start |  |  |  |  |  |  | family with sequence similarity 86, member B2 |
|  | FAM86B2 | 12338262 | End |  |  |  |  |  |  |  |
| 8p23.1 | LOC646344 | 12350171 | Start |  |  |  |  |  |  | similar to sphingomyelinase, intestinal alkaline |
|  | LOC646344 | 12370407 | End |  |  |  |  |  |  |  |
| 8p23.1 | LOC100127885 | 12378728 | Start |  |  |  |  |  |  | similar to liver-related low express protein 1 |
|  | LOC100127885 | 12378901 | End |  |  |  |  |  |  |  |
|  | rs9325708 | 12447312 |  | 1477 | 1 | 2 | -1.1 | -1.1 | -1.1 |  |
|  |  |  |  | 1475 | 5 | 0 |  |  |  |  |
| 8p23.1 | LOC100131718 | 12448333 | Start |  |  |  |  |  |  | hypothetical LOC100131718 |
|  | LOC100131718 | 12448991 | End |  |  |  |  |  |  |  |
|  | rs2681140 | 12466379 |  | 1480 | 0 | 0 | 0 | 0 | 0.5 |  |
|  |  |  |  | 1480 | 0 | 0 |  |  |  |  |
| 8p23.1 | LOC100131581 | 12480186 | Start |  |  |  |  |  |  | hypothetical LOC100131581 |
|  | LOC100131581 | 12481558 | End |  |  |  |  |  |  |  |
|  | rs607884 | 12487533 |  | 1480 | 0 | 0 | 0 | 0.5 | 0.5 |  |
|  |  |  |  | 1480 | 0 | 0 |  |  |  |  |
| 8p23.1 | LOC729732 | 12496850 | Start |  |  |  |  |  |  | hypothetical protein LOC729732 |
| 8p23.1 | LOC100132309 | 12509163 | Start |  |  |  |  |  |  | hypothetical LOC100132309 |
|  | LOC100132309 | 12509772 | End |  |  |  |  |  |  |  |
|  | rs7465010 | 12517929 |  | 614 | 693 | 173 | 0.5 | 0.5 | 0.5 |  |
|  |  |  |  | 623.4 | 674.3 | 182.4 |  |  |  |  |
|  | rs6530842 | 12520366 |  | 1480 | 0 | 0 | 0 | 0 | 10.7 |  |
|  |  |  |  | 1480 | 0 | 0 |  |  |  |  |
|  | rs1715410 | 12526350 |  | 1480 | 0 | 0 | 0 | 10.7 | -0.1 |  |
|  |  |  |  | 1480 | 0 | 0 |  |  |  |  |
|  | rs680850 | 12537556 |  | 728 | 708 | 44 | 10.7 | -0.1 | 0 |  |
|  |  |  |  | 791 | 581.9 | 107 |  |  |  |  |
|  | LOC729732 | 12567490 | End |  |  |  |  |  |  |  |
| 8p23.1 | OR7E8P | 12585920 | Start |  |  |  |  |  |  | olfactory receptor, family 7, subfamily E, member 8 pseudogene |
| 8p23.1 | LOC442381 | 12585982 | Start |  |  |  |  |  |  | similar to Protein C11orf2 (Another new gene 2 protein) |
|  | OR7E8P | 12587145 | End |  |  |  |  |  |  |  |
|  | LOC442381 | 12587582 | End |  |  |  |  |  |  |  |
| 8p23.1 | OR7E15P | 12598250 | Start |  |  |  |  |  |  | olfactory receptor, family 7, subfamily E, member 15 pseudogene |
|  | OR7E15P | 12598905 | End |  |  |  |  |  |  |  |
| 8p23.1 | OR7E10P | 12604832 | Start |  |  |  |  |  |  | olfactory receptor, family 7, subfamily E, member 10 pseudogene |
|  | OR7E10P | 12606018 | End |  |  |  |  |  |  |  |
| 8p23.1 | LONRF1 | 12623777 | Start |  |  |  |  |  |  | LON peptidase N-terminal domain and ring finger 1 |
|  | rs3802268 | 12630632 |  | 709 | 534 | 237 | -11.1 | -9.9 | 0 |  |
|  |  |  |  | 643.6 | 664.7 | 171.6 |  |  |  |  |
|  | rs6995647 | 12632654 |  | 1447 | 33 | 0 | 0 | 0 | 0 |  |
|  |  |  |  | 1447.2 | 32.6 | 0.2 |  |  |  |  |
|  | LONRF1 | 12657363 | End |  |  |  |  |  |  |  |
| 8q23.3 | CSMD3 | 113304333 | Start |  |  |  |  |  |  | CUB and Sushi multiple domains 3 |
|  | rs16883437 | 113407962 |  | 900 | 498 | 82 | -0.4 | -0.4 | -1.7 |  |
|  |  |  |  | 892 | 513.9 | 74 |  |  |  |  |
|  | rs16883441 | 113408633 |  | 1479 | 1 | 0 | 0 | -2.1 | -2.1 |  |
|  |  |  |  | 1479 | 1 | 0 |  |  |  |  |
|  | rs7462540 | 113435341 |  | 463 | 683 | 334 | -2.1 | -2.2 | -2.2 |  |
|  |  |  |  | 437.3 | 734.4 | 308.3 |  |  |  |  |
|  | rs1492678 | 113443580 |  | 464 | 682 | 334 | -2.2 | -2.2 | -2.9 |  |
|  |  |  |  | 437.9 | 734.3 | 307.9 |  |  |  |  |
|  | rs3950676 | 113443849 |  | 463 | 683 | 334 | -2.1 | -2.8 | -3.2 |  |
|  |  |  |  | 437.3 | 734.4 | 308.3 |  |  |  |  |
|  | rs4876466 | 113445000 |  | 874 | 491 | 115 | -2.5 | -6.8 | -3.2 |  |
|  |  |  |  | 846.8 | 545.4 | 87.8 |  |  |  |  |
|  | rs4876281 | 113445071 |  | 447 | 647 | 386 | -5.7 | -3.7 | -3.7 |  |
|  |  |  |  | 401.1 | 738.7 | 340.1 |  |  |  |  |
|  | SNP_A-2033349 | 113445554 |  | 465 | 682 | 333 | -2.2 | -2.2 | -2.1 |  |
|  |  |  |  | 438.9 | 734.1 | 306.9 |  |  |  |  |
|  | SNP_A-2034295 | 113445678 |  | 465 | 682 | 333 | -2.2 | -2.1 | -2 |  |
|  |  |  |  | 438.9 | 734.1 | 306.9 |  |  |  |  |
|  | rs4876468 | 113449291 |  | 462 | 685 | 333 | -2 | -2 | -2 |  |
|  |  |  |  | 437.3 | 734.4 | 308.3 |  |  |  |  |
|  | rs4563913 | 113451969 |  | 465 | 683 | 332 | -2.1 | -2 | -1.7 |  |
|  |  |  |  | 439.5 | 734 | 306.5 |  |  |  |  |
|  | rs1873746 | 113452239 |  | 467 | 684 | 329 | -2 | -1.7 | -1.4 |  |
|  |  |  |  | 442.2 | 733.6 | 304.2 |  |  |  |  |
|  | rs17640016 | 113467267 |  | 879 | 511 | 90 | -0.5 | -1.2 | 0 |  |
|  |  |  |  | 869.7 | 529.7 | 80.7 |  |  |  |  |
|  | rs6994009 | 113469659 |  | 404 | 708 | 368 | -1 | 0 | 0 |  |
|  |  |  |  | 388.2 | 739.6 | 352.2 |  |  |  |  |
|  | CSMD3 | 114518418 | End |  |  |  |  |  |  |  |
| 8q24.11-q24.13 | EXT1 | 118880783 | Start |  |  |  |  |  |  | exostoses (multiple) 1 |
|  | EXT1 | 119193239 | End |  |  |  |  |  |  |  |
| 8q24.12 | SAMD12 | 119270875 | Start |  |  |  |  |  |  | sterile alpha motif domain containing 12 |
|  | rs11562802 | 119281773 |  | 1205 | 262 | 13 | 0.1 | 1.8 | 1.9 |  |
|  |  |  |  | 1206 | 260 | 14 |  |  |  |  |
|  | rs2451164 | 119283555 |  | 521 | 757 | 202 | 2.1 | 2.2 | 3.5 |  |
|  |  |  |  | 546.7 | 705.6 | 227.7 |  |  |  |  |
|  | rs2451163 | 119283577 |  | 518 | 759 | 203 | 2.2 | 3.5 | 3.4 |  |
|  |  |  |  | 544.3 | 706.5 | 229.3 |  |  |  |  |
|  | rs2514747 | 119284010 |  | 469 | 801 | 210 | 4.9 | 3.3 | 3.4 |  |
|  |  |  |  | 510.8 | 717.3 | 251.8 |  |  |  |  |
|  | rs2514948 | 119284126 |  | 520 | 756 | 204 | 2 | 2.1 | 2.1 |  |
|  |  |  |  | 544.9 | 706.3 | 228.9 |  |  |  |  |
|  | rs2514949 | 119284165 |  | 521 | 757 | 202 | 2.1 | 2.2 | 2.2 |  |
|  |  |  |  | 546.7 | 705.6 | 227.7 |  |  |  |  |
|  | rs2514752 | 119292130 |  | 518 | 759 | 203 | 2.2 | 2.2 | 2.1 |  |
|  |  |  |  | 544.3 | 706.5 | 229.3 |  |  |  |  |
|  | rs10955866 | 119295568 |  | 521 | 757 | 202 | 2.1 | 2.1 | 2.1 |  |
|  |  |  |  | 546.7 | 705.6 | 227.7 |  |  |  |  |
|  | rs10808496 | 119298314 |  | 522 | 755 | 203 | 2 | 2.1 | 1.9 |  |
|  |  |  |  | 546.7 | 705.6 | 227.7 |  |  |  |  |
|  | rs2451168 | 119305578 |  | 529 | 756 | 195 | 2.3 | 1.9 | 1.9 |  |
|  |  |  |  | 555.8 | 702.3 | 221.8 |  |  |  |  |
|  | rs2451138 | 119307654 |  | 795 | 607 | 78 | 1.5 | 1.5 | 1.5 |  |
|  |  |  |  | 815.3 | 566.3 | 98.3 |  |  |  |  |
|  | rs12680308 | 119309206 |  | 795 | 607 | 78 | 1.5 | 1.5 | 0 |  |
|  |  |  |  | 815.3 | 566.3 | 98.3 |  |  |  |  |
|  | rs7017744 | 119314555 |  | 1478 | 2 | 0 | 0 | 0 | 0 |  |
|  |  |  |  | 1478 | 2 | 0 |  |  |  |  |
|  | SAMD12 | 119703365 | End |  |  |  |  |  |  |  |
| 9p23-p24.3 | PTPRD | 8304246 | Start |  |  |  |  |  |  | protein tyrosine phosphatase, receptor type, D |
|  | rs17588757 | 9052189 |  | 1094 | 349 | 37 | -0.4 | -0.3 | -0.2 |  |
|  |  |  |  | 1087.2 | 362.6 | 30.2 |  |  |  |  |
|  | rs16928863 | 9055718 |  | 1291 | 180 | 9 | -0.1 | 0 | 0 |  |
|  |  |  |  | 1288.6 | 184.8 | 6.6 |  |  |  |  |
|  | rs324474 | 9067555 |  | 867 | 536 | 77 | 0.1 | 0.1 | 0.9 |  |
|  |  |  |  | 870.4 | 529.2 | 80.4 |  |  |  |  |
|  | rs324475 | 9067607 |  | 866 | 536 | 78 | 0.1 | 0.9 | 0.8 |  |
|  |  |  |  | 868.9 | 530.2 | 80.9 |  |  |  |  |
|  | rs674362 | 9075811 |  | 504 | 752 | 224 | 1.3 | 1 | 0.7 |  |
|  |  |  |  | 523.2 | 713.5 | 243.2 |  |  |  |  |
| 9p23 | RPS26P3 | 9080773 | Start |  |  |  |  |  |  | ribosomal protein S26 pseudogene 3 |
|  | RPS26P3 | 9081423 | End |  |  |  |  |  |  |  |
|  | rs10977479 | 9082345 |  | 1185 | 288 | 7 | 0.6 | 0.5 | 1 |  |
|  |  |  |  | 1193.4 | 271.2 | 15.4 |  |  |  |  |
|  | rs7024971 | 9088121 |  | 1156 | 311 | 13 | 0.4 | 0.9 | 0.3 |  |
|  |  |  |  | 1162.2 | 298.6 | 19.2 |  |  |  |  |
|  | rs324542 | 9092409 |  | 774 | 598 | 108 | 0.2 | -1.2 | -1.2 |  |
|  |  |  |  | 777.9 | 590.2 | 111.9 |  |  |  |  |
|  | rs10977509 | 9121375 |  | 1447 | 0 | 33 | -15.7 | -15.7 | -5.4 |  |
|  |  |  |  | 1414.7 | 64.5 | 0.7 |  |  |  |  |
|  | rs10977514 | 9125223 |  | 1448 | 0 | 32 | -15.2 | -5.2 | -1.5 |  |
|  |  |  |  | 1416.7 | 62.6 | 0.7 |  |  |  |  |
|  | rs7043302 | 9129951 |  | 1349 | 127 | 4 | -0.1 | -0.3 | -0.4 |  |
|  |  |  |  | 1348.1 | 128.8 | 3.1 |  |  |  |  |
|  | rs2028782 | 9131628 |  | 548 | 696 | 236 | -0.2 | -0.4 | -0.5 |  |
|  |  |  |  | 542.4 | 707.1 | 230.4 |  |  |  |  |
|  | rs957155 | 9132828 |  | 1108 | 340 | 32 | -0.2 | -0.3 | -0.2 |  |
|  |  |  |  | 1103.6 | 348.9 | 27.6 |  |  |  |  |
|  | rs10977527 | 9134115 |  | 873 | 523 | 84 | -0.1 | 0 | 0.1 |  |
|  |  |  |  | 869.7 | 529.7 | 80.7 |  |  |  |  |
|  | rs1992671 | 9136140 |  | 549 | 707 | 224 | 0.1 | 0.1 | 0.1 |  |
|  |  |  |  | 550.3 | 704.3 | 225.3 |  |  |  |  |
|  | rs10121402 | 9136300 |  | 544 | 709 | 227 | 0.1 | 0.1 | -0.7 |  |
|  |  |  |  | 545.5 | 706.1 | 228.5 |  |  |  |  |
|  | rs1445208 | 9152669 |  | 536 | 714 | 230 | 0.1 | -0.7 | 0 |  |
|  |  |  |  | 538.8 | 708.4 | 232.8 |  |  |  |  |
|  | rs7847947 | 9166870 |  | 961 | 432 | 87 | -2.3 | 0 | 0 |  |
|  |  |  |  | 936 | 481.9 | 62 |  |  |  |  |
|  | PTPRD | 10602509 | End |  |  |  |  |  |  |  |
| 10p12 | PTER | 16518973 | Start |  |  |  |  |  |  | phosphotriesterase related |
|  | rs7074783 | 16551080 |  | 840 | 442 | 198 | -16.4 | -8.6 | 0.1 |  |
|  |  |  |  | 760.6 | 600.8 | 118.6 |  |  |  |  |
|  | rs7900628 | 16559583 |  | 430 | 736 | 314 | 0 | 1.1 | 0.9 |  |
|  |  |  |  | 430.3 | 735.5 | 314.3 |  |  |  |  |
|  | rs7915486 | 16559769 |  | 601 | 721 | 158 | 1.9 | 1.4 | 1.3 |  |
|  |  |  |  | 624.7 | 673.7 | 181.7 |  |  |  |  |
|  | rs1411876 | 16567178 |  | 407 | 738 | 335 | 0 | 0 | -0.1 |  |
|  |  |  |  | 406.9 | 738.2 | 334.9 |  |  |  |  |
|  | rs3802555 | 16568857 |  | 1355 | 122 | 3 | 0 | 0.1 | 0.1 |  |
|  |  |  |  | 1354.8 | 122.5 | 2.8 |  |  |  |  |
|  | rs10508512 | 16572580 |  | 1243 | 230 | 7 | 0.2 | 0.2 | 0.1 |  |
|  |  |  |  | 1246.1 | 223.9 | 10.1 |  |  |  |  |
|  | rs17138721 | 16575257 |  | 1243 | 229 | 8 | 0.1 | 0 | 0 |  |
|  |  |  |  | 1245.1 | 224.7 | 10.1 |  |  |  |  |
|  | rs17138741 | 16581069 |  | 1345 | 131 | 4 | 0 | 0 | -21.8 |  |
|  |  |  |  | 1344.3 | 132.5 | 3.3 |  |  |  |  |
|  | rs11254031 | 16581777 |  | 1429 | 50 | 1 | -0.1 | -26.1 | 0 |  |
|  |  |  |  | 1428.5 | 51.1 | 0.5 |  |  |  |  |
|  | rs10904756 | 16587840 |  | 675 | 490 | 315 | -26.2 | 0 | 0 |  |
|  |  |  |  | 571.9 | 696.2 | 211.9 |  |  |  |  |
|  | PTER | 16595742 | End |  |  |  |  |  |  |  |
| 10p13 | C1QL3 | 16595748 | Start |  |  |  |  |  |  | complement component 1, q subcomponent-like 3 |
|  | C1QL3 | 16604010 | End |  |  |  |  |  |  |  |
| 10p13 | RSU1 | 16672621 | Start |  |  |  |  |  |  | Ras suppressor protein 1 |
|  | RSU1 | 16899459 | End |  |  |  |  |  |  |  |
| 10p12.33 | FAM23B | 18081224 | Start |  |  |  |  |  |  | family with sequence similarity 23, member B |
|  | FAM23B | 18129861 | End |  |  |  |  |  |  |  |
| 10p12.33 | MRC1 | 18138358 | Start |  |  |  |  |  |  | mannose receptor, C type 1 |
|  | MRC1 | 18240097 | End |  |  |  |  |  |  |  |
|  | rs10508553 | 18270265 |  | 992 | 440 | 48 | 0 | 0 | 0 |  |
|  |  |  |  | 992.5 | 438.9 | 48.5 |  |  |  |  |
|  | rs16916669 | 18277976 |  | 1476 | 0 | 4 | -2.3 | -0.1 | -0.1 |  |
|  |  |  |  | 1472 | 8 | 0 |  |  |  |  |
| 10p12.33 | SLC39A12 | 18280827 | Start |  |  |  |  |  |  | solute carrier family 39 (zinc transporter), member 12 |
|  | rs10508554 | 18282641 |  | 738 | 613 | 129 | 0 | 0 | -0.1 |  |
|  |  |  |  | 737.1 | 614.7 | 128.1 |  |  |  |  |
|  | rs1887218 | 18287669 |  | 1480 | 0 | 0 | 0 | -0.1 | -0.9 |  |
|  |  |  |  | 1480 | 0 | 0 |  |  |  |  |
|  | rs7085542 | 18288101 |  | 742 | 608 | 130 | -0.1 | -0.9 | -0.9 |  |
|  |  |  |  | 739.3 | 613.5 | 127.3 |  |  |  |  |
|  | rs571979 | 18291613 |  | 590 | 665 | 225 | -0.9 | -0.9 | -0.3 |  |
|  |  |  |  | 575 | 695 | 210 |  |  |  |  |
|  | rs16916691 | 18293357 |  | 1480 | 0 | 0 | 0 | 0.1 | -4.6 |  |
|  |  |  |  | 1480 | 0 | 0 |  |  |  |  |
|  | rs1277532 | 18304631 |  | 388 | 743 | 349 | 0.1 | -4.6 | -8.2 |  |
|  |  |  |  | 389.8 | 739.5 | 350.8 |  |  |  |  |
|  | rs2497828 | 18307982 |  | 592 | 541 | 347 | -19.8 | -28.8 | -9.6 |  |
|  |  |  |  | 502.6 | 719.7 | 257.6 |  |  |  |  |
|  | rs2497830 | 18308217 |  | 740 | 474 | 266 | -22.5 | -0.3 | -0.9 |  |
|  |  |  |  | 645 | 664.1 | 171 |  |  |  |  |
|  | rs10827981 | 18308228 |  | 1061 | 388 | 31 | 0.2 | -0.4 | -0.6 |  |
|  |  |  |  | 1064.2 | 381.6 | 34.2 |  |  |  |  |
|  | rs11011857 | 18310728 |  | 971 | 445 | 64 | -0.5 | -1.1 | -1.8 |  |
|  |  |  |  | 962.5 | 462.1 | 55.5 |  |  |  |  |
|  | rs10508555 | 18316688 |  | 528 | 701 | 251 | -0.3 | -0.3 | -0.6 |  |
|  |  |  |  | 521.5 | 714.1 | 244.5 |  |  |  |  |
|  | rs2497755 | 18322673 |  | 1202 | 255 | 23 | -0.5 | -0.6 | -0.4 |  |
|  |  |  |  | 1194.3 | 270.4 | 15.3 |  |  |  |  |
|  | rs7099037 | 18326599 |  | 484 | 713 | 283 | -0.3 | -0.1 | -0.1 |  |
|  |  |  |  | 477.3 | 726.4 | 276.3 |  |  |  |  |
|  | rs7079066 | 18326645 |  | 545 | 709 | 226 | 0.1 | 0.1 | 0 |  |
|  |  |  |  | 546.7 | 705.6 | 227.7 |  |  |  |  |
|  | rs11818989 | 18327820 |  | 1446 | 34 | 0 | 0 | -0.3 | 0 |  |
|  |  |  |  | 1446.2 | 33.6 | 0.2 |  |  |  |  |
|  | rs2478574 | 18328638 |  | 1200 | 259 | 21 | -0.4 | 0 | 0 |  |
|  |  |  |  | 1194.3 | 270.4 | 15.3 |  |  |  |  |
| 10p12.33 | LOC100129213 | 18330721 | Start |  |  |  |  |  |  | hypothetical protein LOC100129213 |
|  | LOC100129213 | 18358275 | End |  |  |  |  |  |  |  |
|  | SLC39A12 | 18372218 | End |  |  |  |  |  |  |  |
| 10p12 | CACNB2 | 18469612 | Start |  |  |  |  |  |  | calcium channel, voltage-dependent, beta 2 subunit |
|  | CACNB2 | 18870694 | End |  |  |  |  |  |  |  |
|  | rs1542379 | 11801092 |  | 948 | 471 | 61 | -0.1 | -18.5 | -3.8 |  |
|  |  |  |  | 946.4 | 474.2 | 59.4 |  |  |  |  |
|  | rs10831682 | 11801881 |  | 707 | 493 | 280 | -21.4 | -5.7 | -0.8 |  |
|  |  |  |  | 614.3 | 678.4 | 187.3 |  |  |  |  |
|  | rs4910425 | 11804807 |  | 657 | 655 | 168 | -0.1 | 0.4 | 0.6 |  |
|  |  |  |  | 654.9 | 659.2 | 165.9 |  |  |  |  |
|  | rs753020 | 11806675 |  | 673 | 667 | 140 | 0.6 | 0.6 | 0.6 |  |
|  |  |  |  | 684.5 | 644 | 151.5 |  |  |  |  |
|  | rs16910208 | 11806921 |  | 1036 | 412 | 32 | 0.3 | 0.4 | 0.2 |  |
|  |  |  |  | 1042.3 | 399.5 | 38.3 |  |  |  |  |
|  | rs6485255 | 11814065 |  | 1467 | 13 | 0 | 0 | 0.1 | 0.1 |  |
|  |  |  |  | 1467 | 12.9 | 0 |  |  |  |  |
|  | rs6485257 | 11814890 |  | 989 | 444 | 47 | 0.1 | 0.2 | 0.2 |  |
|  |  |  |  | 990.9 | 440.2 | 48.9 |  |  |  |  |
| 11p15.3 | USP47 | 11819546 | Start |  |  |  |  |  |  | ubiquitin specific peptidase 47 |
|  | rs10831686 | 11846330 |  | 375 | 736 | 369 | -0.1 | 0 | -1.8 |  |
|  |  |  |  | 373 | 740 | 367 |  |  |  |  |
|  | rs12418890 | 11849793 |  | 1017 | 422 | 41 | 0.1 | -10.7 | -10.5 |  |
|  |  |  |  | 1018.9 | 418.2 | 42.9 |  |  |  |  |
|  | rs4360694 | 11875274 |  | 692 | 524 | 264 | -15 | -14.9 | 0 |  |
|  |  |  |  | 614.9 | 678.1 | 186.9 |  |  |  |  |
|  | rs11022092 | 11931629 |  | 1478 | 2 | 0 | 0 | 0 | 0 |  |
|  |  |  |  | 1478 | 2 | 0 |  |  |  |  |
|  | USP47 | 11937448 | End |  |  |  |  |  |  |  |
| 11p15.2 | DKK3 | 11941119 | Start |  |  |  |  |  |  | dickkopf homolog 3 (Xenopus laevis) |
|  | DKK3 | 11987493 | End |  |  |  |  |  |  |  |
| 11p15.3 | MICAL2 | 12088714 | Start |  |  |  |  |  |  | microtubule associated monoxygenase, calponin and LIM domain containing 2 |
|  | MICAL2 | 12241908 | End |  |  |  |  |  |  |  |
| 11p15.1 | PTPN5 | 18706051 | Start |  |  |  |  |  |  | protein tyrosine phosphatase, non-receptor type 5 (striatum-enriched) |
|  | PTPN5 | 18769965 | End |  |  |  |  |  |  |  |
| 11p15.1 | LOC441592 | 18841672 | Start |  |  |  |  |  |  | similar to Mas-related G-protein coupled receptor member X1 (Sensory neuron-specific G-protein coupled receptor 4) |
|  | LOC441592 | 18842632 | End |  |  |  |  |  |  |  |
| 11p15.1 | LOC645397 | 18849107 | Start |  |  |  |  |  |  | similar to methylenetetrahydrofolate dehydrogenase (NADP+ dependent) 1-like |
|  | LOC645397 | 18851666 | End |  |  |  |  |  |  |  |
| 11p15.1 | LOC390098 | 18865050 | Start |  |  |  |  |  |  | similar to MAS-related GPR, member X2 |
|  | LOC390098 | 18866043 | End |  |  |  |  |  |  |  |
| 11p15.1 | LOC390099 | 18888554 | Start |  |  |  |  |  |  | similar to Sensory neuron-specific G-protein coupled receptor 2 |
|  | LOC390099 | 18889519 | End |  |  |  |  |  |  |  |
|  | rs1842908 | 18907713 |  | 1476 | 4 | 0 | 0 | 28.4 | 28.4 |  |
|  |  |  |  | 1476 | 4 | 0 |  |  |  |  |
|  | rs2015757 | 18909153 |  | 365 | 943 | 172 | 28.4 | 28.4 | 26.7 |  |
|  |  |  |  | 472.8 | 727.4 | 279.8 |  |  |  |  |
|  | rs4756976 | 18911384 |  | 1480 | 0 | 0 | 0 | 0 | 1.9 |  |
|  |  |  |  | 1480 | 0 | 0 |  |  |  |  |
| 11p15.1 | MRGPRX1 | 18911936 | Start |  |  |  |  |  |  | MAS-related GPR, member X1 |
|  | rs11024885 | 18912802 |  | 1459 | 21 | 0 | 0 | 1.9 | 1.7 |  |
|  |  |  |  | 1459.1 | 20.9 | 0.1 |  |  |  |  |
|  | rs3858489 | 18913106 |  | 555 | 742 | 183 | 2 | 1.8 | 3 |  |
|  |  |  |  | 579.4 | 693.2 | 207.4 |  |  |  |  |
| 11p15.1 | LOC729352 | 18913114 | Start |  |  |  |  |  |  | hypothetical protein LOC729352 |
|  | MRGPRX1 | 18913125 | End |  |  |  |  |  |  |  |
|  | rs2004384 | 18913266 |  | 556 | 739 | 185 | 1.8 | 2.8 | 2.6 |  |
|  |  |  |  | 578.8 | 693.5 | 207.8 |  |  |  |  |
|  | rs4512797 | 18913740 |  | 1251 | 229 | 0 | 0.7 | 2.9 | 3.9 |  |
|  |  |  |  | 1259.9 | 211.3 | 8.9 |  |  |  |  |
|  | rs1842914 | 18913773 |  | 582 | 727 | 171 | 1.7 | 2.6 | 2.9 |  |
|  |  |  |  | 604 | 682.9 | 193 |  |  |  |  |
|  | rs1872021 | 18914462 |  | 536 | 771 | 173 | 4.1 | 3.8 | 3.8 |  |
|  |  |  |  | 573.8 | 695.5 | 210.8 |  |  |  |  |
|  | rs7107749 | 18915821 |  | 441 | 810 | 229 | 5 | 3.2 | 0 |  |
|  |  |  |  | 483.6 | 724.8 | 271.6 |  |  |  |  |
|  | rs4757737 | 18916600 |  | 548 | 753 | 179 | 2.7 | 0 | 0 |  |
|  |  |  |  | 577.5 | 694 | 208.5 |  |  |  |  |
|  | LOC729352 | 18918200 | End |  |  |  |  |  |  |  |
| 11p15.1 | LOC645415 | 18928570 | Start |  |  |  |  |  |  | similar to Sensory neuron-specific G-protein coupled receptor 2 |
|  | LOC645415 | 18936006 | End |  |  |  |  |  |  |  |
| 11p15.1 | LOC390101 | 18949859 | Start |  |  |  |  |  |  | similar to Sensory neuron-specific G-protein coupled receptor 2 |
|  | LOC390101 | 18955193 | End |  |  |  |  |  |  |  |
| 11p15.1 | MRGPRX2 | 19032578 | Start |  |  |  |  |  |  | MAS-related GPR, member X2 |
|  | MRGPRX2 | 19038804 | End |  |  |  |  |  |  |  |
| 11p15.1 | ZDHHC13 | 19095268 | Start |  |  |  |  |  |  | zinc finger, DHHC-type containing 13 |
|  | ZDHHC13 | 19154543 | End |  |  |  |  |  |  |  |
|  | rs4606498 | 81172161 |  | 643 | 665 | 172 | 0 | -0.2 | -0.2 |  |
|  |  |  |  | 643 | 665.1 | 172 |  |  |  |  |
|  | rs7927911 | 81173907 |  | 915 | 491 | 74 | -0.2 | -0.2 | -0.3 |  |
|  |  |  |  | 910 | 501.1 | 69 |  |  |  |  |
|  | rs7928108 | 81174055 |  | 915 | 491 | 74 | -0.2 | -0.3 | -0.3 |  |
|  |  |  |  | 910 | 501.1 | 69 |  |  |  |  |
|  | rs11232863 | 81175527 |  | 1479 | 0 | 1 | -0.8 | -0.8 | -7.3 |  |
|  |  |  |  | 1478 | 2 | 0 |  |  |  |  |
|  | rs11232864 | 81175555 |  | 1480 | 0 | 0 | 0 | -7.1 | -5.7 |  |
|  |  |  |  | 1480 | 0 | 0 |  |  |  |  |
|  | rs7944055 | 81181573 |  | 1149 | 260 | 71 | -7.1 | -5.7 | -5.8 |  |
|  |  |  |  | 1105.3 | 347.4 | 27.3 |  |  |  |  |
|  | rs7947005 | 81181640 |  | 1145 | 274 | 61 | -4.7 | -5 | -12.8 |  |
|  |  |  |  | 1110.5 | 343 | 26.5 |  |  |  |  |
|  | rs11232869 | 81183111 |  | 1216 | 245 | 19 | -0.3 | -4.9 | -10.4 |  |
|  |  |  |  | 1210.5 | 255.9 | 13.5 |  |  |  |  |
|  | rs4944354 | 81193598 |  | 813 | 518 | 149 | -4 | -10 | -0.5 |  |
|  |  |  |  | 776.5 | 591 | 112.5 |  |  |  |  |
|  | rs10792597 | 81194806 |  | 1065 | 333 | 82 | -5.5 | 0 | 0 |  |
|  |  |  |  | 1024.7 | 413.6 | 41.7 |  |  |  |  |
|  | rs4944357 | 81202309 |  | 632 | 690 | 158 | 0.8 | 0.7 | 0.1 |  |
|  |  |  |  | 645 | 664.1 | 171 |  |  |  |  |
|  | rs11607556 | 81203885 |  | 633 | 689 | 158 | 0.7 | 0.1 | 0 |  |
|  |  |  |  | 645.6 | 663.8 | 170.6 |  |  |  |  |
|  | rs11232882 | 81204221 |  | 1004 | 426 | 50 | -0.1 | 0 | 0 |  |
|  |  |  |  | 1000.7 | 432.5 | 46.7 |  |  |  |  |
| 11q25 | NCAPD3 | 133527547 | Start |  |  |  |  |  |  | non-SMC condensin II complex, subunit D3 |
|  | NCAPD3 | 133599636 | End |  |  |  |  |  |  |  |
| 11q25 | VPS26B | 133599771 | Start |  |  |  |  |  |  | vacuolar protein sorting 26 homolog B (S. pombe) |
|  | VPS26B | 133622896 | End |  |  |  |  |  |  |  |
| 11q25 | THYN1 | 133623383 | Start |  |  |  |  |  |  | thymocyte nuclear protein 1 |
|  | THYN1 | 133628470 | End |  |  |  |  |  |  |  |
| 11q25 | ACAD8 | 133628644 | Start |  |  |  |  |  |  | acyl-Coenzyme A dehydrogenase family, member 8 |
|  | ACAD8 | 133640959 | End |  |  |  |  |  |  |  |
| 11q25 | GLB1L3 | 133652266 | Start |  |  |  |  |  |  | galactosidase, beta 1 like 3 |
|  | GLB1L3 | 133694667 | End |  |  |  |  |  |  |  |
| 11q25 | LOC89944 | 133707016 | Start |  |  |  |  |  |  | hypothetical protein BC008326 |
| 11q25 | LOC100130507 | 133713735 | Start |  |  |  |  |  |  | hypothetical protein LOC100130507 |
|  | LOC100130507 | 133714512 | End |  |  |  |  |  |  |  |
|  | LOC89944 | 133751428 | End |  |  |  |  |  |  |  |
| 11q25 | B3GAT1 | 133753608 | Start |  |  |  |  |  |  | beta-1,3-glucuronyltransferase 1 (glucuronosyltransferase P) |
|  | rs1866769 | 133779886 |  | 915 | 499 | 66 | 0.1 | 0 | 0 |  |
|  |  |  |  | 916.3 | 496.5 | 67.3 |  |  |  |  |
|  | rs1866768 | 133779920 |  | 922 | 492 | 66 | 0 | 0 | 0.2 |  |
|  |  |  |  | 921.8 | 492.5 | 65.8 |  |  |  |  |
|  | B3GAT1 | 133787022 | End |  |  |  |  |  |  |  |
|  | rs1561613 | 133799781 |  | 1187 | 280 | 13 | 0.2 | 0.1 | 0.2 |  |
|  |  |  |  | 1189.8 | 274.4 | 15.8 |  |  |  |  |
|  | rs4264165 | 133816011 |  | 799 | 575 | 106 | -0.1 | 0 | -11.1 |  |
|  |  |  |  | 797.6 | 577.8 | 104.6 |  |  |  |  |
|  | rs11223816 | 133820709 |  | 616 | 680 | 184 | 0.1 | -20.1 | -5.5 |  |
|  |  |  |  | 617.5 | 677 | 185.5 |  |  |  |  |
|  | rs10894818 | 133821432 |  | 690 | 401 | 389 | -57.2 | -27 | -25.4 |  |
|  |  |  |  | 535.8 | 709.4 | 234.8 |  |  |  |  |
|  | rs10128652 | 133824928 |  | 765 | 590 | 125 | -0.3 | -0.2 | 0.7 |  |
|  |  |  |  | 759.2 | 601.6 | 119.2 |  |  |  |  |
|  | rs11223827 | 133831546 |  | 733 | 610 | 137 | -0.2 | 0.8 | -1.1 |  |
|  |  |  |  | 728 | 620 | 132 |  |  |  |  |
|  | rs4592433 | 133853131 |  | 490 | 744 | 246 | 0.7 | -3.3 | -2.4 |  |
|  |  |  |  | 502.1 | 719.9 | 258.1 |  |  |  |  |
|  | rs11223858 | 133855317 |  | 884 | 419 | 177 | -15.3 | -10.2 | -10.5 |  |
|  |  |  |  | 807.9 | 571.1 | 100.9 |  |  |  |  |
|  | rs7925572 | 133857095 |  | 1225 | 255 | 0 | 0.9 | 0.2 | 0.1 |  |
|  |  |  |  | 1236 | 233 | 11 |  |  |  |  |
|  | rs7119746 | 133861143 |  | 1034 | 407 | 39 | 0 | -0.1 | -0.2 |  |
|  |  |  |  | 1034.7 | 405.5 | 39.7 |  |  |  |  |
|  | rs11602971 | 133861915 |  | 1225 | 243 | 12 | 0 | 0 | -0.2 |  |
|  |  |  |  | 1225 | 242.9 | 12 |  |  |  |  |
|  | rs11223867 | 133867348 |  | 1238 | 232 | 10 | 0 | -0.7 | 0 |  |
|  |  |  |  | 1238.7 | 230.5 | 10.7 |  |  |  |  |
|  | rs4937902 | 133875643 |  | 1279 | 177 | 24 | -1.7 | 0 | 0 |  |
|  |  |  |  | 1263.6 | 207.9 | 8.6 |  |  |  |  |
| 11q25 | LOC729302 | 134046530 | Start |  |  |  |  |  |  | hypothetical protein LOC729302 |
|  | LOC729302 | 134049481 | End |  |  |  |  |  |  |  |
| 12p11.21 | ASSP14 | 33029940 | Start |  |  |  |  |  |  | argininosuccinate synthetase pseudogene 14 |
|  | ASSP14 | 33031370 | End |  |  |  |  |  |  |  |
|  | rs10459094 | 33167151 |  | 1299 | 175 | 6 | 0 | -0.1 | 0 |  |
|  |  |  |  | 1298.9 | 175.2 | 5.9 |  |  |  |  |
|  | rs1445311 | 33171126 |  | 1296 | 176 | 8 | -0.1 | -0.1 | -0.6 |  |
|  |  |  |  | 1294.2 | 179.5 | 6.2 |  |  |  |  |
|  | rs10844479 | 33171601 |  | 1475 | 5 | 0 | 0 | -0.5 | -0.5 |  |
|  |  |  |  | 1475 | 5 | 0 |  |  |  |  |
|  | rs16920643 | 33172361 |  | 916 | 485 | 79 | -0.5 | -0.5 | -0.6 |  |
|  |  |  |  | 906.8 | 503.3 | 69.8 |  |  |  |  |
|  | rs4931059 | 33172827 |  | 1475 | 5 | 0 | 0 | -0.1 | -0.1 |  |
|  |  |  |  | 1475 | 5 | 0 |  |  |  |  |
|  | rs7973393 | 33173493 |  | 1302 | 171 | 7 | -0.1 | -0.1 | -1.4 |  |
|  |  |  |  | 1300.8 | 173.4 | 5.8 |  |  |  |  |
|  | rs1445314 | 33176320 |  | 1477 | 3 | 0 | 0 | -4.7 | -32.2 |  |
|  |  |  |  | 1477 | 3 | 0 |  |  |  |  |
|  | rs10772040 | 33193705 |  | 1222 | 210 | 48 | -4.8 | -32 | -33.2 |  |
|  |  |  |  | 1189.8 | 274.4 | 15.8 |  |  |  |  |
|  | rs983985 | 33194560 |  | 715 | 481 | 284 | -23.9 | -25.5 | -4.3 |  |
|  |  |  |  | 616.9 | 677.2 | 185.9 |  |  |  |  |
|  | rs2018498 | 33195133 |  | 713 | 471 | 296 | -27.3 | -5.1 | -2.5 |  |
|  |  |  |  | 607.9 | 681.3 | 190.9 |  |  |  |  |
|  | rs1500075 | 33203464 |  | 391 | 729 | 360 | -0.2 | -0.1 | -1.7 |  |
|  |  |  |  | 385.7 | 739.7 | 354.7 |  |  |  |  |
|  | rs10219460 | 33219659 |  | 1274 | 200 | 6 | 0.1 | -1.4 | -1.6 |  |
|  |  |  |  | 1275.6 | 196.8 | 7.6 |  |  |  |  |
|  | rs1379534 | 33224334 |  | 971 | 432 | 77 | -1.5 | -1.7 | -2.3 |  |
|  |  |  |  | 952 | 470 | 58 |  |  |  |  |
|  | rs12297690 | 33225221 |  | 1303 | 171 | 6 | 0 | -1 | -1 |  |
|  |  |  |  | 1302.7 | 171.7 | 5.7 |  |  |  |  |
|  | rs1445313 | 33225528 |  | 1012 | 408 | 60 | -0.9 | -0.9 | -1.1 |  |
|  |  |  |  | 999.1 | 433.8 | 47.1 |  |  |  |  |
|  | rs4931700 | 33233844 |  | 1012 | 408 | 60 | -0.9 | -1.1 | -0.9 |  |
|  |  |  |  | 999.1 | 433.8 | 47.1 |  |  |  |  |
|  | rs1457672 | 33234180 |  | 798 | 552 | 130 | -1.3 | -1.1 | 0 |  |
|  |  |  |  | 779.4 | 589.2 | 111.4 |  |  |  |  |
|  | rs2085508 | 33234603 |  | 921 | 481 | 78 | -0.5 | 0 | 0 |  |
|  |  |  |  | 911.5 | 499.9 | 68.5 |  |  |  |  |
| 12p11.1 | SYT10 | 33419615 | Start |  |  |  |  |  |  | synaptotagmin X |
|  | SYT10 | 33484021 | End |  |  |  |  |  |  |  |
| 12q23.3 | BTBD11 | 106236327 | Start |  |  |  |  |  |  | BTB (POZ) domain containing 11 |
|  | rs4964217 | 106408792 |  | 1159 | 298 | 23 | -0.2 | -2.2 | -2.3 |  |
|  |  |  |  | 1156 | 304 | 20 |  |  |  |  |
|  | rs7962232 | 106411481 |  | 630 | 621 | 229 | -3.1 | -2.3 | -1.6 |  |
|  |  |  |  | 597.7 | 685.7 | 196.7 |  |  |  |  |
|  | rs12300726 | 106411686 |  | 849 | 521 | 110 | -1.2 | -0.7 | -1.7 |  |
|  |  |  |  | 831.8 | 555.5 | 92.8 |  |  |  |  |
|  | rs12300822 | 106411734 |  | 1110 | 344 | 26 | 0 | -1.7 | -1.5 |  |
|  |  |  |  | 1110.5 | 343 | 26.5 |  |  |  |  |
|  | rs11835314 | 106412032 |  | 631 | 620 | 229 | -3.2 | -2.1 | -2.2 |  |
|  |  |  |  | 598.3 | 685.4 | 196.3 |  |  |  |  |
|  | rs11113364 | 106415129 |  | 1161 | 293 | 26 | -0.3 | -2.3 | -2.3 |  |
|  |  |  |  | 1155.1 | 304.8 | 20.1 |  |  |  |  |
|  | rs759519 | 106416291 |  | 630 | 619 | 231 | -3.3 | -2.5 | -2.5 |  |
|  |  |  |  | 596.4 | 686.2 | 197.4 |  |  |  |  |
|  | rs7133143 | 106416590 |  | 1163 | 289 | 28 | -0.5 | -2.5 | -3.8 |  |
|  |  |  |  | 1155.1 | 304.8 | 20.1 |  |  |  |  |
|  | rs1861586 | 106416608 |  | 630 | 619 | 231 | -3.3 | -8.3 | -3.9 |  |
|  |  |  |  | 596.4 | 686.2 | 197.4 |  |  |  |  |
|  | rs4964596 | 106420101 |  | 861 | 414 | 205 | -21 | -12.9 | 0 |  |
|  |  |  |  | 770.7 | 594.6 | 114.7 |  |  |  |  |
|  | rs10492161 | 106420510 |  | 1164 | 287 | 29 | -0.6 | 0 | 0 |  |
|  |  |  |  | 1155.1 | 304.8 | 20.1 |  |  |  |  |
|  | BTBD11 | 106577549 | End |  |  |  |  |  |  |  |
| 12q23.3 | PWP1 | 106603720 | Start |  |  |  |  |  |  | PWP1 homolog (S. cerevisiae) |
|  | PWP1 | 106630387 | End |  |  |  |  |  |  |  |
| 12q24.13 | RPH3A | 111714125 | Start |  |  |  |  |  |  | rabphilin 3A homolog (mouse) |
|  | RPH3A | 111819631 | End |  |  |  |  |  |  |  |
|  | rs10850094 | 111823283 |  | 596 | 715 | 169 | 1.3 | 1.1 | 1.4 |  |
|  |  |  |  | 614.3 | 678.4 | 187.3 |  |  |  |  |
|  | rs10774674 | 111823894 |  | 597 | 709 | 174 | 0.9 | 1.3 | 1.3 |  |
|  |  |  |  | 611.7 | 679.6 | 188.7 |  |  |  |  |
|  | rs10850098 | 111824778 |  | 557 | 740 | 183 | 1.9 | 1.8 | 1.8 |  |
|  |  |  |  | 580.6 | 692.7 | 206.6 |  |  |  |  |
|  | rs10774676 | 111825127 |  | 558 | 740 | 182 | 1.9 | 1.8 | 1.8 |  |
|  |  |  |  | 581.9 | 692.2 | 205.9 |  |  |  |  |
|  | rs4766663 | 111825694 |  | 557 | 740 | 183 | 1.9 | 1.8 | 1.8 |  |
|  |  |  |  | 580.6 | 692.7 | 206.6 |  |  |  |  |
|  | rs6489868 | 111826412 |  | 558 | 739 | 183 | 1.8 | 1.8 | 1.8 |  |
|  |  |  |  | 581.3 | 692.5 | 206.3 |  |  |  |  |
|  | rs4766673 | 111827881 |  | 557 | 740 | 183 | 1.9 | 1.8 | 1.8 |  |
|  |  |  |  | 580.6 | 692.7 | 206.6 |  |  |  |  |
|  | rs4766674 | 111827921 |  | 560 | 737 | 183 | 1.7 | 1.8 | 1.9 |  |
|  |  |  |  | 582.5 | 692 | 205.5 |  |  |  |  |
|  | rs4766675 | 111828173 |  | 557 | 740 | 183 | 1.9 | 1.9 | 0 |  |
|  |  |  |  | 580.6 | 692.7 | 206.6 |  |  |  |  |
|  | rs4766676 | 111828301 |  | 557 | 742 | 181 | 2 | 0 | 0 |  |
|  |  |  |  | 581.9 | 692.2 | 205.9 |  |  |  |  |
| 12q24.1 | OAS1 | 111829122 | Start |  |  |  |  |  |  | 2',5'-oligoadenylate synthetase 1, 40/46kDa |
|  | OAS1 | 111842095 | End |  |  |  |  |  |  |  |
| 12q24.2 | OAS3 | 111860632 | Start |  |  |  |  |  |  | 2'-5'-oligoadenylate synthetase 3, 100kDa |
|  | OAS3 | 111895437 | End |  |  |  |  |  |  |  |
| 12q24.2 | OAS2 | 111900657 | Start |  |  |  |  |  |  | 2'-5'-oligoadenylate synthetase 2, 69/71kDa |
|  | OAS2 | 111933911 | End |  |  |  |  |  |  |  |
| 12q24.13 | DTX1 | 111980045 | Start |  |  |  |  |  |  | deltex homolog 1 (Drosophila) |
| 12q24.13 | LOC100129447 | 112012653 | Start |  |  |  |  |  |  | hypothetical protein LOC100129447 |
|  | LOC100129447 | 112013102 | End |  |  |  |  |  |  |  |
|  | DTX1 | 112020216 | End |  |  |  |  |  |  |  |
| 12q23-q24 | RASAL1 | 112021701 | Start |  |  |  |  |  |  | RAS protein activator like 1 (GAP1 like) |
|  | RASAL1 | 112058404 | End |  |  |  |  |  |  |  |
| 14q11.2 | P704P | 19057551 | Start |  |  |  |  |  |  | prostate-specific P704P |
|  | P704P | 19090272 | End |  |  |  |  |  |  |  |
| 14q11.1-q11.2 | LOC642850 | 19091205 | Start |  |  |  |  |  |  | similar to positive cofactor 2, glutamine/Q-rich-associated protein isoform b |
|  | LOC642850 | 19144901 | End |  |  |  |  |  |  |  |
| 14q11.2 | NEK2P | 19166779 | Start |  |  |  |  |  |  | NEK2 pseudogene |
|  | NEK2P | 19169042 | End |  |  |  |  |  |  |  |
| 14q11.2 | LOC400174 | 19206828 | Start |  |  |  |  |  |  | similar to single stranded DNA binding protein 3 |
|  | LOC400174 | 19208002 | End |  |  |  |  |  |  |  |
| 14q11.2 | USP10P1 | 19215649 | Start |  |  |  |  |  |  | ubiquitin specific peptidase 10 pseudogene 1 |
|  | USP10P1 | 19219046 | End |  |  |  |  |  |  |  |
| 14q11.2 | LOC254398 | 19226260 | Start |  |  |  |  |  |  | similar to Oligophrenin 1 |
|  | LOC254398 | 19230470 | End |  |  |  |  |  |  |  |
| 14q11.2 | OR11H8P | 19250735 | Start |  |  |  |  |  |  | olfactory receptor, family 11, subfamily H, member 8 pseudogene |
|  | OR11H8P | 19252115 | End |  |  |  |  |  |  |  |
| 14q11.2 | OR11K2P | 19271245 | Start |  |  |  |  |  |  | olfactory receptor, family 11, subfamily K, member 2 pseudogene |
|  | OR11K2P | 19272392 | End |  |  |  |  |  |  |  |
|  | rs4983173 | 19272965 |  | 934 | 510 | 36 | 1.8 | 1.5 | 4.5 |  |
|  |  |  |  | 955.2 | 467.6 | 57.2 |  |  |  |  |
| 14q11.2 | OR4Q3 | 19285427 | Start |  |  |  |  |  |  | olfactory receptor, family 4, subfamily Q, member 3 |
|  | OR4Q3 | 19286368 | End |  |  |  |  |  |  |  |
| 14q11.2 | OR4H12P | 19297909 | Start |  |  |  |  |  |  | olfactory receptor, family 4, subfamily H, member 12 pseudogene |
|  | OR4H12P | 19298787 | End |  |  |  |  |  |  |  |
|  | rs4562981 | 19309086 |  | 959 | 478 | 43 | 0.6 | 10 | 10.3 |  |
|  |  |  |  | 969.7 | 456.5 | 53.7 |  |  |  |  |
| 14q11.2 | OR4M1 | 19318322 | Start |  |  |  |  |  |  | olfactory receptor, family 4, subfamily M, member 1 |
|  | OR4M1 | 19319263 | End |  |  |  |  |  |  |  |
| 14q11.2 | OR4N1P | 19334241 | Start |  |  |  |  |  |  | olfactory receptor, family 4, subfamily N, member 1 pseudogene |
|  | OR4N1P | 19335389 | End |  |  |  |  |  |  |  |
|  | rs4412905 | 19336854 |  | 570 | 813 | 97 | 14.1 | 14.2 | 4.6 |  |
|  |  |  |  | 644.3 | 664.4 | 171.3 |  |  |  |  |
|  | rs4080788 | 19337785 |  | 1278 | 202 | 0 | 0.5 | 0.3 | 3.6 |  |
|  |  |  |  | 1284.9 | 188.2 | 6.9 |  |  |  |  |
|  | rs1780870 | 19362325 |  | 1040 | 403 | 37 | 0.1 | 3.2 | 3.4 |  |
|  |  |  |  | 1041.4 | 400.1 | 38.4 |  |  |  |  |
|  | rs10130102 | 19362693 |  | 717 | 716 | 47 | 10.9 | 9.6 | 20.7 |  |
|  |  |  |  | 780.8 | 588.3 | 110.8 |  |  |  |  |
| 14q11.2 | OR4N2 | 19365448 | Start |  |  |  |  |  |  | olfactory receptor, family 4, subfamily N, member 2 |
|  | OR4N2 | 19366371 | End |  |  |  |  |  |  |  |
|  | rs4473104 | 19380763 |  | 1011 | 455 | 14 | 2.5 | 28.7 | 19 |  |
|  |  |  |  | 1036.4 | 404.2 | 39.4 |  |  |  |  |
|  | rs2318527 | 19381928 |  | 317 | 1020 | 143 | 50.7 | 35.3 | 25.4 |  |
|  |  |  |  | 462.1 | 729.8 | 288.1 |  |  |  |  |
| 14q11.2 | OR4K6P | 19385266 | Start |  |  |  |  |  |  | olfactory receptor, family 4, subfamily K, member 6 pseudogene |
|  | OR4K6P | 19386328 | End |  |  |  |  |  |  |  |
|  | rs1686549 | 19402695 |  | 748 | 711 | 21 | 14.9 | 22.4 | 23 |  |
|  |  |  |  | 822.8 | 561.4 | 95.8 |  |  |  |  |
| 14q11.2 | OR4K3P | 19405996 | Start |  |  |  |  |  |  | olfactory receptor, family 4, subfamily K, member 3 pseudogene |
|  | rs1780941 | 19406967 |  | 446 | 918 | 116 | 28.3 | 26.8 | 18.8 |  |
|  |  |  |  | 553.4 | 703.2 | 223.4 |  |  |  |  |
|  | OR4K3P | 19407342 | End |  |  |  |  |  |  |  |
|  | rs1686539 | 19411485 |  | 538 | 867 | 75 | 24.7 | 20.3 | 19.9 |  |
|  |  |  |  | 637.7 | 667.6 | 174.7 |  |  |  |  |
| 14q11.2 | OR4K2 | 19414267 | Start |  |  |  |  |  |  | olfactory receptor, family 4, subfamily K, member 2 |
|  | OR4K2 | 19415211 | End |  |  |  |  |  |  |  |
|  | rs1780934 | 19416412 |  | 696 | 751 | 33 | 16.6 | 16.3 | 16.2 |  |
|  |  |  |  | 775.8 | 591.5 | 112.8 |  |  |  |  |
|  | rs1319956 | 19417051 |  | 689 | 754 | 37 | 16.2 | 16.5 | 12 |  |
|  |  |  |  | 767.8 | 596.4 | 115.8 |  |  |  |  |
|  | rs1319954 | 19417404 |  | 693 | 754 | 33 | 16.9 | 12.3 | 18.9 |  |
|  |  |  |  | 773.6 | 592.8 | 113.6 |  |  |  |  |
|  | rs1780930 | 19417621 |  | 830 | 630 | 20 | 8.9 | 16.6 | 16.7 |  |
|  |  |  |  | 885.8 | 518.3 | 75.8 |  |  |  |  |
|  | rs8013148 | 19420550 |  | 272 | 958 | 250 | 29.1 | 28.4 | 26 |  |
|  |  |  |  | 381.1 | 739.8 | 359.1 |  |  |  |  |
|  | rs1780927 | 19424016 |  | 599 | 843 | 38 | 27.4 | 24.9 | 23.6 |  |
|  |  |  |  | 703.7 | 633.7 | 142.7 |  |  |  |  |
| 14q11.2 | OR4K4P | 19443611 | Start |  |  |  |  |  |  | olfactory receptor, family 4, subfamily K, member 4 pseudogene |
|  | rs1780909 | 19444485 |  | 673 | 786 | 21 | 23 | 21.7 | 11.5 |  |
|  |  |  |  | 767.8 | 596.4 | 115.8 |  |  |  |  |
|  | OR4K4P | 19444743 | End |  |  |  |  |  |  |  |
|  | rs2318498 | 19447566 |  | 1426 | 54 | 0 | 0.1 | 1.6 | 14.2 |  |
|  |  |  |  | 1426.5 | 53 | 0.5 |  |  |  |  |
|  | rs1632089 | 19453422 |  | 1162 | 318 | 0 | 1.6 | 15.7 | 5 |  |
|  |  |  |  | 1179.1 | 283.8 | 17.1 |  |  |  |  |
| 14q11.2 | OR4K5 | 19458606 | Start |  |  |  |  |  |  | olfactory receptor, family 4, subfamily K, member 5 |
|  | OR4K5 | 19459577 | End |  |  |  |  |  |  |  |
|  | rs1686588 | 19459714 |  | 259 | 974 | 247 | 33.3 | 7.4 | 6.6 |  |
|  |  |  |  | 376 | 740 | 364 |  |  |  |  |
| 14q11.2 | OR4K1 | 19473666 | Start |  |  |  |  |  |  | olfactory receptor, family 4, subfamily K, member 1 |
|  | OR4K1 | 19474601 | End |  |  |  |  |  |  |  |
|  | rs7146334 | 19483641 |  | 795 | 663 | 22 | 10.8 | 7.8 | 15.1 |  |
|  |  |  |  | 857.4 | 538.1 | 84.4 |  |  |  |  |
|  | rs6572904 | 19487234 |  | 801 | 633 | 46 | 5.4 | 13.7 | 8.2 |  |
|  |  |  |  | 843.8 | 547.4 | 88.8 |  |  |  |  |
|  | rs2635568 | 19489099 |  | 241 | 1001 | 238 | 41.2 | 31.4 | 0.2 |  |
|  |  |  |  | 371.5 | 740 | 368.5 |  |  |  |  |
|  | rs10151880 | 19489537 |  | 545 | 805 | 130 | 9.9 | -3.5 | 0.3 |  |
|  |  |  |  | 606.6 | 681.8 | 191.6 |  |  |  |  |
|  | rs4060079 | 19489770 |  | 694 | 483 | 303 | -26 | 0.4 | 0.3 |  |
|  |  |  |  | 591.3 | 688.4 | 200.3 |  |  |  |  |
|  | rs2635576 | 19489991 |  | 242 | 1005 | 233 | 42.5 | 30.1 | 6.9 |  |
|  |  |  |  | 374.5 | 740 | 365.5 |  |  |  |  |
|  | rs3916621 | 19492423 |  | 449 | 888 | 143 | 20 | 8.2 | 8.2 |  |
|  |  |  |  | 538.8 | 708.4 | 232.8 |  |  |  |  |
| 14q11.2 | OR4K16P | 19494446 | Start |  |  |  |  |  |  | olfactory receptor, family 4, subfamily K, member 16 pseudogene |
|  | OR4K16P | 19495653 | End |  |  |  |  |  |  |  |
|  | rs10141075 | 19495751 |  | 548 | 690 | 242 | -0.5 | -0.5 | -0.5 |  |
|  |  |  |  | 538.8 | 708.4 | 232.8 |  |  |  |  |
|  | rs11850906 | 19496544 |  | 549 | 690 | 241 | -0.5 | -0.5 | -0.4 |  |
|  |  |  |  | 540 | 708 | 232 |  |  |  |  |
|  | rs7159122 | 19502641 |  | 548 | 690 | 242 | -0.5 | -0.4 | -0.4 |  |
|  |  |  |  | 538.8 | 708.4 | 232.8 |  |  |  |  |
|  | rs7151469 | 19502828 |  | 548 | 691 | 241 | -0.4 | -0.4 | 0.2 |  |
|  |  |  |  | 539.4 | 708.2 | 232.4 |  |  |  |  |
|  | rs6572987 | 19502884 |  | 548 | 690 | 242 | -0.5 | 0.2 | 0.2 |  |
|  |  |  |  | 538.8 | 708.4 | 232.8 |  |  |  |  |
| 14q11.2 | OR4K15 | 19513518 | Start |  |  |  |  |  |  | olfactory receptor, family 4, subfamily K, member 15 |
|  | OR4K15 | 19514564 | End |  |  |  |  |  |  |  |
|  | rs1953978 | 19525147 |  | 1207 | 273 | 0 | 1.1 | 1.1 | 0.7 |  |
|  |  |  |  | 1219.6 | 247.8 | 12.6 |  |  |  |  |
| 14q11.2 | OR4Q2P | 19540062 | Start |  |  |  |  |  |  | olfactory receptor, family 4, subfamily Q, member 2 pseudogene |
|  | OR4Q2P | 19541192 | End |  |  |  |  |  |  |  |
| 14q11.2 | OR4K14 | 19552260 | Start |  |  |  |  |  |  | olfactory receptor, family 4, subfamily K, member 14 |
|  | OR4K14 | 19553192 | End |  |  |  |  |  |  |  |
|  | rs1953998 | 19553467 |  | 1011 | 437 | 32 | 0.6 | 0.6 | 0 |  |
|  |  |  |  | 1021.4 | 416.2 | 42.4 |  |  |  |  |
|  | rs2185443 | 19556947 |  | 1254 | 214 | 12 | -0.1 | 0 | 0 |  |
|  |  |  |  | 1251.6 | 218.9 | 9.6 |  |  |  |  |
| 14q11.2 | OR4K13 | 19571843 | Start |  |  |  |  |  |  | olfactory receptor, family 4, subfamily K, member 13 |
|  | OR4K13 | 19572757 | End |  |  |  |  |  |  |  |
| 14q11.2 | OR4U1P | 19581815 | Start |  |  |  |  |  |  | olfactory receptor, family 4, subfamily U, member 1 pseudogene |
|  | OR4U1P | 19582954 | End |  |  |  |  |  |  |  |
| 14q11.2 | OR4L1 | 19598044 | Start |  |  |  |  |  |  | olfactory receptor, family 4, subfamily L, member 1 |
|  | OR4L1 | 19598982 | End |  |  |  |  |  |  |  |
| 14q11.2 | OR4T1P | 19631254 | Start |  |  |  |  |  |  | olfactory receptor, family 4, subfamily T, member 1 pseudogene |
|  | OR4T1P | 19632374 | End |  |  |  |  |  |  |  |
| 14q11.2 | OR4K17 | 19655406 | Start |  |  |  |  |  |  | olfactory receptor, family 4, subfamily K, member 17 |
|  | OR4K17 | 19656437 | End |  |  |  |  |  |  |  |
| 14q11.2 | OR4N5 | 19681735 | Start |  |  |  |  |  |  | olfactory receptor, family 4, subfamily N, member 5 |
|  | OR4N5 | 19682661 | End |  |  |  |  |  |  |  |
| 14q11.2 | OR11G1P | 19717127 | Start |  |  |  |  |  |  | olfactory receptor, family 11, subfamily G, member 1 pseudogene |
|  | OR11G1P | 19718277 | End |  |  |  |  |  |  |  |
| 14p13 | OR11P1P | 19721824 | Start |  |  |  |  |  |  | olfactory receptor, family 11, subfamily P, member 1 pseudogene |
|  | OR11P1P | 19722397 | End |  |  |  |  |  |  |  |
| 14q11.2 | OR11G2 | 19735335 | Start |  |  |  |  |  |  | olfactory receptor, family 11, subfamily G, member 2 |
|  | OR11G2 | 19736372 | End |  |  |  |  |  |  |  |
| 14q11.2 | OR11H5P | 19747196 | Start |  |  |  |  |  |  | olfactory receptor, family 11, subfamily H, member 5 pseudogene |
|  | OR11H5P | 19748294 | End |  |  |  |  |  |  |  |
| 14q32.33 | IGHVII-33-1 | 105894366 | Start |  |  |  |  |  |  | immunoglobulin heavy variable (II)-33-1 |
|  | IGHVII-33-1 | 105894639 | End |  |  |  |  |  |  |  |
| 14q32.33 | IGHV3-33-2 | 105896074 | Start |  |  |  |  |  |  | immunoglobulin heavy variable 3-33-2 |
|  | IGHV3-33-2 | 105896522 | End |  |  |  |  |  |  |  |
| 14q32.33 | IGHV4-34 | 105900637 | Start |  |  |  |  |  |  | immunoglobulin heavy variable 4-34 |
|  | IGHV4-34 | 105901069 | End |  |  |  |  |  |  |  |
| 14q32.33 | IGHV7-34-1 | 105904276 | Start |  |  |  |  |  |  | immunoglobulin heavy variable 7-34-1 |
|  | IGHV7-34-1 | 105904709 | End |  |  |  |  |  |  |  |
| 14q32.33 | IGHV3-35 | 105916366 | Start |  |  |  |  |  |  | immunoglobulin heavy variable 3-35 |
|  | IGHV3-35 | 105916819 | End |  |  |  |  |  |  |  |
| 14q32.33 | IGHV3-36 | 105919725 | Start |  |  |  |  |  |  | immunoglobulin heavy variable 3-36 |
|  | IGHV3-36 | 105920187 | End |  |  |  |  |  |  |  |
| 14q32.33 | IGHV3-37 | 105923622 | Start |  |  |  |  |  |  | immunoglobulin heavy variable 3-37 |
|  | IGHV3-37 | 105924071 | End |  |  |  |  |  |  |  |
| 14q32.33 | IGHV3-38 | 105937452 | Start |  |  |  |  |  |  | immunoglobulin heavy variable 3-38 |
|  | IGHV3-38 | 105937901 | End |  |  |  |  |  |  |  |
| 14q32.33 | IGHVIII-38-1 | 105944980 | Start |  |  |  |  |  |  | immunoglobulin heavy variable (III)-38-1 |
|  | IGHVIII-38-1 | 105945272 | End |  |  |  |  |  |  |  |
| 14q32.33 | IGHV4-39 | 105948662 | Start |  |  |  |  |  |  | immunoglobulin heavy variable 4-39 |
|  | IGHV4-39 | 105949100 | End |  |  |  |  |  |  |  |
| 14q32.33 | IGHV7-40 | 105952309 | Start |  |  |  |  |  |  | immunoglobulin heavy variable 7-40 |
|  | IGHV7-40 | 105952511 | End |  |  |  |  |  |  |  |
| 14q32.33 | IGHVII-40-1 | 105967890 | Start |  |  |  |  |  |  | immunoglobulin heavy variable (II)-40-1 |
|  | IGHVII-40-1 | 105967966 | End |  |  |  |  |  |  |  |
| 14q32.33 | IGHV3-41 | 105970085 | Start |  |  |  |  |  |  | immunoglobulin heavy variable 3-41 |
|  | IGHV3-41 | 105970539 | End |  |  |  |  |  |  |  |
| 14q32.33 | IGHV3-42 | 105990224 | Start |  |  |  |  |  |  | immunoglobulin heavy variable 3-42 |
|  | IGHV3-42 | 105990658 | End |  |  |  |  |  |  |  |
| 14q32.33 | IGHV3-43 | 105997232 | Start |  |  |  |  |  |  | immunoglobulin heavy variable 3-43 |
|  | IGHV3-43 | 105997689 | End |  |  |  |  |  |  |  |
| 14q32.33 | IGHVII-43-1 | 105999760 | Start |  |  |  |  |  |  | immunoglobulin heavy variable (II)-43-1 |
|  | IGHVII-43-1 | 105999968 | End |  |  |  |  |  |  |  |
| 14q32.33 | IGHVIII-44 | 106005268 | Start |  |  |  |  |  |  | immunoglobulin heavy variable (III)-44 |
|  | IGHVIII-44 | 106005444 | End |  |  |  |  |  |  |  |
| 14q32.33 | IGHVIV-44-1 | 106016394 | Start |  |  |  |  |  |  | immunoglobulin heavy variable (IV)-44-1 |
|  | IGHVIV-44-1 | 106016822 | End |  |  |  |  |  |  |  |
| 14q32.33 | IGHVII-44-2 | 106021204 | Start |  |  |  |  |  |  | immunoglobulin heavy variable (II)-44-2 |
|  | IGHVII-44-2 | 106021440 | End |  |  |  |  |  |  |  |
| 14q32.33 | IGHV1-45 | 106033974 | Start |  |  |  |  |  |  | immunoglobulin heavy variable 1-45 |
|  | IGHV1-45 | 106034411 | End |  |  |  |  |  |  |  |
| 14q32.33 | IGHV1-46 | 106038092 | Start |  |  |  |  |  |  | immunoglobulin heavy variable 1-46 |
|  | IGHV1-46 | 106038529 | End |  |  |  |  |  |  |  |
| 14q32.33 | IGHVII-46-1 | 106042815 | Start |  |  |  |  |  |  | immunoglobulin heavy variable (II)-46-1 |
|  | IGHVII-46-1 | 106043103 | End |  |  |  |  |  |  |  |
| 14q32.33 | IGHV3-47 | 106045551 | Start |  |  |  |  |  |  | immunoglobulin heavy variable 3-47 |
|  | IGHV3-47 | 106046006 | End |  |  |  |  |  |  |  |
| 14q32.33 | IGHVIII-47-1 | 106058180 | Start |  |  |  |  |  |  | immunoglobulin heavy variable (III)-47-1 |
|  | IGHVIII-47-1 | 106058483 | End |  |  |  |  |  |  |  |
| 14q32.33 | IGHV3-48 | 106064857 | Start |  |  |  |  |  |  | immunoglobulin heavy variable 3-48 |
|  | IGHV3-48 | 106065312 | End |  |  |  |  |  |  |  |
| 14q32.33 | IGHV3-49 | 106083981 | Start |  |  |  |  |  |  | immunoglobulin heavy variable 3-49 |
|  | IGHV3-49 | 106084442 | End |  |  |  |  |  |  |  |
| 14q32.33 | IGHVII-49-1 | 106091361 | Start |  |  |  |  |  |  | immunoglobulin heavy variable (II)-49-1 |
|  | IGHVII-49-1 | 106091631 | End |  |  |  |  |  |  |  |
| 14q32.33 | IGHV3-50 | 106093133 | Start |  |  |  |  |  |  | immunoglobulin heavy variable 3-50 |
|  | IGHV3-50 | 106093588 | End |  |  |  |  |  |  |  |
| 14q32.33 | IGHV5-51 | 106105772 | Start |  |  |  |  |  |  | immunoglobulin heavy variable 5-51 |
|  | IGHV5-51 | 106106207 | End |  |  |  |  |  |  |  |
| 14q32.33 | IGHVIII-51-1 | 106110533 | Start |  |  |  |  |  |  | immunoglobulin heavy variable (III)-51-1 |
|  | IGHVIII-51-1 | 106110839 | End |  |  |  |  |  |  |  |
| 14q32.33 | IGHVII-51-2 | 106111743 | Start |  |  |  |  |  |  | immunoglobulin heavy variable (II)-51-2 |
|  | IGHVII-51-2 | 106112005 | End |  |  |  |  |  |  |  |
| 14q32.33 | IGHV3-52 | 106113405 | Start |  |  |  |  |  |  | immunoglobulin heavy variable 3-52 |
|  | IGHV3-52 | 106113855 | End |  |  |  |  |  |  |  |
| 14q32.33 | IGHV3-53 | 106119715 | Start |  |  |  |  |  |  | immunoglobulin heavy variable 3-53 |
|  | IGHV3-53 | 106120165 | End |  |  |  |  |  |  |  |
| 14q32.33 | IGHVII-53-1 | 106126712 | Start |  |  |  |  |  |  | immunoglobulin heavy variable (II)-53-1 |
|  | IGHVII-53-1 | 106126981 | End |  |  |  |  |  |  |  |
| 14q32.33 | IGHV3-54 | 106128393 | Start |  |  |  |  |  |  | immunoglobulin heavy variable 3-54 |
|  | IGHV3-54 | 106128840 | End |  |  |  |  |  |  |  |
| 14q32.33 | IGHV4-55 | 106133171 | Start |  |  |  |  |  |  | immunoglobulin heavy variable 4-55 |
|  | IGHV4-55 | 106133606 | End |  |  |  |  |  |  |  |
| 14q32.33 | IGHV7-56 | 106136817 | Start |  |  |  |  |  |  | immunoglobulin heavy variable 7-56 |
|  | IGHV7-56 | 106137251 | End |  |  |  |  |  |  |  |
| 14q32.33 | IGHV3-57 | 106145967 | Start |  |  |  |  |  |  | immunoglobulin heavy variable 3-57 |
|  | IGHV3-57 | 106146272 | End |  |  |  |  |  |  |  |
| 14q32.33 | IGHV1-58 | 106149416 | Start |  |  |  |  |  |  | immunoglobulin heavy variable 1-58 |
|  | IGHV1-58 | 106149853 | End |  |  |  |  |  |  |  |
| 14q32.33 | IGHV4-59 | 106154299 | Start |  |  |  |  |  |  | immunoglobulin heavy variable 4-59 |
|  | IGHV4-59 | 106154730 | End |  |  |  |  |  |  |  |
| 14q32.33 | IGHV3-60 | 106158247 | Start |  |  |  |  |  |  | immunoglobulin heavy variable 3-60 |
|  | IGHV3-60 | 106158703 | End |  |  |  |  |  |  |  |
| 14q32.33 | IGHVII-60-1 | 106164755 | Start |  |  |  |  |  |  | immunoglobulin heavy variable (II)-60-1 |
|  | IGHVII-60-1 | 106165023 | End |  |  |  |  |  |  |  |
| 14q32.33 | IGHV4-61 | 106166169 | Start |  |  |  |  |  |  | immunoglobulin heavy variable 4-61 |
|  | IGHV4-61 | 106166606 | End |  |  |  |  |  |  |  |
| 14q32.33 | IGHV3-62 | 106170178 | Start |  |  |  |  |  |  | immunoglobulin heavy variable 3-62 |
|  | IGHV3-62 | 106170633 | End |  |  |  |  |  |  |  |
| 14q32.33 | IGHVII-62-1 | 106177565 | Start |  |  |  |  |  |  | immunoglobulin heavy variable (II)-62-1 |
|  | IGHVII-62-1 | 106177837 | End |  |  |  |  |  |  |  |
| 14q32.33 | IGHV3-63 | 106179273 | Start |  |  |  |  |  |  | immunoglobulin heavy variable 3-63 |
|  | IGHV3-63 | 106179740 | End |  |  |  |  |  |  |  |
| 14q32.33 | LOC192130 | 106180527 | Start |  |  |  |  |  |  | golgi autoantigen, golgin subfamily a, 4 pseudogene |
|  | LOC192130 | 106181443 | End |  |  |  |  |  |  |  |
| 14q32.33 | IGHV3-64 | 106184784 | Start |  |  |  |  |  |  | immunoglobulin heavy variable 3-64 |
|  | IGHV3-64 | 106185239 | End |  |  |  |  |  |  |  |
| 14q32.33 | IGHV3-65 | 106193099 | Start |  |  |  |  |  |  | immunoglobulin heavy variable 3-65 |
|  | IGHV3-65 | 106193593 | End |  |  |  |  |  |  |  |
| 14q32.33 | IGHVII-65-1 | 106198833 | Start |  |  |  |  |  |  | immunoglobulin heavy variable (II)-65-1 |
|  | IGHVII-65-1 | 106199105 | End |  |  |  |  |  |  |  |
| 14q32.33 | IGHV3-66 | 106202076 | Start |  |  |  |  |  |  | immunoglobulin heavy variable 3-66 |
|  | IGHV3-66 | 106202526 | End |  |  |  |  |  |  |  |
| 14q32.33 | IGHV1-67 | 106207665 | Start |  |  |  |  |  |  | immunoglobulin heavy variable 1-67 |
|  | IGHV1-67 | 106208104 | End |  |  |  |  |  |  |  |
| 14q32.33 | LOC192127 | 106209692 | Start |  |  |  |  |  |  | solute carrier family 20 (phosphate transporter), member 1 pseudogene |
|  | rs6576201 | 106210758 |  | 562 | 683 | 235 | -0.6 | -0.3 | 0 |  |
|  |  |  |  | 551.6 | 703.9 | 224.6 |  |  |  |  |
|  | rs9324092 | 106210868 |  | 526 | 708 | 246 | -0.1 | 0 | 0 |  |
|  |  |  |  | 523.2 | 713.5 | 243.2 |  |  |  |  |
|  | LOC192127 | 106211411 | End |  |  |  |  |  |  |  |
|  | rs17113284 | 106211538 |  | 810 | 570 | 100 | 0 | -0.1 | 22.6 |  |
|  |  |  |  | 810.2 | 569.7 | 100.2 |  |  |  |  |
| 14q32.33 | IGHVII-67-1 | 106213455 | Start |  |  |  |  |  |  | immunoglobulin heavy variable (II)-67-1 |
|  | IGHVII-67-1 | 106213604 | End |  |  |  |  |  |  |  |
| 14q32.33 | IGHVIII-67-2 | 106214164 | Start |  |  |  |  |  |  | immunoglobulin heavy variable (III)-67-2 |
|  | IGHVIII-67-2 | 106214262 | End |  |  |  |  |  |  |  |
|  | rs3858878 | 106215123 |  | 1114 | 343 | 23 | 0.1 | 57.9 | 58 |  |
|  |  |  |  | 1116.6 | 337.9 | 25.6 |  |  |  |  |
| 14q32.33 | IGHVIII-67-3 | 106219676 | Start |  |  |  |  |  |  | immunoglobulin heavy variable (III)-67-3 |
|  | IGHVIII-67-3 | 106219950 | End |  |  |  |  |  |  |  |
| 14q32.33 | IGHVIII-67-4 | 106222154 | Start |  |  |  |  |  |  | immunoglobulin heavy variable (III)-67-4 |
|  | IGHVIII-67-4 | 106222459 | End |  |  |  |  |  |  |  |
|  | rs7150397 | 106223861 |  | 293 | 1080 | 107 | 74 | 58.1 | 6.9 |  |
|  |  |  |  | 468.8 | 728.3 | 282.8 |  |  |  |  |
| 14q32.33 | IGHV1-68 | 106230869 | Start |  |  |  |  |  |  | immunoglobulin heavy variable 1-68 |
|  | IGHV1-68 | 106231348 | End |  |  |  |  |  |  |  |
|  | rs7146073 | 106231699 |  | 1114 | 344 | 22 | 0.2 | -0.6 | -0.6 |  |
|  |  |  |  | 1117.4 | 337.1 | 25.4 |  |  |  |  |
|  | rs17672538 | 106231839 |  | 824 | 532 | 124 | -1.6 | -1.6 | -1 |  |
|  |  |  |  | 802.8 | 574.5 | 102.8 |  |  |  |  |
|  | rs17113366 | 106238649 |  | 1466 | 14 | 0 | 0 | -0.1 | 15.4 |  |
|  |  |  |  | 1466 | 13.9 | 0 |  |  |  |  |
|  | rs3814922 | 106238827 |  | 518 | 714 | 248 | 0 | 15.7 | 15.8 |  |
|  |  |  |  | 517.3 | 715.4 | 247.3 |  |  |  |  |
| 14q32.32-q32.33 | IGHV1-69 | 106240974 | Start |  |  |  |  |  |  | immunoglobulin heavy variable 1-69 |
|  | IGHV1-69 | 106241412 | End |  |  |  |  |  |  |  |
|  | rs2904623 | 106246130 |  | 492 | 952 | 36 | 48.4 | 33.2 | 31.3 |  |
|  |  |  |  | 633.1 | 669.8 | 177.1 |  |  |  |  |
|  | rs10129255 | 106247258 |  | 811 | 571 | 98 | 0.1 | -0.6 | -0.2 |  |
|  |  |  |  | 812.4 | 568.3 | 99.4 |  |  |  |  |
| 14q32.33 | IGHV2-70 | 106249864 | Start |  |  |  |  |  |  | immunoglobulin heavy variable 2-70 |
|  | IGHV2-70 | 106250307 | End |  |  |  |  |  |  |  |
|  | rs2007467 | 106250892 |  | 810 | 538 | 132 | -1.9 | 0 | 0 |  |
|  |  |  |  | 786.6 | 584.7 | 108.6 |  |  |  |  |
|  | rs756581 | 106251269 |  | 1104 | 351 | 25 | 0.1 | 0.1 | 0 |  |
|  |  |  |  | 1106.2 | 346.7 | 27.2 |  |  |  |  |
| 14q32.33 | IGHV3-71 | 106254441 | Start |  |  |  |  |  |  | immunoglobulin heavy variable 3-71 |
|  | IGHV3-71 | 106254902 | End |  |  |  |  |  |  |  |
| 14q32.33 | IGHV3-72 | 106269975 | Start |  |  |  |  |  |  | immunoglobulin heavy variable 3-72 |
|  | IGHV3-72 | 106270436 | End |  |  |  |  |  |  |  |
| 14q32.33 | IGHV3-73 | 106281975 | Start |  |  |  |  |  |  | immunoglobulin heavy variable 3-73 |
|  | IGHV3-73 | 106282436 | End |  |  |  |  |  |  |  |
|  | rs4773949 | 106287351 |  | 1075 | 376 | 29 | 0.1 | 0 | 0 |  |
|  |  |  |  | 1077.8 | 370.4 | 31.8 |  |  |  |  |
| 14q32.33 | C14orf99 | 106288309 | Start |  |  |  |  |  |  | chromosome 14 open reading frame 99 |
|  | C14orf99 | 106289452 | End |  |  |  |  |  |  |  |
| 14q32.33 | IGHV3-74 | 106289719 | Start |  |  |  |  |  |  | immunoglobulin heavy variable 3-74 |
|  | IGHV3-74 | 106290174 | End |  |  |  |  |  |  |  |
| 14q32.33 | IGHVII-74-1 | 106300518 | Start |  |  |  |  |  |  | immunoglobulin heavy variable (II)-74-1 |
|  | IGHVII-74-1 | 106300686 | End |  |  |  |  |  |  |  |
| 14q32.33 | IGHV3-75 | 106302953 | Start |  |  |  |  |  |  | immunoglobulin heavy variable 3-75 |
|  | IGHV3-75 | 106303422 | End |  |  |  |  |  |  |  |
| 14q32.33 | IGHV3-76 | 106307134 | Start |  |  |  |  |  |  | immunoglobulin heavy variable 3-76 |
|  | IGHV3-76 | 106307583 | End |  |  |  |  |  |  |  |
| 14q32.33 | IGHVIII-76-1 | 106311035 | Start |  |  |  |  |  |  | immunoglobulin heavy variable (III)-76-1 |
|  | IGHVIII-76-1 | 106311341 | End |  |  |  |  |  |  |  |
| 14q32.33 | IGHV5-78 | 106330372 | Start |  |  |  |  |  |  | immunoglobulin heavy variable 5-78 |
|  | IGHV5-78 | 106330806 | End |  |  |  |  |  |  |  |
| 14q32.33 | IGHVII-78-1 | 106344881 | Start |  |  |  |  |  |  | immunoglobulin heavy variable (II)-78-1 |
|  | IGHVII-78-1 | 106345155 | End |  |  |  |  |  |  |  |
| 14q32.33 | IGHV3-79 | 106346898 | Start |  |  |  |  |  |  | immunoglobulin heavy variable 3-79 |
|  | IGHV3-79 | 106347349 | End |  |  |  |  |  |  |  |
| 14q32.33 | IGHV4-80 | 106352039 | Start |  |  |  |  |  |  | immunoglobulin heavy variable 4-80 |
|  | IGHV4-80 | 106352439 | End |  |  |  |  |  |  |  |
| 14q32.33 | IGHV7-81 | 106353835 | Start |  |  |  |  |  |  | immunoglobulin heavy variable 7-81 |
|  | IGHV7-81 | 106354271 | End |  |  |  |  |  |  |  |
| 14q32.33 | IGHVIII-82 | 106358805 | Start |  |  |  |  |  |  | immunoglobulin heavy variable (III)-82 |
|  | IGHVIII-82 | 106359096 | End |  |  |  |  |  |  |  |
| 15q11.1 | LOC729722 | 18337216 | Start |  |  |  |  |  |  | similar to ankyrin repeat domain 30A |
|  | LOC729722 | 18353201 | End |  |  |  |  |  |  |  |
| 15q11.2 | LOC651963 | 18429923 | Start |  |  |  |  |  |  | similar to Ig heavy chain V-I region HG3 precursor |
|  | LOC651963 | 18430368 | End |  |  |  |  |  |  |  |
| 15q11.2 | LOC646052 | 18432672 | Start |  |  |  |  |  |  | similar to Ig heavy chain V-I region V35 precursor |
|  | LOC646052 | 18438137 | End |  |  |  |  |  |  |  |
|  | rs7179358 | 18451755 |  | 552 | 690 | 238 | -0.4 | 0.1 | 1.5 |  |
|  |  |  |  | 543.7 | 706.7 | 229.7 |  |  |  |  |
| 15q11.2 | LOC646057 | 18452736 | Start |  |  |  |  |  |  | similar to Ig heavy chain V-III region VH26 precursor |
|  | LOC646057 | 18456699 | End |  |  |  |  |  |  |  |
| 15q11.2 | LOC646071 | 18511161 | Start |  |  |  |  |  |  | similar to a disintegrin and metallopeptidase domain 6 |
|  | LOC646071 | 18513705 | End |  |  |  |  |  |  |  |
| 15q11.2 | LOC646079 | 18530821 | Start |  |  |  |  |  |  | similar to breast cancer anti-estrogen resistance 1 |
|  | LOC646079 | 18539738 | End |  |  |  |  |  |  |  |
| 15q11.2 | LOC100132904 | 18587299 | Start |  |  |  |  |  |  | hypothetical protein LOC100132904 |
|  | LOC100132904 | 18588188 | End |  |  |  |  |  |  |  |
| 15q11.2 | LOC100130655 | 18605747 | Start |  |  |  |  |  |  | hypothetical LOC100130655 |
|  | LOC100130655 | 18608297 | End |  |  |  |  |  |  |  |
| 15q11.2 | LOC100132642 | 18609410 | Start |  |  |  |  |  |  | hypothetical LOC100132642 |
|  | LOC100132642 | 18610046 | End |  |  |  |  |  |  |  |
| 15q11.2 | LOC642311 | 18613637 | Start |  |  |  |  |  |  | similar to Ribosome beiogenesis protein BMS1 homolog |
|  | LOC642311 | 18624071 | End |  |  |  |  |  |  |  |
|  | rs4114033 | 18682601 |  | 1074 | 397 | 9 | 1.8 | 4.8 | 4.5 |  |
|  |  |  |  | 1094.1 | 356.8 | 29.1 |  |  |  |  |
| 15q11.2 | LOC646090 | 18693090 | Start |  |  |  |  |  |  | similar to rhophilin-like protein |
|  | LOC646090 | 18747329 | End |  |  |  |  |  |  |  |
| 15q11.2 | LOC646096 | 18748808 | Start |  |  |  |  |  |  | protein kinase CHK2-like |
|  | rs1670080 | 18753439 |  | 1053 | 426 | 1 | 3.5 | 3.2 | 6 |  |
|  |  |  |  | 1082.9 | 366.1 | 30.9 |  |  |  |  |
|  | LOC646096 | 18756847 | End |  |  |  |  |  |  |  |
| 15q11.2 | LOC100130364 | 18766866 | Start |  |  |  |  |  |  | hypothetical LOC100130364 |
|  | LOC100130364 | 18767499 | End |  |  |  |  |  |  |  |
| 15q11.2 | LOC100132392 | 18768876 | Start |  |  |  |  |  |  | hypothetical LOC100132392 |
|  | LOC100132392 | 18770436 | End |  |  |  |  |  |  |  |
|  | rs4114744 | 18782954 |  | 1478 | 0 | 2 | -1.3 | 4.1 | 1.9 |  |
|  |  |  |  | 1476 | 4 | 0 |  |  |  |  |
| 15q11.2 | LOC100133249 | 18795900 | Start |  |  |  |  |  |  | hypothetical protein LOC100133249 |
|  | LOC100133249 | 18796793 | End |  |  |  |  |  |  |  |
| 15q11.2 | LOC100132980 | 18810157 | Start |  |  |  |  |  |  | similar to Intraflagellar transport 81 homolog (Chlamydomonas) |
|  | LOC100132980 | 18824428 | End |  |  |  |  |  |  |  |
|  | rs6599965 | 18824589 |  | 978 | 493 | 9 | 4.3 | 2 | 3.3 |  |
|  |  |  |  | 1013.1 | 422.8 | 44.1 |  |  |  |  |
|  | rs4931971 | 18846941 |  | 1088 | 369 | 23 | 0.3 | 2.2 | 2 |  |
|  |  |  |  | 1094.1 | 356.8 | 29.1 |  |  |  |  |
| 15q11.2 | LOC283755 | 18848558 | Start |  |  |  |  |  |  | hypothetical protein LOC283755 |
| 15q11.2 | LOC283755 | 18848558 | Start |  |  |  |  |  |  | hypothetical protein LOC283755 |
| 15q11.2 | LOC646139 | 18970614 | Start |  |  |  |  |  |  | similar to hect domain and RLD 2 |
| 15q11.2 | LOC646139 | 18970614 | Start |  |  |  |  |  |  | similar to hect domain and RLD 2 |
|  | LOC283755 | 18971443 | End |  |  |  |  |  |  |  |
|  | LOC283755 | 18971443 | End |  |  |  |  |  |  |  |
|  | LOC646139 | 18995069 | End |  |  |  |  |  |  |  |
|  | LOC646139 | 18995069 | End |  |  |  |  |  |  |  |
| 15q11.2 | LOC100132727 | 18998028 | Start |  |  |  |  |  |  | hypothetical protein LOC100132727 |
| 15q11.2 | LOC100132727 | 18998028 | Start |  |  |  |  |  |  | hypothetical protein LOC100132727 |
|  | LOC100132727 | 18998856 | End |  |  |  |  |  |  |  |
|  | LOC100132727 | 18998856 | End |  |  |  |  |  |  |  |
| 15q11.2 | LOC727832 | 18999026 | Start |  |  |  |  |  |  | similar to cis-Golgi matrix protein GM130 |
| 15q11.2 | LOC727832 | 18999026 | Start |  |  |  |  |  |  | similar to cis-Golgi matrix protein GM130 |
|  | LOC727832 | 19007128 | End |  |  |  |  |  |  |  |
|  | LOC727832 | 19007128 | End |  |  |  |  |  |  |  |
| 15q11.2 | LOC729786 | 19027714 | Start |  |  |  |  |  |  | similar to golgi autoantigen, golgin subfamily a, 8A |
| 15q11.2 | LOC729786 | 19027714 | Start |  |  |  |  |  |  | similar to golgi autoantigen, golgin subfamily a, 8A |
|  | LOC729786 | 19041108 | End |  |  |  |  |  |  |  |
|  | LOC729786 | 19041108 | End |  |  |  |  |  |  |  |
| 15q11.2 | LOC646177 | 19058780 | Start |  |  |  |  |  |  | similar to chromosome 9 open reading frame 79 |
| 15q11.2 | LOC646177 | 19058780 | Start |  |  |  |  |  |  | similar to chromosome 9 open reading frame 79 |
|  | LOC646177 | 19064543 | End |  |  |  |  |  |  |  |
|  | LOC646177 | 19064543 | End |  |  |  |  |  |  |  |
| 15q11.2 | LOC401805 | 19092050 | Start |  |  |  |  |  |  | hypothetical gene supported by NM_144726 |
| 15q11.2 | LOC401805 | 19092050 | Start |  |  |  |  |  |  | hypothetical gene supported by NM_144726 |
|  | LOC401805 | 19093967 | End |  |  |  |  |  |  |  |
|  | LOC401805 | 19093967 | End |  |  |  |  |  |  |  |
|  | SNP_A-2255702 | 19111608 |  | 836 | 610 | 34 | 5.7 | 5 | 4.5 |  |
|  |  |  |  | 879.6 | 522.7 | 77.6 |  |  |  |  |
|  | rs8040821 | 19112164 |  | 1240 | 240 | 0 | 0.8 | 3.4 | 16.8 |  |
|  |  |  |  | 1249.7 | 220.5 | 9.7 |  |  |  |  |
|  | rs8040821 | 19112164 |  | 1240 | 240 | 0 | 0.8 | 3.4 | 16.8 |  |
|  |  |  |  | 1249.7 | 220.5 | 9.7 |  |  |  |  |
| 15q11-q13 | BCL8 | 19134811 | Start |  |  |  |  |  |  | B-cell CLL/lymphoma 8 |
| 15q11-q13 | BCL8 | 19134811 | Start |  |  |  |  |  |  | B-cell CLL/lymphoma 8 |
|  | rs6600068 | 19146832 |  | 940 | 522 | 18 | 4 | 21.2 | 18.7 |  |
|  |  |  |  | 974.6 | 452.8 | 52.6 |  |  |  |  |
|  | rs6600068 | 19146832 |  | 940 | 522 | 18 | 4 | 21.2 | 18.7 |  |
|  |  |  |  | 974.6 | 452.8 | 52.6 |  |  |  |  |
|  | rs4247069 | 19149205 |  | 645 | 821 | 14 | 29.3 | 18.7 | 17.1 |  |
|  |  |  |  | 752.8 | 605.5 | 121.8 |  |  |  |  |
|  | rs4247069 | 19149205 |  | 645 | 821 | 14 | 29.3 | 18.7 | 17.1 |  |
|  |  |  |  | 752.8 | 605.5 | 121.8 |  |  |  |  |
|  | rs939200 | 19164827 |  | 849 | 579 | 52 | 2.4 | 2 | 2 |  |
|  |  |  |  | 875.8 | 525.4 | 78.8 |  |  |  |  |
|  | rs939200 | 19164827 |  | 849 | 579 | 52 | 2.4 | 2 | 2 |  |
|  |  |  |  | 875.8 | 525.4 | 78.8 |  |  |  |  |
|  | rs6600090 | 19178339 |  | 1397 | 82 | 1 | 0 | 0 | 0.6 |  |
|  |  |  |  | 1397.2 | 81.6 | 1.2 |  |  |  |  |
|  | rs6600090 | 19178339 |  | 1397 | 82 | 1 | 0 | 0 | 0.6 |  |
|  |  |  |  | 1397.2 | 81.6 | 1.2 |  |  |  |  |
| 15q11.2 | LOC646214 | 19187488 | Start |  |  |  |  |  |  | similar to p21-activated kinase 2 |
| 15q11.2 | LOC646214 | 19187488 | Start |  |  |  |  |  |  | similar to p21-activated kinase 2 |
|  | LOC646214 | 19194116 | End |  |  |  |  |  |  |  |
|  | LOC646214 | 19194116 | End |  |  |  |  |  |  |  |
|  | rs4402527 | 19207088 |  | 1480 | 0 | 0 | 0 | 0.8 | -0.2 |  |
|  |  |  |  | 1480 | 0 | 0 |  |  |  |  |
|  | rs4402527 | 19207088 |  | 1480 | 0 | 0 | 0 | 0.8 | 0 |  |
|  |  |  |  | 1480 | 0 | 0 |  |  |  |  |
|  | BCL8 | 19221496 | End |  |  |  |  |  |  |  |
|  | BCL8 | 19221496 | End |  |  |  |  |  |  |  |
| 15q11.2 | LOC100133065 | 19238458 | Start |  |  |  |  |  |  | similar to hCG1734082 |
| 15q11.2 | LOC100133065 | 19238458 | Start |  |  |  |  |  |  | similar to hCG1734082 |
|  | LOC100133065 | 19240245 | End |  |  |  |  |  |  |  |
|  | LOC100133065 | 19240245 | End |  |  |  |  |  |  |  |
| 15q11.2 | DKFZP547L112 | 19250616 | Start |  |  |  |  |  |  | hypothetical protein DKFZp547L112 |
| 15q11.2 | DKFZP547L112 | 19250616 | Start |  |  |  |  |  |  | hypothetical protein DKFZp547L112 |
|  | DKFZP547L112 | 19266096 | End |  |  |  |  |  |  |  |
|  | DKFZP547L112 | 19266096 | End |  |  |  |  |  |  |  |
| 15q11.2 | LOC646243 | 19268721 | Start |  |  |  |  |  |  | similar to coxsackie virus and adenovirus receptor precursor |
| 15q11.2 | LOC646243 | 19268721 | Start |  |  |  |  |  |  | similar to coxsackie virus and adenovirus receptor precursor |
|  | LOC646243 | 19280268 | End |  |  |  |  |  |  |  |
|  | LOC646243 | 19280268 | End |  |  |  |  |  |  |  |
| 15q11.2 | A26B1 | 19305253 | Start |  |  |  |  |  |  | ANKRD26-like family B, member 1 |
| 15q11.2 | A26B1 | 19305253 | Start |  |  |  |  |  |  | ANKRD26-like family B, member 1 |
|  | A26B1 | 19336667 | End |  |  |  |  |  |  |  |
|  | A26B1 | 19336667 | End |  |  |  |  |  |  |  |
|  | rs3848222 | 19356830 |  | 1244 | 236 | 0 | 0.8 | -0.2 | 0 |  |
|  |  |  |  | 1253.4 | 217.2 | 9.4 |  |  |  |  |
|  | rs3848222 | 19356830 |  | 1244 | 236 | 0 | 0.8 | 0 | 0 |  |
|  |  |  |  | 1253.4 | 217.2 | 9.4 |  |  |  |  |
|  | rs2243568 | 19369108 |  | 901 | 496 | 83 | -0.5 | -0.2 | 2.9 |  |
|  |  |  |  | 892 | 513.9 | 74 |  |  |  |  |
| 15q11.2 | LOC440225 | 19370631 | Start |  |  |  |  |  |  | similar to Neurofibromin (Neurofibromatosis-related protein NF-1) |
| 15q11.2 | LOC440225 | 19370631 | Start |  |  |  |  |  |  | similar to Neurofibromin (Neurofibromatosis-related protein NF-1) |
|  | rs2251057 | 19385558 |  | 1197 | 273 | 10 | 0.3 | 6.8 | 6.9 |  |
|  |  |  |  | 1201.5 | 264 | 14.5 |  |  |  |  |
|  | rs7166137 | 19403945 |  | 832 | 635 | 13 | 10.5 | 10.6 | 17.1 |  |
|  |  |  |  | 892.8 | 513.4 | 73.8 |  |  |  |  |
|  | LOC440225 | 19405675 | End |  |  |  |  |  |  |  |
|  | LOC440225 | 19405675 | End |  |  |  |  |  |  |  |
| 15q11.2 | OR11J2P | 19430549 | Start |  |  |  |  |  |  | olfactory receptor, family 11, subfamily J, member 2 pseudogene |
| 15q11.2 | OR11J2P | 19430549 | Start |  |  |  |  |  |  | olfactory receptor, family 11, subfamily J, member 2 pseudogene |
|  | OR11J2P | 19431694 | End |  |  |  |  |  |  |  |
|  | OR11J2P | 19431694 | End |  |  |  |  |  |  |  |
| 15q11.2 | OR11J5P | 19439138 | Start |  |  |  |  |  |  | olfactory receptor, family 11, subfamily J, member 5 pseudogene |
| 15q11.2 | OR11J5P | 19439138 | Start |  |  |  |  |  |  | olfactory receptor, family 11, subfamily J, member 5 pseudogene |
|  | OR11J5P | 19440297 | End |  |  |  |  |  |  |  |
|  | OR11J5P | 19440297 | End |  |  |  |  |  |  |  |
| 15q11.2 | LOC727899 | 19449018 | Start |  |  |  |  |  |  | hypothetical protein LOC727899 |
| 15q11.2 | LOC727899 | 19449018 | Start |  |  |  |  |  |  | hypothetical protein LOC727899 |
|  | LOC727899 | 19461476 | End |  |  |  |  |  |  |  |
|  | LOC727899 | 19461476 | End |  |  |  |  |  |  |  |
|  | rs17134298 | 19464910 |  | 1478 | 2 | 0 | 0 | 24.2 | 43.8 |  |
|  |  |  |  | 1478 | 2 | 0 |  |  |  |  |
| 15q11.2 | LOC283804 | 19522590 | Start |  |  |  |  |  |  | similar to a disintegrin and metallopeptidase domain 6 |
| 15q11.2 | LOC283804 | 19522590 | Start |  |  |  |  |  |  | similar to a disintegrin and metallopeptidase domain 6 |
|  | LOC283804 | 19525134 | End |  |  |  |  |  |  |  |
|  | LOC283804 | 19525134 | End |  |  |  |  |  |  |  |
| 15q11.2 | LOC100133063 | 19542669 | Start |  |  |  |  |  |  | similar to breast cancer anti-estrogen resistance 1 |
| 15q11.2 | LOC100133063 | 19542669 | Start |  |  |  |  |  |  | similar to breast cancer anti-estrogen resistance 1 |
|  | LOC100133063 | 19551572 | End |  |  |  |  |  |  |  |
|  | LOC100133063 | 19551572 | End |  |  |  |  |  |  |  |
| 15q11.2 | LOC100132026 | 19598556 | Start |  |  |  |  |  |  | hypothetical protein LOC100132026 |
|  | LOC100132026 | 19599445 | End |  |  |  |  |  |  |  |
| 15q11.2 | LOC100132293 | 19617008 | Start |  |  |  |  |  |  | hypothetical LOC100132293 |
|  | LOC100132293 | 19619558 | End |  |  |  |  |  |  |  |
| 15q11.2 | LOC400968 | 19620671 | Start |  |  |  |  |  |  | hypothetical LOC400968 |
|  | LOC400968 | 19621307 | End |  |  |  |  |  |  |  |
| 15q11.2 | LOC727914 | 19624879 | Start |  |  |  |  |  |  | similar to Ribosome biogenesis protein BMS1 homolog |
|  | LOC727914 | 19635323 | End |  |  |  |  |  |  |  |
| 15q11.2 | OR11J1P | 19769501 | Start |  |  |  |  |  |  | olfactory receptor, family 11, subfamily J, member 1 pseudogene |
|  | OR11J1P | 19770660 | End |  |  |  |  |  |  |  |
| 15q11.2 | LOC727924 | 19779378 | Start |  |  |  |  |  |  | hypothetical protein LOC727924 |
|  | LOC727924 | 19791849 | End |  |  |  |  |  |  |  |
| 15q11.2 | OR11H3P | 19798765 | Start |  |  |  |  |  |  | olfactory receptor, family 11, subfamily H, member 3 pseudogene |
|  | OR11H3P | 19799911 | End |  |  |  |  |  |  |  |
| 15q11.2 | OR11K1P | 19820118 | Start |  |  |  |  |  |  | olfactory receptor, family 11, subfamily K, member 1 pseudogene |
|  | OR11K1P | 19821246 | End |  |  |  |  |  |  |  |
|  | rs1346662 | 19821421 |  | 684 | 782 | 14 | 24.1 | 43.4 | 38 |  |
|  |  |  |  | 780.8 | 588.3 | 110.8 |  |  |  |  |
|  | rs2880332 | 19830049 |  | 616 | 839 | 25 | 29.5 | 33.2 | 38.4 |  |
|  |  |  |  | 724.5 | 622 | 133.5 |  |  |  |  |
|  | rs7166438 | 19831773 |  | 751 | 716 | 13 | 17.1 | 21.5 | 46.8 |  |
|  |  |  |  | 831 | 556 | 93 |  |  |  |  |
|  | rs7166613 | 19831810 |  | 673 | 797 | 10 | 27 | 52.9 | 32.2 |  |
|  |  |  |  | 775.8 | 591.5 | 112.8 |  |  |  |  |
| 15q11.2 | OR4Q1P | 19833632 | Start |  |  |  |  |  |  | olfactory receptor, family 4, subfamily Q, member 1 pseudogene |
|  | OR4Q1P | 19834812 | End |  |  |  |  |  |  |  |
|  | rs4238548 | 19835428 |  | 577 | 884 | 19 | 38.4 | 24.3 | 22.8 |  |
|  |  |  |  | 701.6 | 634.8 | 143.6 |  |  |  |  |
| 15q11.2 | LOC652851 | 19845949 | Start |  |  |  |  |  |  | similar to Olfactory receptor 4H12 |
| 15q11.2 | OR4H6P | 19846219 | Start |  |  |  |  |  |  | olfactory receptor, family 4, subfamily H, member 6 pseudogene |
|  | OR4H6P | 19846855 | End |  |  |  |  |  |  |  |
|  | LOC652851 | 19846957 | End |  |  |  |  |  |  |  |
|  | rs4310812 | 19852603 |  | 1177 | 301 | 2 | 1.2 | 2.1 | 2.3 |  |
|  |  |  |  | 1190.7 | 273.6 | 15.7 |  |  |  |  |
| 15q11.2 | OR4M2 | 19869940 | Start |  |  |  |  |  |  | olfactory receptor, family 4, subfamily M, member 2 |
|  | OR4M2 | 19870881 | End |  |  |  |  |  |  |  |
|  | rs2082048 | 19876834 |  | 777 | 630 | 73 | 2.7 | 2.8 | 33.3 |  |
|  |  |  |  | 805.7 | 572.6 | 101.7 |  |  |  |  |
| 15q11.2 | OR4N4 | 19883837 | Start |  |  |  |  |  |  | olfactory receptor, family 4, subfamily N, member 4 |
|  | OR4N4 | 19884787 | End |  |  |  |  |  |  |  |
|  | rs1896867 | 19886462 |  | 1281 | 199 | 0 | 0.5 | 39.3 | 34.9 |  |
|  |  |  |  | 1287.7 | 185.6 | 6.7 |  |  |  |  |
|  | rs4779295 | 19911648 |  | 515 | 945 | 20 | 50.5 | 38.3 | 31.9 |  |
|  |  |  |  | 658.9 | 657.2 | 163.9 |  |  |  |  |
|  | rs11854399 | 19912458 |  | 845 | 622 | 13 | 9.7 | 28.8 | 56 |  |
|  |  |  |  | 902.9 | 506.1 | 70.9 |  |  |  |  |
| 15q11.2 | OR4N3P | 19914826 | Start |  |  |  |  |  |  | olfactory receptor, family 4, subfamily N, member 3 pseudogene |
|  | OR4N3P | 19915749 | End |  |  |  |  |  |  |  |
|  | rs1835208 | 19915944 |  | 601 | 856 | 23 | 32.7 | 80.2 | 48.7 |  |
|  |  |  |  | 715.4 | 627.1 | 137.4 |  |  |  |  |
|  | rs7161939 | 19922588 |  | 169 | 1159 | 152 | 104.5 | 58 | 48.7 |  |
|  |  |  |  | 378.5 | 739.9 | 361.5 |  |  |  |  |
| 15q11.2 | LOC388076 | 19941517 | Start |  |  |  |  |  |  | similar to ribosomal protein S8 |
|  | LOC388076 | 19942223 | End |  |  |  |  |  |  |  |
|  | rs10519379 | 19943075 |  | 1233 | 191 | 56 | -7.3 | 7.2 | 7.1 |  |
|  |  |  |  | 1192.5 | 272 | 15.5 |  |  |  |  |
|  | rs11635275 | 19943185 |  | 689 | 774 | 17 | 22.4 | 22.4 | 19.4 |  |
|  |  |  |  | 782.3 | 587.4 | 110.3 |  |  |  |  |
|  | rs8035298 | 19945295 |  | 1479 | 1 | 0 | 0 | 0.1 | 75.3 |  |
|  |  |  |  | 1479 | 1 | 0 |  |  |  |  |
| 15q11.2 | IGHV1OR15-1 | 19949604 | Start |  |  |  |  |  |  | immunoglobulin heavy variable 1/OR15-1 |
|  | rs2271569 | 19950116 |  | 1422 | 58 | 0 | 0.1 | 75.1 | 45.3 |  |
|  |  |  |  | 1422.6 | 56.9 | 0.6 |  |  |  |  |
|  | IGHV1OR15-1 | 19950239 | End |  |  |  |  |  |  |  |
| 15q11.2 | LOC646370 | 19967170 | Start |  |  |  |  |  |  | similar to Ig heavy chain V-I region V35 precursor |
|  | LOC646370 | 19967857 | End |  |  |  |  |  |  |  |
|  | rs1813939 | 19971289 |  | 333 | 1077 | 70 | 77.7 | 45.7 | 54.8 |  |
|  |  |  |  | 513.2 | 716.6 | 250.2 |  |  |  |  |
| 15q11.2 | LOC642131 | 19972818 | Start |  |  |  |  |  |  | similar to Ig heavy chain V-II region ARH-77 precursor |
|  | LOC642131 | 19974738 | End |  |  |  |  |  |  |  |
| 15q11.2 | LOC646379 | 19983953 | Start |  |  |  |  |  |  | similar to Ig heavy chain V-I region V35 precursor |
|  | rs2055220 | 19985717 |  | 1115 | 356 | 9 | 1.2 | 24 | 17.6 |  |
|  |  |  |  | 1129.6 | 326.7 | 23.6 |  |  |  |  |
|  | rs11633173 | 19988041 |  | 623 | 838 | 19 | 30.7 | 20.5 | 44.9 |  |
|  |  |  |  | 733.6 | 616.8 | 129.6 |  |  |  |  |
|  | rs1826882 | 19989036 |  | 894 | 532 | 54 | 1 | 39.9 | 25.5 |  |
|  |  |  |  | 909.2 | 501.6 | 69.2 |  |  |  |  |
|  | LOC646379 | 19991387 | End |  |  |  |  |  |  |  |
|  | rs1001444 | 20016405 |  | 325 | 1084 | 71 | 80 | 45.8 | 21.8 |  |
|  |  |  |  | 507.9 | 718.2 | 253.9 |  |  |  |  |
| 15q11.2 | LOC646396 | 20046847 | Start |  |  |  |  |  |  | similar to Zinc finger CCHC domain-containing protein 2 |
|  | rs1985933 | 20053967 |  | 1059 | 387 | 34 | 0 | 2.3 | 3.3 |  |
|  |  |  |  | 1060 | 385.1 | 35 |  |  |  |  |
|  | rs7166350 | 20059872 |  | 732 | 672 | 76 | 4.4 | 5.1 | 3 |  |
|  |  |  |  | 770.7 | 594.6 | 114.7 |  |  |  |  |
|  | LOC646396 | 20072491 | End |  |  |  |  |  |  |  |
|  | rs11259883 | 20079140 |  | 919 | 536 | 25 | 3.6 | 2 | 1.2 |  |
|  |  |  |  | 952 | 470 | 58 |  |  |  |  |
| 15q11.2 | LOC100130556 | 20241196 | Start |  |  |  |  |  |  | hypothetical LOC100130556 |
|  | LOC100130556 | 20242310 | End |  |  |  |  |  |  |  |
| 15q13-q14 | ABCB10P | 20243548 | Start |  |  |  |  |  |  | ATP-binding cassette, sub-family B (MDR/TAP), member 10 pseudogene |
|  | ABCB10P | 20244000 | End |  |  |  |  |  |  |  |
| 15q11.2 | LOC100132979 | 20253649 | Start |  |  |  |  |  |  | similar to FLJ32679 protein |
|  | LOC100132979 | 20267066 | End |  |  |  |  |  |  |  |
| 15q11.2 | LOC283767 | 20287610 | Start |  |  |  |  |  |  | FLJ40198 protein |
| 15q11.2 | LOC100131452 | 20295617 | Start |  |  |  |  |  |  | hypothetical protein LOC100131452 |
|  | LOC283767 | 20296164 | End |  |  |  |  |  |  |  |
|  | LOC100131452 | 20296446 | End |  |  |  |  |  |  |  |
| 15q11.2 | LOC100129539 | 20299406 | Start |  |  |  |  |  |  | similar to FLJ00287 protein |
|  | LOC100129539 | 20300689 | End |  |  |  |  |  |  |  |
| 15q11.2 | LOC729894 | 20328861 | Start |  |  |  |  |  |  | similar to Engulfment and cell motility protein 2 (CED-12 homolog A) |
|  | rs4405519 | 20329239 |  | 517 | 733 | 230 | 0.6 | 0.1 | 0.1 |  |
|  |  |  |  | 527.4 | 712.2 | 240.4 |  |  |  |  |
|  | rs12900257 | 20335459 |  | 783 | 585 | 112 | -0.1 | -0.1 | -0.1 |  |
|  |  |  |  | 781.6 | 587.9 | 110.6 |  |  |  |  |
|  | rs7359214 | 20335847 |  | 1344 | 132 | 4 | 0 | 0 | 0 |  |
|  |  |  |  | 1343.3 | 133.4 | 3.3 |  |  |  |  |
|  | LOC729894 | 20341338 | End |  |  |  |  |  |  |  |
|  | rs8036395 | 20343286 |  | 1480 | 0 | 0 | 0 | 0 | 0 |  |
|  |  |  |  | 1480 | 0 | 0 |  |  |  |  |
| 15q11.2 | TUBGCP5 | 20384836 | Start |  |  |  |  |  |  | tubulin, gamma complex associated protein 5 |
|  | TUBGCP5 | 20425332 | End |  |  |  |  |  |  |  |
| 15q11 | CYFIP1 | 20444125 | Start |  |  |  |  |  |  | cytoplasmic FMR1 interacting protein 1 |
|  | CYFIP1 | 20555044 | End |  |  |  |  |  |  |  |
| 15q25.2 | ADAMTSL3 | 82113842 | Start |  |  |  |  |  |  | ADAMTS-like 3 |
|  | rs10163186 | 82483240 |  | 894 | 509 | 77 | -0.1 | -0.1 | -0.2 |  |
|  |  |  |  | 891.3 | 514.5 | 74.3 |  |  |  |  |
|  | rs11259939 | 82483942 |  | 1480 | 0 | 0 | 0 | -0.1 | -0.1 |  |
|  |  |  |  | 1480 | 0 | 0 |  |  |  |  |
|  | rs17158441 | 82496114 |  | 1354 | 122 | 4 | -0.1 | -0.1 | 0 |  |
|  |  |  |  | 1352.9 | 124.3 | 2.9 |  |  |  |  |
|  | ADAMTSL3 | 82499598 | End |  |  |  |  |  |  |  |
| 15q25.2 | LOC642677 | 82512870 | Start |  |  |  |  |  |  | hypothetical LOC642677 |
|  | rs1818950 | 82513525 |  | 784 | 587 | 109 | 0 | 0 | 0 |  |
|  |  |  |  | 784.5 | 586.1 | 109.5 |  |  |  |  |
|  | LOC642677 | 82516571 | End |  |  |  |  |  |  |  |
| 15q25.2 | LOC727963 | 82539342 | Start |  |  |  |  |  |  | similar to elongation factor Tu GTP binding domain containing 1 |
|  | rs7178655 | 82555245 |  | 682 | 649 | 149 | 0.1 | -0.1 | 1.4 |  |
|  |  |  |  | 684.5 | 644 | 151.5 |  |  |  |  |
|  | LOC727963 | 82564540 | End |  |  |  |  |  |  |  |
| 15q25.2 | LOC648809 | 82572708 | Start |  |  |  |  |  |  | similar to elongation factor Tu GTP binding domain containing 1 |
|  | rs11638297 | 82573421 |  | 785 | 587 | 108 | 0 | 1.8 | 2.2 |  |
|  |  |  |  | 785.9 | 585.2 | 108.9 |  |  |  |  |
|  | LOC648809 | 82602209 | End |  |  |  |  |  |  |  |
|  | rs4842939 | 82618970 |  | 761 | 662 | 57 | 5.7 | 4.7 | 4.6 |  |
|  |  |  |  | 805.7 | 572.6 | 101.7 |  |  |  |  |
| 15q25.2 | LOC100132799 | 82630934 | Start |  |  |  |  |  |  | hypothetical protein LOC100132799 |
|  | LOC100132799 | 82633129 | End |  |  |  |  |  |  |  |
| 15q25.2 | LOC440300 | 82633719 | Start |  |  |  |  |  |  | similar to melanoma-associated chondroitin sulfate proteoglycan 4 |
|  | LOC440300 | 82657377 | End |  |  |  |  |  |  |  |
| 15q25.2 | LOC728764 | 82658602 | Start |  |  |  |  |  |  | similar to Dynamin-1 (D100) (Dynamin, brain) (B-dynamin) |
| 15q25.2 | LOC727994 | 82659568 | Start |  |  |  |  |  |  | similar to LOC388161 |
| 15q25.2 | LOC388152 | 82660151 | Start |  |  |  |  |  |  | hypothetical LOC388152 |
|  | LOC728764 | 82662767 | End |  |  |  |  |  |  |  |
|  | LOC727994 | 82662767 | End |  |  |  |  |  |  |  |
|  | LOC388152 | 82689960 | End |  |  |  |  |  |  |  |
| 15q25.2 | LOC161527 | 82695491 | Start |  |  |  |  |  |  | hypothetical protein LOC161527 |
|  | LOC161527 | 82707734 | End |  |  |  |  |  |  |  |
| 15q25.2 | LOC643707 | 82735249 | Start |  |  |  |  |  |  | golgi autoantigen, golgin subfamily a, 6 pseudogene |
|  | LOC643707 | 82741216 | End |  |  |  |  |  |  |  |
|  | rs4842947 | 82743638 |  | 881 | 532 | 67 | 0.4 | 9.9 | 10.4 |  |
|  |  |  |  | 888.9 | 516.1 | 74.9 |  |  |  |  |
| 15q25.2 | KIAA1920 | 82744017 | Start |  |  |  |  |  |  | KIAA1920 protein |
|  | KIAA1920 | 82757402 | End |  |  |  |  |  |  |  |
| 15q25.2 | LOC440302 | 82758326 | Start |  |  |  |  |  |  | similar to ubiquitin-conjugating enzyme E2Q 2 |
|  | LOC440302 | 82767450 | End |  |  |  |  |  |  |  |
| 15q25.2 | LOC100132084 | 82844619 | Start |  |  |  |  |  |  | hypothetical protein LOC100132084 |
|  | LOC100132084 | 82846616 | End |  |  |  |  |  |  |  |
|  | rs3883011 | 82889398 |  | 740 | 734 | 6 | 20.4 | 21.4 | 21.9 |  |
|  |  |  |  | 828 | 558 | 94 |  |  |  |  |
|  | rs3883013 | 82889661 |  | 740 | 740 | 0 | 22.5 | 22.9 | 7.9 |  |
|  |  |  |  | 832.5 | 555 | 92.5 |  |  |  |  |
|  | rs3883014 | 82889733 |  | 729 | 750 | 1 | 23.4 | 8.1 | 4.2 |  |
|  |  |  |  | 823.5 | 561 | 95.5 |  |  |  |  |
|  | rs11638788 | 82892291 |  | 713 | 617 | 150 | -0.4 | -0.1 | 0.1 |  |
|  |  |  |  | 705 | 632.9 | 142 |  |  |  |  |
|  | rs11635505 | 82898038 |  | 759 | 604 | 117 | 0.1 | 0.2 | 0.1 |  |
|  |  |  |  | 760.6 | 600.8 | 118.6 |  |  |  |  |
|  | rs1849287 | 82904075 |  | 879 | 532 | 69 | 0.3 | 0 | 0.5 |  |
|  |  |  |  | 885.8 | 518.3 | 75.8 |  |  |  |  |
|  | rs11638630 | 82911513 |  | 757 | 602 | 121 | 0 | 0.2 | 0 |  |
|  |  |  |  | 756.3 | 603.3 | 120.3 |  |  |  |  |
|  | rs7180051 | 82917298 |  | 1147 | 316 | 17 | 0.2 | -0.2 | -0.2 |  |
|  |  |  |  | 1150.7 | 308.6 | 20.7 |  |  |  |  |
| 15q25.2 | ZSCAN2 | 82945253 | Start |  |  |  |  |  |  | zinc finger and SCAN domain containing 2 |
|  | ZSCAN2 | 82967951 | End |  |  |  |  |  |  |  |
|  | rs17532346 | 82972499 |  | 762 | 588 | 130 | -0.4 | -0.4 | -0.4 |  |
|  |  |  |  | 753.5 | 605.1 | 121.5 |  |  |  |  |
|  | rs11634320 | 82973187 |  | 760 | 589 | 131 | -0.4 | -0.4 | 0 |  |
|  |  |  |  | 751.3 | 606.3 | 122.3 |  |  |  |  |
|  | rs2292463 | 82976754 |  | 444 | 713 | 323 | -0.6 | 0 | 0 |  |
|  |  |  |  | 433 | 735.1 | 312 |  |  |  |  |
| 15q25.2 | WDR73 | 82987003 | Start |  |  |  |  |  |  | WD repeat domain 73 |
|  | WDR73 | 82998525 | End |  |  |  |  |  |  |  |
| 15q22-qter | NMB | 82999364 | Start |  |  |  |  |  |  | neuromedin B |
|  | NMB | 83002806 | End |  |  |  |  |  |  |  |
| 15q25.3 | SEC11A | 83013779 | Start |  |  |  |  |  |  | SEC11 homolog A (S. cerevisiae) |
|  | SEC11A | 83060678 | End |  |  |  |  |  |  |  |
| 15q25.3 | ZNF592 | 83092822 | Start |  |  |  |  |  |  | zinc finger protein 592 |
|  | ZNF592 | 83150667 | End |  |  |  |  |  |  |  |
| 15q25.2 | ALPK3 | 83160915 | Start |  |  |  |  |  |  | alpha-kinase 3 |
|  | ALPK3 | 83217714 | End |  |  |  |  |  |  |  |
| 17q21.31 | LOC339192 | 40652465 | Start |  |  |  |  |  |  | hypothetical protein LOC339192 |
| 17q21 | FMNL1 | 40655075 | Start |  |  |  |  |  |  | formin-like 1 |
|  | LOC339192 | 40675042 | End |  |  |  |  |  |  |  |
|  | FMNL1 | 40680468 | End |  |  |  |  |  |  |  |
| 17q21.31 | C17orf46 | 40687543 | Start |  |  |  |  |  |  | chromosome 17 open reading frame 46 |
|  | C17orf46 | 40695262 | End |  |  |  |  |  |  |  |
| 17q21 | MAP3K14 | 40696271 | Start |  |  |  |  |  |  | mitogen-activated protein kinase kinase kinase 14 |
|  | MAP3K14 | 40750197 | End |  |  |  |  |  |  |  |
|  | rs4792855 | 40815480 |  | 405 | 746 | 329 | 0.2 | 0.3 | 0.3 |  |
|  |  |  |  | 409 | 738 | 333 |  |  |  |  |
|  | rs1230094 | 40825939 |  | 751 | 622 | 107 | 0.6 | 0.7 | 0.6 |  |
|  |  |  |  | 762.1 | 599.9 | 118.1 |  |  |  |  |
|  | rs732589 | 40826543 |  | 762 | 617 | 101 | 0.7 | 0.6 | 0.5 |  |
|  |  |  |  | 774.3 | 592.4 | 113.3 |  |  |  |  |
| 17q21.31 | ARHGAP27 | 40827058 | Start |  |  |  |  |  |  | Rho GTPase activating protein 27 |
|  | rs1230103 | 40841574 |  | 753 | 619 | 108 | 0.5 | 0.5 | 0.2 |  |
|  |  |  |  | 762.8 | 599.5 | 117.8 |  |  |  |  |
|  | rs12947718 | 40848884 |  | 977 | 458 | 45 | 0.3 | 0 | 0 |  |
|  |  |  |  | 982.7 | 446.5 | 50.7 |  |  |  |  |
|  | ARHGAP27 | 40858780 | End |  |  |  |  |  |  |  |
| 17q21.31 | LOC201175 | 40862501 | Start |  |  |  |  |  |  | hypothetical protein LOC201175 |
|  | LOC201175 | 40867570 | End |  |  |  |  |  |  |  |
| 17q21.31 | PLEKHM1 | 40869049 | Start |  |  |  |  |  |  | pleckstrin homology domain containing, family M (with RUN domain) member 1 |
|  | rs17631303 | 40872185 |  | 1036 | 399 | 45 | -0.2 | 0 | -0.1 |  |
|  |  |  |  | 1031.4 | 408.2 | 40.4 |  |  |  |  |
|  | rs3946526 | 40897439 |  | 980 | 455 | 45 | 0.3 | 0.1 | 49.4 |  |
|  |  |  |  | 985.2 | 444.7 | 50.2 |  |  |  |  |
|  | rs2078200 | 40897617 |  | 803 | 573 | 104 | 0 | 80.1 | 79.7 |  |
|  |  |  |  | 802 | 574.9 | 103 |  |  |  |  |
|  | PLEKHM1 | 40923893 | End |  |  |  |  |  |  |  |
| 17q21.31 | LOC644354 | 40934084 | Start |  |  |  |  |  |  | similar to Apoptosis-related protein 2 (APR-2) |
|  | LOC644354 | 40934428 | End |  |  |  |  |  |  |  |
| 17q21.31 | LRRC37A4 | 40939890 | Start |  |  |  |  |  |  | leucine rich repeat containing 37, member A4 (pseudogene) |
|  | LRRC37A4 | 40948305 | End |  |  |  |  |  |  |  |
|  | rs2696639 | 41006823 |  | 89 | 1312 | 79 | 193.6 | 193.6 | 191.3 |  |
|  |  |  |  | 375 | 740 | 365 |  |  |  |  |
|  | rs2696640 | 41007016 |  | 88 | 1312 | 80 | 193.6 | 192 | 192 |  |
|  |  |  |  | 374 | 740 | 366 |  |  |  |  |
|  | rs2693363 | 41007205 |  | 93 | 1307 | 80 | 190.3 | 192.6 | 192.6 |  |
|  |  |  |  | 376.5 | 739.9 | 363.5 |  |  |  |  |
|  | rs2693364 | 41007294 |  | 87 | 1314 | 79 | 195 | 195 | 153.4 |  |
|  |  |  |  | 374 | 740 | 366 |  |  |  |  |
|  | rs2693371 | 41011471 |  | 87 | 1314 | 79 | 195 | 153.4 | 153.4 |  |
|  |  |  |  | 374 | 740 | 366 |  |  |  |  |
|  | rs17642476 | 41012163 |  | 1298 | 182 | 0 | 0.4 | 153.4 | 75.8 |  |
|  |  |  |  | 1303.6 | 170.8 | 5.6 |  |  |  |  |
|  | rs2463520 | 41015138 |  | 87 | 1314 | 79 | 195 | 98.8 | 98.8 |  |
|  |  |  |  | 374 | 740 | 366 |  |  |  |  |
| 17q21.31 | LOC644157 | 41018375 | Start |  |  |  |  |  |  | similar to dead end homolog 1 |
|  | LOC644157 | 41020592 | End |  |  |  |  |  |  |  |
|  | rs2696425 | 41022689 |  | 879 | 524 | 77 | 0 | 0 | 0 |  |
|  |  |  |  | 879.6 | 522.7 | 77.6 |  |  |  |  |
| 17q21.31 | LOC644172 | 41033261 | Start |  |  |  |  |  |  | similar to C-jun-amino-terminal kinase-interacting protein 1 (JNK-interacting protein 1) (JIP-1) (JNK MAP kinase scaffold protein 1) (Islet-brain 1) (IB-1) (Mitogen-activated protein kinase 8-interacting protein 1) |
|  | LOC644172 | 41035447 | End |  |  |  |  |  |  |  |
| 17q21.31 | LOC644191 | 41041689 | Start |  |  |  |  |  |  | similar to 40S ribosomal protein S26 |
|  | LOC644191 | 41042466 | End |  |  |  |  |  |  |  |
|  | rs418891 | 41049321 |  | 880 | 524 | 76 | 0 | 0 | 0 |  |
|  |  |  |  | 881.2 | 521.6 | 77.2 |  |  |  |  |
| 17q21.31 | MGC57346 | 41069447 | Start |  |  |  |  |  |  | hypothetical LOC401884 |
|  | MGC57346 | 41071121 | End |  |  |  |  |  |  |  |
|  | rs413778 | 41072668 |  | 881 | 522 | 77 | 0 | 0 | 0 |  |
|  |  |  |  | 881.2 | 521.6 | 77.2 |  |  |  |  |
| 17q21.31 | C17orf69 | 41073731 | Start |  |  |  |  |  |  | chromosome 17 open reading frame 69 |
|  | C17orf69 | 41075613 | End |  |  |  |  |  |  |  |
|  | rs453997 | 41082844 |  | 881 | 522 | 77 | 0 | 0 | 0 |  |
|  |  |  |  | 881.2 | 521.6 | 77.2 |  |  |  |  |
|  | rs422112 | 41083920 |  | 881 | 522 | 77 | 0 | 0 | 0 |  |
|  |  |  |  | 881.2 | 521.6 | 77.2 |  |  |  |  |
|  | rs241033 | 41089766 |  | 882 | 521 | 77 | 0 | 0 | 0 |  |
|  |  |  |  | 882 | 521.1 | 77 |  |  |  |  |
|  | rs241032 | 41089928 |  | 881 | 522 | 77 | 0 | 0 | 0 |  |
|  |  |  |  | 881.2 | 521.6 | 77.2 |  |  |  |  |
| 17q12-q22 | CRHR1 | 41217449 | Start |  |  |  |  |  |  | corticotropin releasing hormone receptor 1 |
| 17q21.31 | LOC100131650 | 41258323 | Start |  |  |  |  |  |  | hypothetical protein LOC100131650 |
|  | LOC100131650 | 41258826 | End |  |  |  |  |  |  |  |
|  | CRHR1 | 41268973 | End |  |  |  |  |  |  |  |
| 17q21.31 | LOC100128977 | 41276797 | Start |  |  |  |  |  |  | similar to hCG1654542 |
| 17q21.31 | IMP5 | 41278053 | Start |  |  |  |  |  |  | intramembrane protease 5 |
|  | IMP5 | 41280107 | End |  |  |  |  |  |  |  |
|  | LOC100128977 | 41328988 | End |  |  |  |  |  |  |  |
|  | rs4490092 | 62030952 |  | 451 | 699 | 330 | -1.2 | -0.2 | 0 |  |
|  |  |  |  | 433 | 735.1 | 312 |  |  |  |  |
|  | rs1032343 | 62031073 |  | 811 | 557 | 112 | -0.5 | -0.1 | -0.1 |  |
|  |  |  |  | 802 | 574.9 | 103 |  |  |  |  |
|  | rs12965991 | 62044897 |  | 1312 | 165 | 3 | 0.1 | 0.1 | -0.3 |  |
|  |  |  |  | 1313.9 | 161.1 | 4.9 |  |  |  |  |
|  | rs12971105 | 62046043 |  | 1315 | 161 | 4 | 0.1 | -0.4 | -0.8 |  |
|  |  |  |  | 1315.8 | 159.4 | 4.8 |  |  |  |  |
|  | rs11664818 | 62053230 |  | 852 | 526 | 102 | -0.7 | -1.6 | -4.3 |  |
|  |  |  |  | 840 | 550 | 90 |  |  |  |  |
|  | rs12956627 | 62058576 |  | 1319 | 140 | 21 | -1.9 | -5.8 | -8.1 |  |
|  |  |  |  | 1303.6 | 170.8 | 5.6 |  |  |  |  |
|  | SNP_A-2053948 | 62059541 |  | 1243 | 198 | 39 | -3.5 | -5.3 | -20.3 |  |
|  |  |  |  | 1216.9 | 250.3 | 12.9 |  |  |  |  |
|  | rs1565516 | 62060664 |  | 1354 | 113 | 13 | -1.1 | -13.9 | -16.4 |  |
|  |  |  |  | 1344.3 | 132.5 | 3.3 |  |  |  |  |
|  | rs1873481 | 62060697 |  | 830 | 470 | 180 | -10.8 | -12.7 | -1.9 |  |
|  |  |  |  | 766.4 | 597.3 | 116.4 |  |  |  |  |
|  | rs9948507 | 62062341 |  | 704 | 541 | 235 | -10.1 | -1.6 | -1.3 |  |
|  |  |  |  | 641.7 | 665.7 | 172.7 |  |  |  |  |
|  | rs1907415 | 62063122 |  | 692 | 649 | 139 | 0.3 | 0.5 | -0.3 |  |
|  |  |  |  | 698.2 | 636.7 | 145.2 |  |  |  |  |
|  | rs902581 | 62063708 |  | 1369 | 109 | 2 | 0 | -1.3 | -0.4 |  |
|  |  |  |  | 1369.2 | 108.7 | 2.2 |  |  |  |  |
|  | rs902582 | 62063989 |  | 585 | 656 | 239 | -1.6 | -0.6 | -0.3 |  |
|  |  |  |  | 563.2 | 699.6 | 217.2 |  |  |  |  |
|  | rs7226966 | 62068324 |  | 701 | 641 | 138 | 0.2 | -0.3 | -0.3 |  |
|  |  |  |  | 705 | 632.9 | 142 |  |  |  |  |
|  | rs17076089 | 62069399 |  | 652 | 642 | 186 | -0.7 | -0.3 | 0 |  |
|  |  |  |  | 639.7 | 666.6 | 173.7 |  |  |  |  |
|  | rs10153423 | 62069508 |  | 701 | 642 | 137 | 0.2 | 0 | 0 |  |
|  |  |  |  | 705.7 | 632.5 | 141.7 |  |  |  |  |
